# Mechanism Inversion in Visible Light-Induced Photoclick Reactions

**DOI:** 10.1021/jacs.5c12759

**Published:** 2025-09-19

**Authors:** Youxin Fu, Jingwen Zhou, Xinyi Zou, Liuhui Shi, Xing Zhang, Anna M. Doze, Michiel F. Hilbers, Wybren Jan Buma, Jianyu Zhang, Ben L. Feringa

**Affiliations:** † College of Science, 74584 Nanjing Forestry University, Nanjing 210037, P.R. China; ‡ Centre for Systems Chemistry, Stratingh Institute for Chemistry, Faculty for Science and Engineering, 3647 University of Groningen, Nijenborgh 4, Groningen 9747 AG, The Netherlands; § Co-Innovation Center for Sustainable Forestry in Southern China, College of Ecology and Environment, 74584 Nanjing Forestry University, Nanjing 210037, China; ∥ Van’t Hoff Institute for Molecular Sciences, 1234 University of Amsterdam, Science Park 904, Amsterdam 1098 XH, The Netherlands; ⊥ Institute for Molecules and Materials, FELIX Laboratory, Radboud University, Toernooiveld 7c, Nijmegen 6525 ED, The Netherlands

## Abstract

Photoclick chemistry has emerged as a powerful methodology for achieving precise spatial and temporal control in photochemical transformations, enabling various applications ranging from surface functionalization, polymer conjugation, and photo-cross-linking to bioimaging and protein labeling. Despite significant advances, the absence of an unambiguous structure–property–mechanism relationship limits the design and application of photoclick systems in biological and material contexts. Herein, we report a novel strategy for tuning the reactivity and reaction mechanism of photoclick reactions of 9,10-phenanthrenequinone (**PQ**) with electron-rich alkenes (**ERA**s) through the design of 2,2’-substituted **PQ** derivatives incorporating electron-withdrawing groups (EWGs) and electron-donating groups (EDGs). Our experimental studies reveal that the reaction rate via a direct pathway involving the direct coupling between **PQ** and **ERA** gradually declines, while that of the triplet–triplet energy transfer-mediated pathway increases with the stepwise change of substitutions from EWGs to EDGs. Theoretical calculations and transient absorption spectroscopy measurements show that these observations can be traced back to the excited-state energy-level inversion between ^1^ *n*π* and ^1^ππ* states, which directly affects intersystem crossing yields. Furthermore, the reaction pathways can be modulated by changing the polarity of the solvent. These remarkable findings provide valuable mechanistic insights and establish a robust platform for the rational tuning of the reactivity and selectivity of **PQ–ERA** photoclick reactions using visible light while offering a unique strategy for the control of photochemical applications in complex environments.

## Graphical abstract

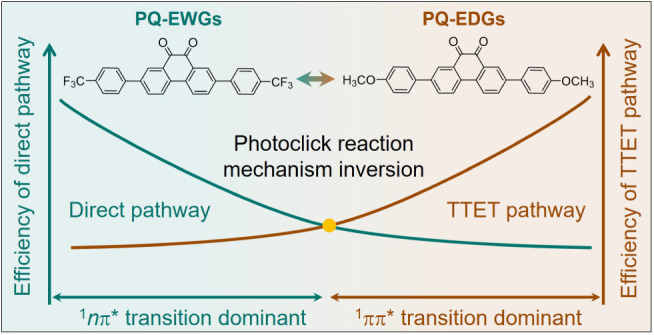

## Introduction

Photochemical activation^1^ in click chemistry^2–5^ has led to the development of a novel class of light-induced transformations known as photoclick chemistry.^6–8^ Unlike conventional click reactions, which often lack precise spatial and temporal control,^9^ photochemical reactions enable highly controlled activation, allowing the reaction to occur with high precision while triggered noninvasively on demand.^1^ The photoinduced cycloaddition of a tetrazole and an alkene, first reported by Lin and coworkers,^10^ marked the inception of a decade of remarkable advancements in this field. Since then, various photoclick reactions have been explored.^11–17^ The unique advantages of photoclick chemistry—including the noninvasive use of light, spatiotemporal control, high efficiency, and compatibility with biological systems—make it particularly well-suited for a wide range of applications. In addition, photoclick chemistry has recently emerged as a promising methodology for surface functionalization,^18–20^ polymer conjugation,^21^ photo-cross-linking,^22,23^ bioimaging,^15,24^ and protein labeling.^17,25,26^

A critical limitation of conventional photoclick reactions typically pertains to their reliance on ultraviolet (UV, *λ*<380 nm) irradiation,^7,8,27^ as exemplified by photoinduced tetrazole–alkene cycloadditions,^10^ UV-initiated thiol–ene/yne reactions,^11,28^ azide–alkyne cycloadditions,^12^ sydnone–alkene/alkyne ligations, azirine–alkene cycloadditions,^29^ and light-activated oxime ligations.^30^ However, visible (vis, *λ* = 380–700 nm) and near-infrared (NIR, *λ* = 700–2500 nm) light-induced photoclick systems are highly warranted because of their deeper tissue penetration,^8^ reduced phototoxicity,^31^ and minimized background interference in complex organic matrices (*e.g.,* polymers, tissue).^32–36^ These attributes are particularly advantageous for noninvasive subsurface imaging and *in vivo* applications, where the use of UV light is associated with risks like inducing cellular damage or apoptosis.^37,38^

Current strategies to redshift photoclick reactivity^8^ focus on the π-extension of chromophores to narrow the energy gap between the highest occupied molecular orbital and the lowest unoccupied molecular orbital. However, this often compromises reaction efficiency and aqueous solubility, thereby hindering potential biological applications.^39–41^ Alternative approaches, such as multiphoton absorption^42^ or upconversion mechanisms,^43,44^ face practical limitations: multiphoton processes demand high-intensity pulsed lasers with low quantum yields, while upconversion systems suffer from inefficient luminescence and reliance on prolonged high-power irradiation.

A pivotal yet underexplored dimension in photoclick chemistry is the role of excited-state evolution processes.^1,7,8^ For the conventional direct pathway, UV excitation of phenanthrenequinones (**PQ**s) populates the S_2_ (^1^ππ*) state, which undergoes rapid internal conversion to the S_1_ (^1^ *n*π*) singlet state. Efficient intersystem crossing (ISC) then generates the reactive T_1_ (^3^ππ*) triplet state, enabling [4 + 2] photocycloaddition with alkenes (Scheme 1a, left).^15,16,45–48^ On the other hand, triplet–triplet energy transfer (TTET)^49–52^ might offer a versatile alternative strategy to perform photoclick reactions, bypassing wavelength constraints by decoupling excitation and reactivity. Here, a visible-light-absorbing photosensitizer (*e.g.,* Bodipy derivatives) transfers energy to an acceptor (*e.g.,* **PQ**), circumventing the need for direct chromophore modification. This approach has enabled all-visible-light control of photoswitches (*e.g.,* diarylethenes,^53–55^ azobenzenes)^56–58^ and molecular motors.^59,60^ Our group recently pioneered the first long-wavelength visible light-induced TTET photoclick system by doping **PQ**s with Bodipy sensitizers (Scheme 1a, right).^46,47^ Green/yellow light (530–590 nm) efficiently triggered the [4 + 2] photocycloaddition reaction between **PQ**s and electron-rich alkenes (**ERA**s), showcasing high reactivity without any structural modifications.

**Scheme 1:**
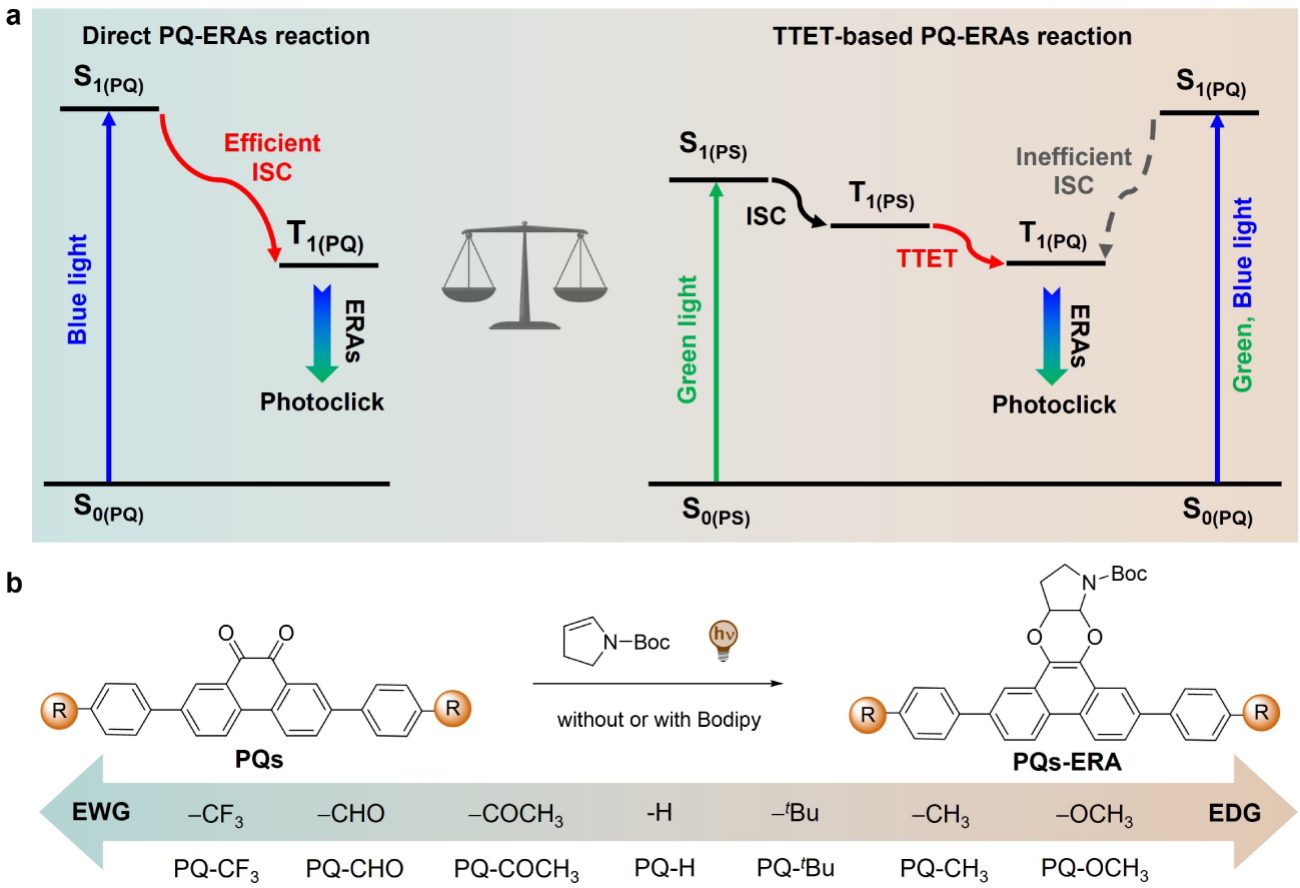
(a) Illustration of the **PQ–ERA** Photoclick Reaction Mechanisms; Left Panel: the Direct Pathway through a Direct and Efficient Intersystem-Crossing (ISC) Process; Right Panel: the TTET-Based Photoclick Pathway Promoted by a Triplet Photosensitizer (PS); (b) the Rational Design of **PQ** Derivatives with Different Substitutions from Electron-Withdrawing Groups (EWGs) to Electron-Donating Groups (EDGs)

Despite these advances, critical gaps in our understanding of excited-state processes still hinder the rational design of molecules with favorable photoclick properties. First, there is the mechanistic ambiguity: the transition from general photocycloaddition to the TTET pathway lacks systematic studies. As a result, key questions, such as the exact point of energy transfer, the role of sensitizer-acceptor orbital alignment, and solvent/subsidiary group effects on TTET efficiency, remain unanswered so far. Second, there is a scarcity of appropriate reaction partners; as yet, only a few TTET-induced photoclick systems have been reported, limiting the possibility to come up with universal design principles. For instance, the interplay between EDG-induced electronic energy modulation (*e.g.,* T_1_ energy lowering) and TTET kinetics requires a broader structural basis.

Facing these challenges and encouraged by the vast range of potential applications, we designed and synthesized a series of 2,2’-substituted **PQ** derivatives (Scheme 1b), incorporating substituents ranging from electron-withdrawing groups (EWGs) to EDGs, and conducted comprehensive investigations into their photoclick-reaction mechanism. Not unexpectedly, we show that introducing electronic groups red-shifts the absorption wavelength to the visible-light range of 440–600 nm due to an increased push–pull effect. Interestingly, with the increased electron-donating ability of the substituents, the photoclick efficiency via the direct pathway decreases while that of the TTET-mediated pathway is enhanced. Theoretical calculations and nanosecond transient absorption studies show that these observations can be well explained by an inversion of the ^1^ *n*π* and ^1^ππ* states, as a result of which ISC yields and the reaction mechanism are strongly modulated. It was also found that the reaction mechanism could be regulated by modifying the polarity of the solvent, with low-polarity solvents favoring the direct pathway, whereas high-polarity solvents promote the TTET pathway. These findings provide key insights into the mechanistic understanding of these reactions and a robust strategy for tailoring the reaction mechanisms of **PQ–ERA** photoclick reactions.

## Results and Discussion

### Synthesis and Photophysical Evaluation of **PQ** Derivatives

To establish a clear structure–property relationship and mechanism scheme, we investigated the effects of substituents on the properties of **PQ** (Scheme 1b) by using a variety of functional groups. For this purpose, we extended the **PQ** core with substituted phenyl moieties based on the method reported in our previous study.^16^ Starting with 2,7-diiodinephenanthrene-9,10-dione and commercially available phenylboronic acids, the Suzuki–Miyaura cross-coupling reaction^61^ allows for straightforward synthesis. Unlike direct **PQ** core modifications, palladium-mediated coupling tolerates various substrates without altering the protocol, yielding a **PQ** library with satisfying product formation employing Pd­(PPh_3_)_4_ as the catalyst (35%–55% yields; for details, see Scheme S2 and Section S2).

Next, these target compounds were characterized in terms of their electronic properties. Analysis of the UV–vis absorption spectra of phenyl-extended **PQ**s revealed that the lowest-energy absorption band (_max_) is bathochromically shifted compared to the unsubstituted **PQ** (*λ*_max_ = 410 nm), resulting in wavelengths longer than 440 nm (Figure S40).^16,45,48^ This band is typically used to excite **PQ** derivatives to trigger the **PQ–ERA** photoclick reaction. Moreover, the modification of the *para*-position of the phenyl group from −CF_3_ to −OCH_3_ induces a significant redshift from 440 to 520 nm, indicative of the change in electronic structure and the push–pull effect induced by the different substituents (Figure S40).

### Photoclick-Reaction Kinetics of **PQ**s with **ERA** via Direct Pathway

The performance of the newly synthesized **PQ** derivatives in a [4 + 2] **PQ–ERA** photocycloaddition reaction via the direct pathway was first evaluated. The reaction partner, *N*-boc-2,3-dihydro-1*H*-pyrrole (**PY**, 10 equiv), was chosen as the **ERA** due to its high reactivity found in our previous studies.^16,45,48^ Solutions of each **PQ** derivative and **PY** in MeCN (50 μM/500 μM, under N_2_ atmosphere) were directly irradiated (without the addition of Bodipy as a photosensitizer) under 440, 455, or 520 nm LEDs selected according to the respective _max_ values of **PQ**s. The reaction progress was continuously monitored via UV–vis spectroscopy (Figure 1a–c; see Section S4.2 and Figures S41–S47 for full details).

**Figure 1:**
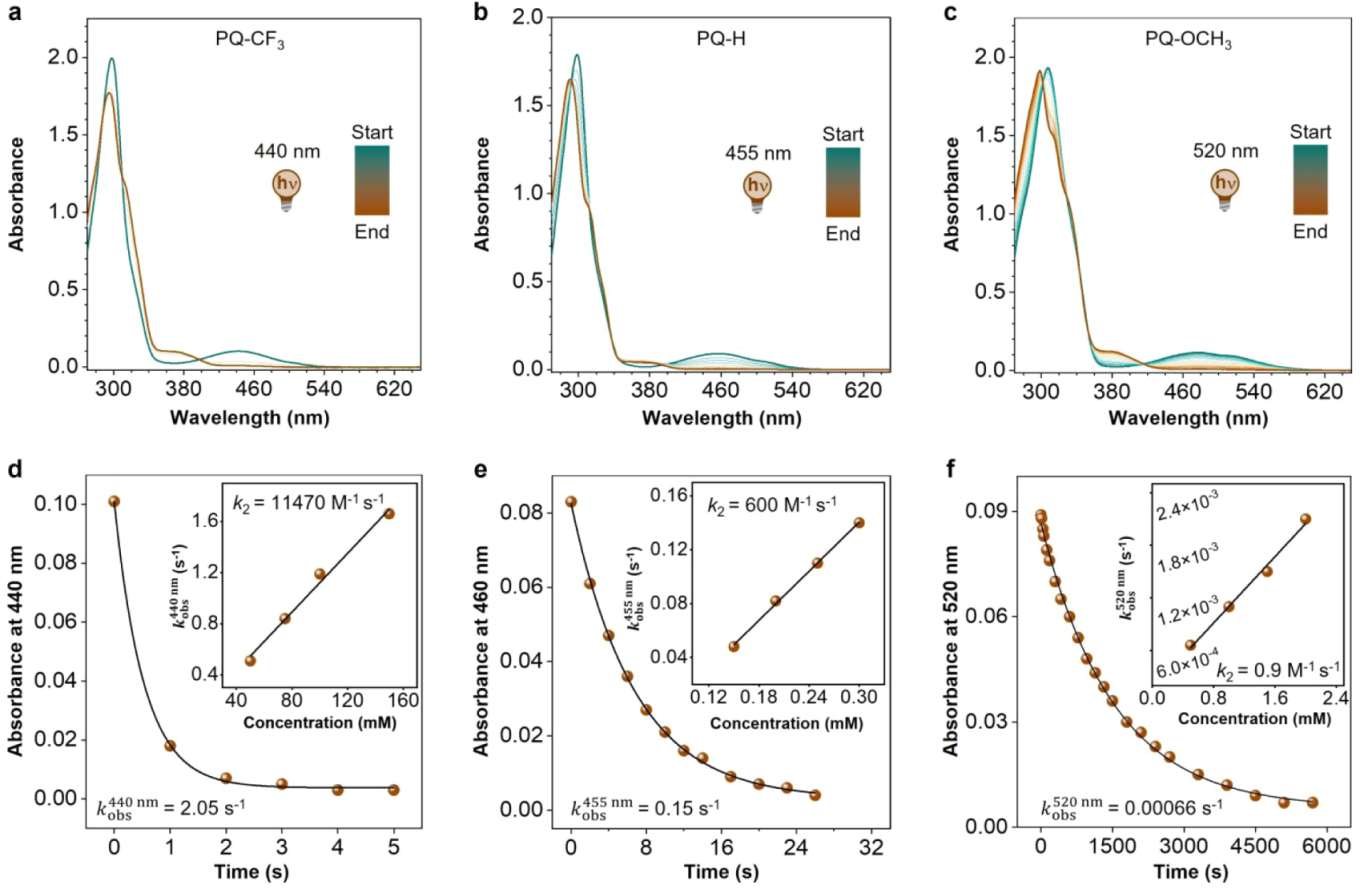
Photoinduced [4 + 2] cycloaddition of **PQ**s with **PY**. (a–c) Time-resolved UV–vis absorption spectra for the photocycloaddition of (a) **PQ–CF**_**3**_, (b) **PQ–H**, and (c) **PQ–OCH**_**3**_ to **PY**, respectively. Samples were irradiated by a 440 nm (**PQ–CF**_**3**_), 455 nm (**PQ–H**), and 520 nm (**PQ–OCH**_**3**_) LED under N_2_ atmosphere at 20 °C in MeCN, spectra measurement interval: 1 s; concentration: 50 μM **PQ**s and 500 μM **PY**. (d–f) Kinetic traces (*k*_obs_) for the photocycloaddition of (d) **PQ–CF**_**3**_, (e) **PQ–H**, and (f) **PQ–OCH**_**3**_ to **PY**, respectively. Inset: the second-order rate constant (*k*_2_) for photocycloaddition determined by the slope of the fitted line.

Similar to a previous study, a pseudo-first-order reaction was observed for all **PQ–ERA** photoclick reactions with a pronounced reactivity trend: from **PQ** with EWGs (e.g., −CF_3_, −CHO, and −COCH_3_) to those with EDGs (e.g., −^*t*^Bu (*tert*-butyl), −CH_3_, and −OCH_3_), the reaction rate gradually decreased by several orders of magnitude. Specifically, **PQ–CF**_**3**_, bearing the strongest EWG, exhibited the highest reaction rate constant (*k*_obs_) of 2.05 s^–1^ (Figure 1d). With a comparatively weaker EWG, **PQ–CHO** and **PQ–COCH**_**3**_ showed decreased *k*_obs_ values of 1.45 s^– 1^ and 1.25 s^–1^ (Figures S42 and S43), respectively, while for the model compound **PQ–H** without any substitution a further reduced value of 0.15 s^–1^ was found (Figure 1e). Interestingly, EDG substituents led to a marked decrease in *k*_obs_. With the increased electron-donating ability of EDGs, **PQ–** ^***t***^ **Bu**, **PQ–CH**_**3**_, and **PQ–OCH**_**3**_ exhibited gradually decreasing *k*_obs_ values of 0.026 s^–1^, 0.016 s^–1^, and 0.00066 s^–1^, respectively (Figures 1f, S45, and S46).

To further investigate the differences in reaction rates induced by these substitutions, we quantitatively determined the second-order reaction rate constant (*k*_2_) of the **PQ–ERA** photoclick reaction—another critical kinetic parameter governing biomolecular conjugation efficiency (Figure 1d–f inset; for detailed information, Section S4.2 and Figures S41–S47). Notably, the *k*_2_ values exhibited a pronounced substitution-related effect that paralleled the observed trend in *k*_obs_, with

EWGs on the **PQ** scaffold significantly enhanced the direct-pathway reactivity compared to EDGs. For instance, under standardized reaction conditions, the **PQ–CF**_**3**_ **_PY** system demonstrated remarkable kinetic performance with *k*_2_ = 11,470 M^–1^ s^–1^ (Figure 1d). This represents a 19-fold enhancement over the **PQ–H_PY** reference system (600 M^–1^ s^–1^, Figure 1e) and a quite impressive 12,744-fold acceleration relative to the EDG-containing **PQ–OCH**_**3**_ **_PY** analog (0.9 M^–1^ s^–1^, Figure 1f). Such unprecedented rate enhancements enable quantitative photoclick conjugation within seconds, establishing this as the fastest-reported **PQ–ERA** system to date.^15–17,45,48^ To assess the biocompatibility of the **PQ–ERA** reaction, the **PQs_PY** photoclick reactions were performed in PBS buffer/MeCN mixtures (0–50% PBS buffer in MeCN, cf. Figures S48–S50). We found that the photoclick reaction still proceeds efficiently in a 1:1 PBS buffer/MeCN mixture, exhibiting rates of *k*_obs_ as 0.012 s^–1^, 0.002 s^–1^ and 0.026 s^–1^, respectively, upon 440, 455, and 520 nm light irradiation. While the limited solubility of the **PQ** derivatives did not allow us to perform the experiments in the absence of organic solvent additives, we could prove that the reaction proceeds efficiently in the presence of biocompatible media.

### Theoretical Calculations of **PQ**s

To gain further fundamental understanding of this new phenomenon, we performed (time-dependent) density functional theory ((TD-)­DFT) calculations at the MN15/def2-TZVP/PCM level, which have previously been used successfully to evaluate various photochemical processes.^62–67^ The calculated vertical energy levels based on the optimized ground-state geometries fit well with their absorption spectra, showing a gradually red-shifted wavelength of λ_max_ from the EWG-substituted to EDG-substituted **PQ**s (Table S1, Figures S40 and S70). In addition, since the direct photoclick reaction of **PQ**s involves ISC, the diabatic energy and electronic features of both the lowest singlet (S_1_) and triplet (T_1_) states were further evaluated based on the optimized excited-state geometries (Figure 2 and Table S2). Interestingly, an energy-level inversion was observed that the S_1_ state of EWG-substituted **PQ**s was dominated by ^1^nπ* transition while that of **PQ–H** and other EDG-substituted **PQs** was dominated by ^1^ππ* transition. Nevertheless, the T_1_ state of all compounds exhibits the transition typical of ^3^ππ*. The different electronic features of these two types of **PQ**s could also be visualized by hole–electron analysis (Figures 2b,c, S70, and S71). According to El-Sayed’s rules,^68^ the efficiency of ISC between singlet and triplet states is governed by their electronic features: a large difference (^1^ *n*π* → ^3^ππ* or ^1^ππ* → ^3^ *n*π*) promotes ISC, while the same feature (^1^ *n*π* → ^3^ *n*π* or ^1^ππ* → ^3^ππ*) would diminish ISC. Based on the above results, the different reaction rates of the general photoclick pathway could be reasonably explained by the reversed energy levels and ISC efficiency. For **PQ–CF**_**3**_, after photoexcitation, the S_1_ state involving a ^1^ *n*π* transition and T_1_ based on the ^1^ππ* ensures their efficient ISC and accumulation of triplet excitons for the following photocycloaddition. With weaker electron acceptors, the singlet states with ^1^ *n*π* and ^1^ππ* get close to each other and decrease the ^1^ *n*π* character of S_1_, resulting in a slightly declined ISC and reaction rate. In contrast, the dominant S_1_ state with ^1^ππ* character and the gradually increased energy gap between the two states based on ^1^ *n*π* and ^1^ππ* features result in inefficient ISC and decreased accumulation of triplet excitons, finally suppressing the direct photoclick reactivity. This structure–kinetic relationship established electronic modulation of excited-state dynamics as a critical design principle for **PQ–ERA** photoclick systems.

**Figure 2:**
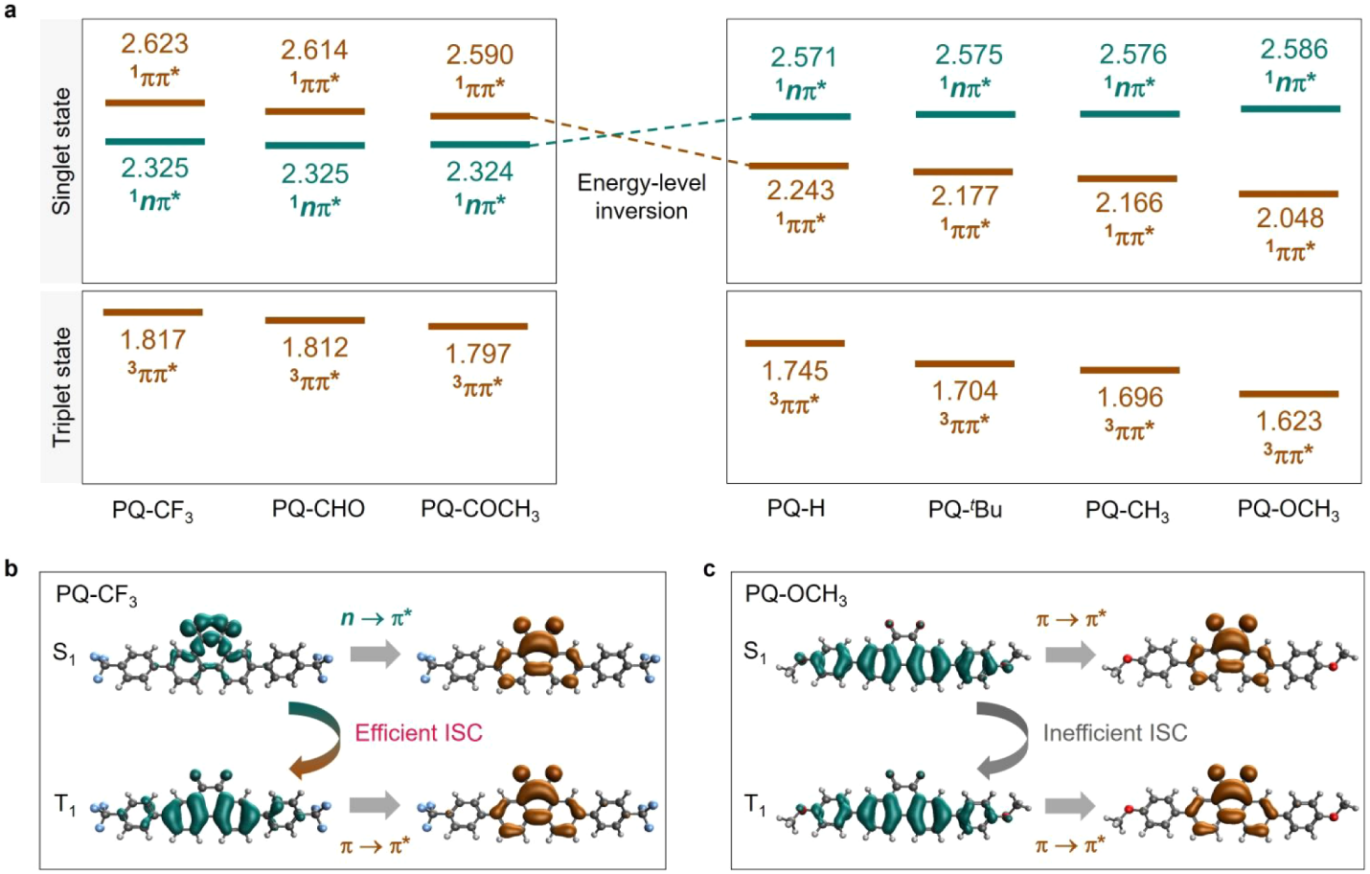
Theoretical calculation of **PQ**s. (a) Adiabatic energies (unit in eV) of the singlet and triplet states for **PQ** derivatives based on the optimized excited geometries, computed at the MN15/def2-TZVP level with PCM (MeCN) solvation. **PQ–CF_3_**, **PQ–CHO**, and **PQ–COCH_3_** retain the ^1^ *n*π^*^ dominated the lowest singlet state, while **PQ–H**, **PQ–*^t^* Bu**, **PQ–CH_3_**, and **PQ–OCH_3_** exhibit the lowest singlet state with ^1^ππ^*^ character, indicating the substituent-induced energy-level inversion. (b,c) Hole-electron analysis of (b) **PQ–CF_3_** and (c) **PQ–OCH_3_**. The hole and electron parts are shown in green and brown color, respectively. The different electronic characters of the lowest singlet and triplet state promote efficient S_1_ → T_1_ ISC while the same nature of these diminishes the ISC efficiency.

### Transient Absorption Spectroscopy Analysis of **PQ**s

To confirm unambiguously the hypothesis that the differences in reaction rates are due to differences in the efficiency with which the lowest triplet state is populated and its lifetime, we have performed nanosecond transient absorption spectroscopy. Figure 3 shows for this purpose decay-associated difference spectra (DADS) obtained from the global analysis of transient absorption spectra of **PQ–CF**_**3**_, **PQ–H**, and **PQ–OCH**_**3**_ in acetonitrile with the associated time-resolved absorption spectra being reported in Figures S62, S65, and S68. Such global analyses show for **PQ**–**CF**_**3**_ two components that we associate, on the basis of our previous studies, with the triplet state (*t* = 7.9 μs) and the ketyl radical (*t* = 92.0 μs).^48^ For **PQ–H**, two components are also observed, of which the (*t* = 2.2 μs) component is attributed on the basis of its spectrum with the decay of the triplet state, while for **PQ**–**OCH**_**3**_ only a single component is observed. Ideally, comparative transient absorption experiments are to be performed on solutions with the same optical density and using the same excitation pulse energies, but due to the limited solubility of **PQ**–**H** and **PQ**–**OCH**_**3**_ different optical densities and pulse energies needed to be used. Correcting for these differences one finds normalized triplet quantum yield ratios for **PQ**–**CF**_**3**_:**PQ**–**H**:**PQ**–**OCH**_**3**_ of roughly 1:0.3:0.035 in excellent agreement with the predicted inversion of the ^1^ππ* and ^1^ *n*π* states. One point meriting further attention is that such an inversion already occurs for **PQ**–**H**, which might lead one to expect much lower triplet quantum yields. However, the energy difference between these two states (0.328 eV) is such that at room temperature, the ^1^ *n*π* state is still an accessible pathway along which ISC can occur.

**Figure 3:**
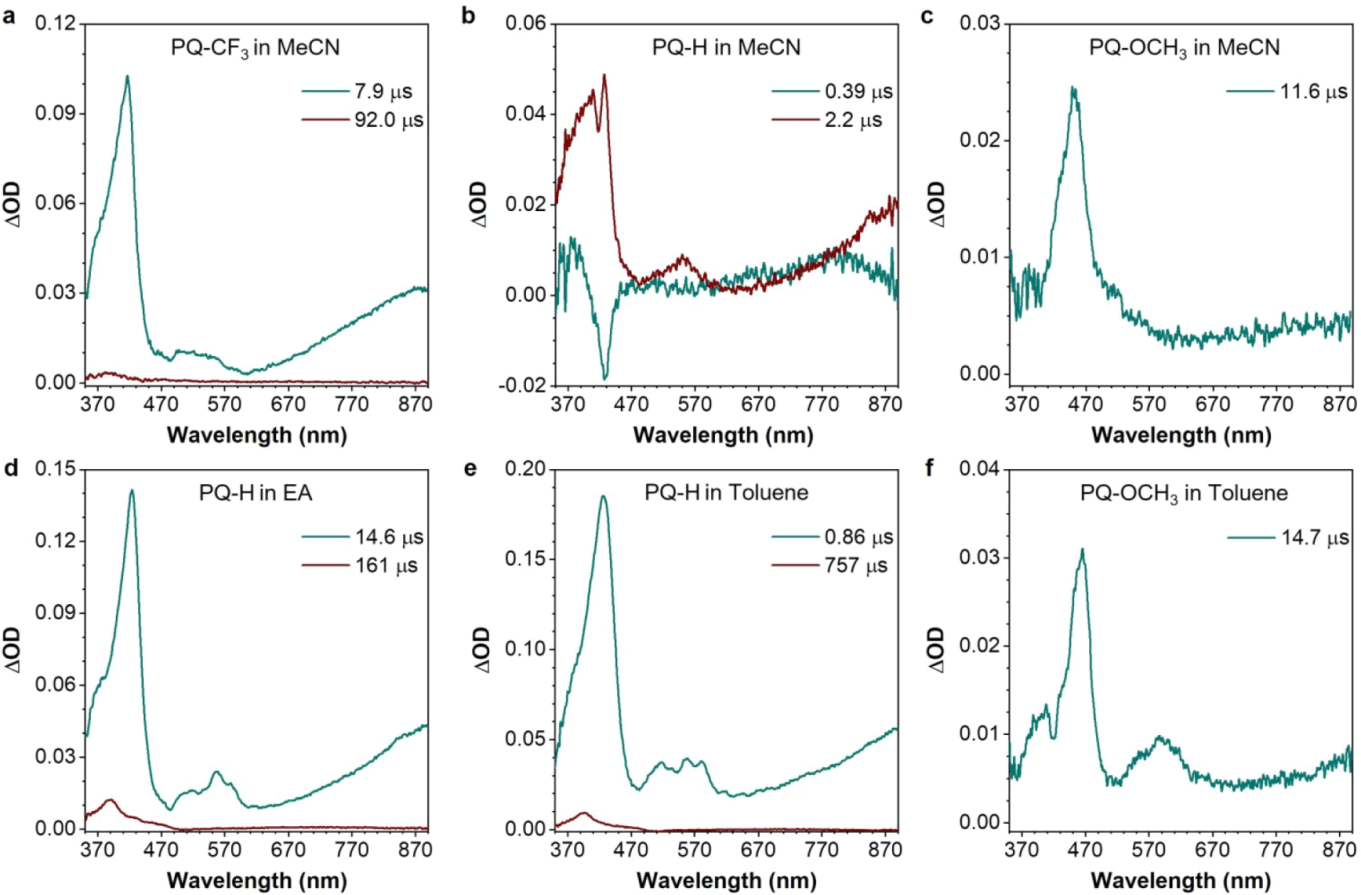
Decay-associated difference spectra (DADS) and decay times obtained from the global analysis of transient absorption spectra of deaerated solutions of (a) **PQ**–**CF**_**3**_, (b) **PQ**–**H**, and (c) **PQ**–**OCH**_**3**_ in acetonitrile with optical densities of 1.4, 0.5, and 0.7, respectively, using pulse excitation energies of 0.1, 1.0, and 3.5 mJ, respectively. Panels (d–f) display such spectra and decay times for deaerated solutions of **PQ–H** in ethyl acetate, toluene with optical densities of 1.0 and 1.6, and **PQ–OCH**_**3**_ in toluene, respectively, using pulse excitation energies of 0.1 mJ.

### Photoclick-Reaction Kinetics of **PQ**s with **ERA** via TTET Pathway

The above theoretical and spectroscopic results indicate that the **EDG**s functionalized **PQ** derivatives do not perform well in the direct **PQ–ERA** reaction under LED irradiation. However, according to our previous studies,^46,47^ their photoclick activity might be reactivated with the assistance of suitable photosensitizers using the TTET pathway. To verify our hypothesis, a photosensitizer (1,3,5,7,8-pentamethyl-2,6-diiodo-Bodipy (**DiIbodipy**)) with high ISC efficiency and a high molar extinction coefficient was selected as a candidate to establish the TTET-based **PQ–ERA**s photoclick reaction.^46^ Initial investigations focused on evaluating the TTET-induced photoclick performance of **PQ** derivatives modified with an electron-donating group (including **PQ–H**). Photoirradiation (520 nm LED, _max_ = 525 nm for **DiIbodipy**; *ε* = 3.3 × 10^4^ M^–1^ cm^–1^) of degassed acetonitrile solutions containing **PQ**s (50 μM, 1 equiv), **PY** (500 μM, 10 equiv), and **DiIbodipy** (50 μM, 1 equiv) revealed pronounced TTET activation. UV–vis kinetic monitoring (Figure 4a–c; for detailed information, see Section S4.2 and Figures S51–S57) demonstrated significant *k*_obs_ enhancements in TTET systems versus **DiIbodipy**-free controls. The methoxy-substituted derivative **PQ–OCH**_**3**_ exhibited optimal performance with *k*_obs_ = 0.46 s^–1^ (Figures 4f and 5), representing a 700-fold acceleration over its non-TTET counterpart (Figure 1f). In contrast, **PQ**s functionalized with an electron-withdrawing group (*e.g.,* −CF_3_, −CHO, −COCH_3_) displayed opposite behavior (Figures 5 and S51–S53).

**Figure 4:**
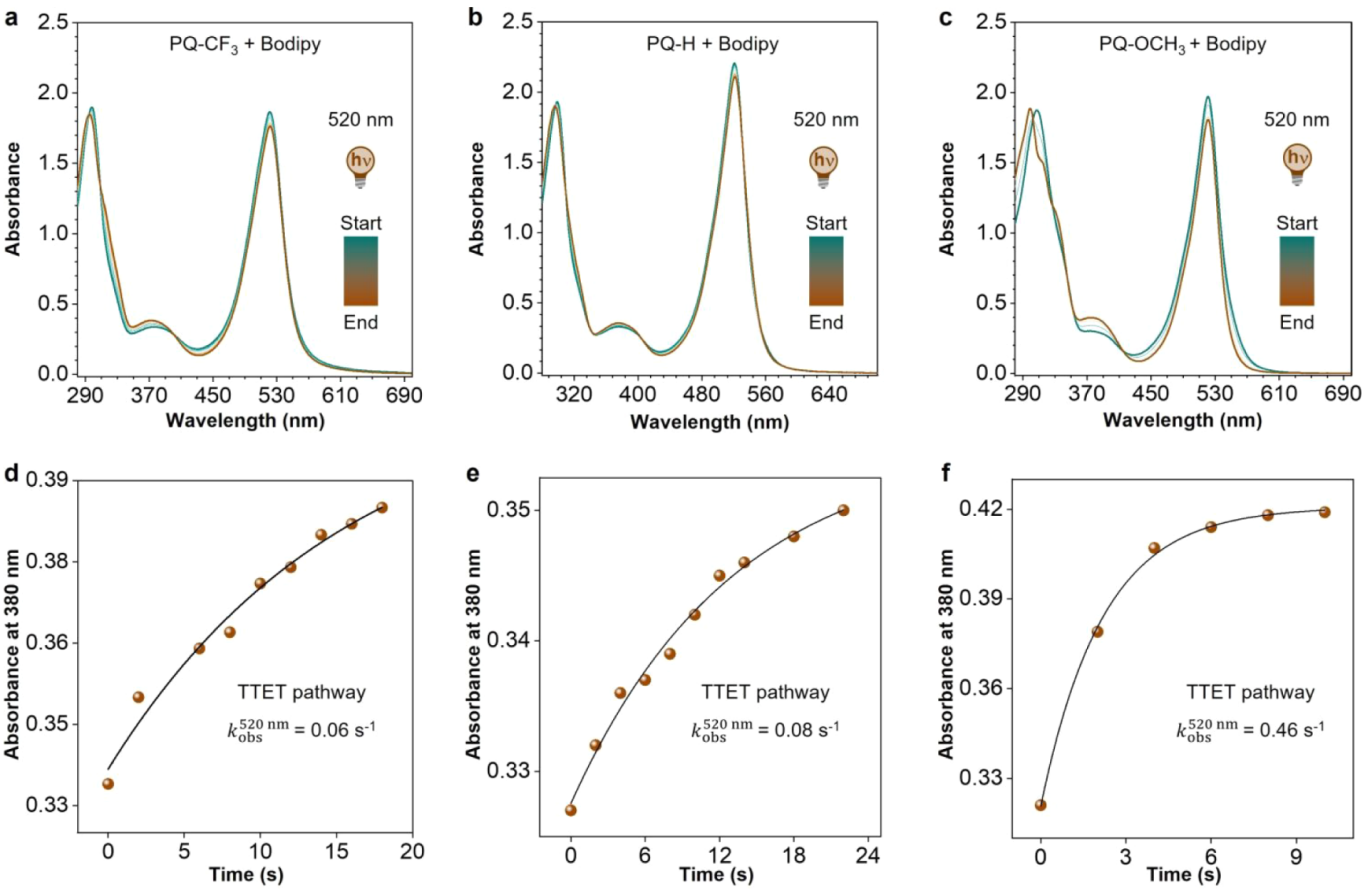
Kinetic analysis of TTET-initiated **PQ–ERA** photocycloadditions. (a–c) Time-resolved UV–vis absorption spectra of the (a) **PQ–CF**_**3**_, (b) **PQ–H**, and (c) **PQ–OCH**_**3**_ to **PY** photoclick reaction, respectively. A solution containing 50 μM **PQ**s, 50 μM photosensitizer (**DiIbodipy**) and 500 μM **PY** in 2.5 mL of MeCN (N_2_ atmosphere) was irradiated under a 520 nm LED at 20 °C. (d–f) Kinetic traces for the (d) **PQ–CF**_**3**_, (e) **PQ–H**, and (f) **PQ–OCH**_**3**_ to **PY** cycloaddition reactions, respectively. **PQs-PY** formation has been fitted to $y={(y}_{0}-a)e^{k_{\mathrm{obs}}^{520\mathrm{nm}}\times t}$ to give $k_{\mathrm{obs}}^{520\mathrm{nm}}$ .

**Figure 5:**
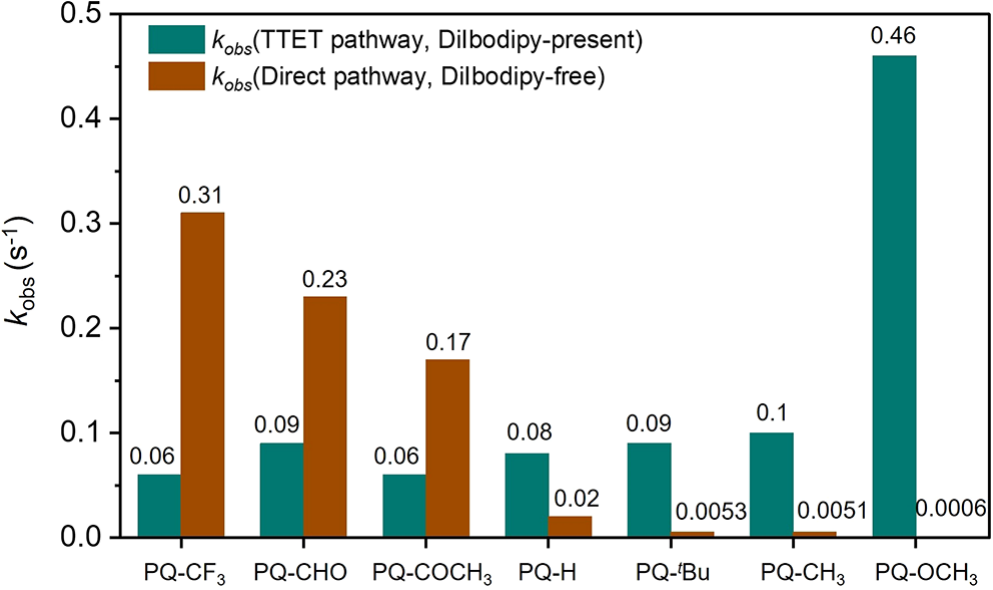
Comparative kinetic analysis of the **PQ–ERA** photocycloaddition reaction rate constant *k*_obs_ for the TTET pathway (DiIbodipy-present) versus the direct pathway (DiIbodipy-free) with all samples subjected to the same LED irradiation at 520 nm.

To further exclude the influence of the excitation wavelength, all samples were subjected to the same LED of 520 nm to compare their reaction kinetics. As shown in Figure 5, while enabling detectable photoclick reactivity (*k*_obs_ in the range of 0.06–0.09 s^–1^) under TTET conditions, their kinetics were markedly suppressed compared to **DiIbodipy**-free systems (*e.g.,* −CF_3_: 0.31 s^–1^ → 0.06 s^–1^; −CHO: 0.23 s^–1^ → 0.09 s^–1^; −COCH_3_: 0.17 s^–1^ → 0.06 s^–1^). This dichotomy suggests a dominance of the direct pathway for **PQ–EWG**s, contrasting sharply with the **PQ–EDG**s (Figures 5 and S54–S57). Notably, the mechanistic inversion observed at **PQ–H** shifting from a dominant direct pathway to TTET-dictated kinetics provides crucial evidence for a substituent-dependent modulation of excited-state evolution in **PQ–ERA** systems.

To elucidate the regulatory role of the photosensitizer concentration on TTET-induced photoclick reaction kinetics and to define the structure–activity relationship underlying its concentration-dependent effects on excited-state energy transfer efficiency and intermolecular interactions, we systematically investigated the impact of **DiIbodipy** loading (0.005–0.5 equiv) on the reaction kinetics of the **PQ–OCH**_**3**_ **_PY** system. To our delight, a fast photoclick reaction was also observed even with only 0.005 equiv **DiIbodipy** addition under green (520 nm) light irradiation (*k*_obs_= 0.081 s^–1^; for detailed information, see Section S4.2 and Figure S57). Interestingly, *k*_obs_ exhibited a nonmonotonic dependence on the **DiIbodipy** concentration, deviating from conventional positive correlation trends. Kinetic profiling reveals an optimal *k*_obs_ value of 0.92 s^–1^ at 0.25 equiv of **DiIbodipy** loading, which exhibited the fastest TTET-induced **PQ–ERA** photoclick within all reported studies; note that full conversion is reached within 4 s,^47,48^ beyond which the reaction kinetics become progressively attenuated. This counterintuitive concentration–response relationship is attributed to the concentration-dependent self-quenching of **DiIbodipy** triplets. At elevated concentrations, enhanced intermolecular interactions facilitate competitive triplet–triplet annihilation (TTA)^49^ pathways, effectively diminishing the critical TTET efficiency for catalytic turnover. Such behavior aligns with established concentration quenching mechanisms in molecular photosensitizer systems, for which an increased collision frequency between excited-state species promotes nonproductive energy dissipation via Dexter-type electronic coupling (for detailed information, see Section S4.2 and Figure S61).^49^

The observed substituent-dependent inversion of the ^1^ *n*π* and ^1^ππ* states led to the question whether it would also be possible to modulate the reaction efficiency of the direct pathway of the **PQ–ERA** photocycloaddition using a judicious choice of solvent, since it is well-known that the excitation energies of ^1^ *n*π* and ^1^ππ* depend in opposite ways on the polarity of the solvent.^69^ Therefore, three **PQ**s bearing distinct substituents (e.g., **PQ–CF**_**3**_, **PQ–H**, and **PQ–OCH**_**3**_) were employed as model compounds for the systematic investigation of solvent dependency. The kinetic behavior of these **PQ**s in toluene (nonpolar), ethyl acetate (EA, moderately polar), and MeCN (strongly polar) was monitored in real time (Figures 6 and S58–S60). Notably, the EWG-modified **PQ** (**PQ–CF**_**3**_) exhibited a remarkably high *k*_obs_ (1.44–2.05 s^–1^) in all solvents with different polarities, while the EDG-modified **PQ** (**PQ–OCH**_**3**_) displayed a significantly reduced reaction rate with *k*_obs_ lower than 0.13 s^–1^ (Figure 6c). These results suggest that the modifications of the ^1^ *n*π* and ^1^ππ* energy levels by the polarity of the solvent do not influence their reaction pathways. In sharp contrast, a huge change in *k*_obs_ was observed for **PQ–H** when different solvents were applied. The values determined for *k*_obs_ using toluene and EA (1.48 s^–1^ and 1.45 s^–1^, respectively) are comparable to or slightly lower than those of **PQ–CF**_**3**_, but in MeCN this rate is drastically slowed down by an order of magnitude to only 0.15 s^–1^, suggesting a similar energy-level inversion and, as a consequence, an inefficient ISC similar to that observed by changing substituents (as shown in Figures 1 and 2).

**Figure 6:**
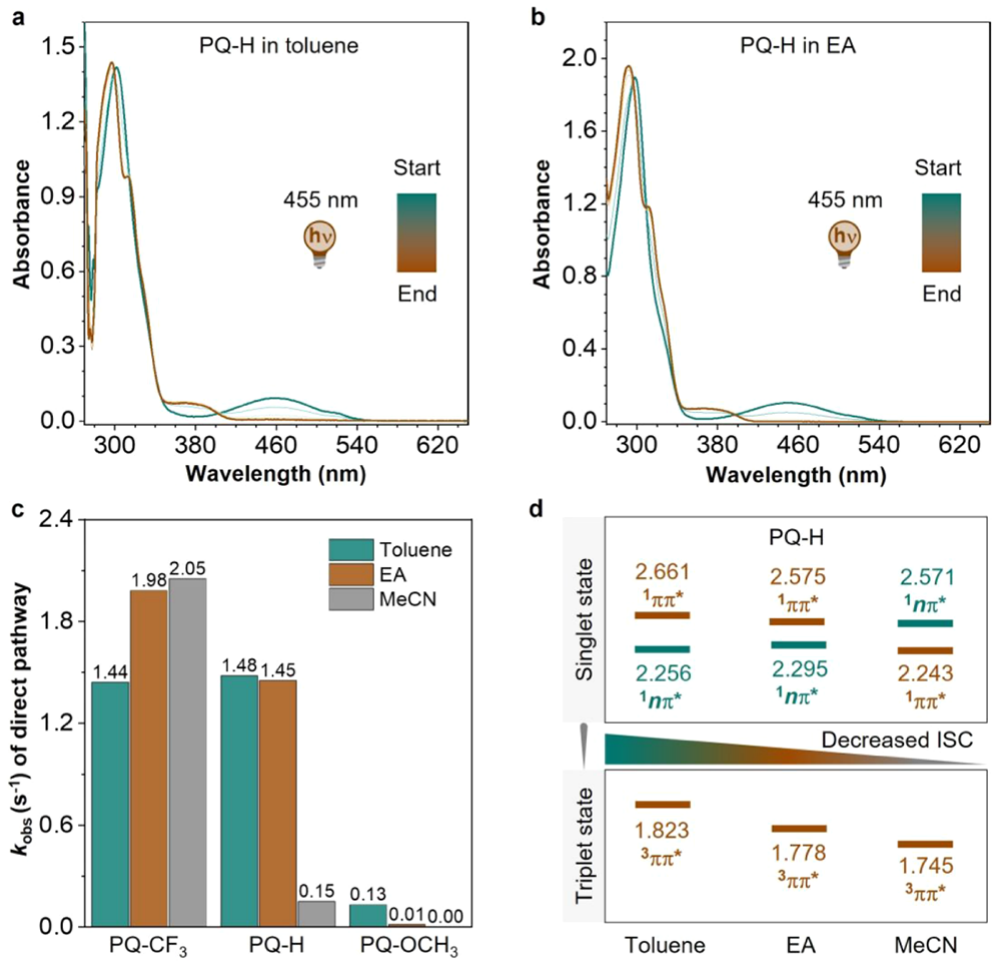
Solvent effect on the direct pathway of the **PQ–ERA** photoclick reaction. (a,b) Time-resolved UV–vis absorption spectra of the **PQ–H** photocycloaddition reaction to **PY** under 455 nm LED irradiation (20 °C) in (a) toluene and (b) ethyl acetate (EA), respectively. (c) Solvent-dependent *k*_obs_ for **PQ–CF**_**3**_, **PQ–H**, and **PQ–OCH**_**3**_ in toluene, EA, and MeCN with different polarities. (d) Adiabatic energies (eV) of the singlet and triplet states for **PQ**–**H** in different solvents based on the optimized excited geometries computed at the MN15/def2-TZVP level with the PCM solvation model.

Theoretical calculations fully support such a hypothesis. As depicted in Figure 6d, increasing the polarity causes a red shift of the excitation energy of the ^1^ππ* state, while the ^1^ *n*π* state of **PQ–H** is blue-shifted, resulting in the energy-level inversion. Such an inversion significantly diminishes the ISC efficiency and limits the photoclick reaction, which fits well with the experimental results. For **PQ**–**CF**_**3**_, on the other hand, the calculations show that the ^1^ *n*π* state always remains the lowest excited singlet state, in line with the experimentally observed absence of a dependence of the reaction rate on the solvent polarity. Transient absorption spectroscopy allows us also here to unambiguously confirm the insight obtained from the calculations as can be concluded from panels (b), (d), (e), and (c), (f) in Figure 3 that show DADS obtained from the global analysis of transient absorption spectra of **PQ–H** in acetonitrile, ethyl acetate, toluene, and **PQ–OCH**_**3**_ in ethyl acetate and toluene, respectively (associated time-resolved absorption spectra shown in Section S4.2 and Figures S62–S69). Similar to our previous discussion (vide supra), we associate the black and red DADS in ethyl acetate and toluene with the decay of the triplet state of **PQ–H** and its photogenerated ketyl radical, respectively. Inspection of these DADS shows a comparable triplet quantum yield in ethyl acetate and toluene, but a significantly reduced one in acetonitrile. We therefore conclude that solvent polarity is another means to modulate the reaction efficiency of **PQ**s.

Based on the above data and analysis, a general mechanistic scheme for the **PQ–ERA** photoclick reaction can be drawn (Figure 7). For **PQ**s with EWG groups and **PQ**s in low-polarity solvents, the lowest excited singlet state is a ^1^ *n*π* state, which exhibits strong spin–orbit coupling with the T_1_^3^ππ* state, resulting in efficient ISC and high triplet quantum yields. As a result, the **PQ–ERA** photoclick reaction can occur through a direct pathway. On the contrary, for **PQs** with EDG and **PQ**s in high-polarity solvents, both S_1_ and T_1_ are ππ* states for which ISC is much less efficient. Thus, suitable photosensitizers are required to promote the photoclick reaction via the TTET-mediated pathway to populate the triplet states of these **PQ**s. Based on this electron-effect and solvent-polarity-induced mechanism variation, molecule-engineering strategies and reaction conditions might be further regulated regarding different environments to promote potential applications of the **PQ–ERA** photoclick reaction in complex and biological systems.

**Figure 7:**
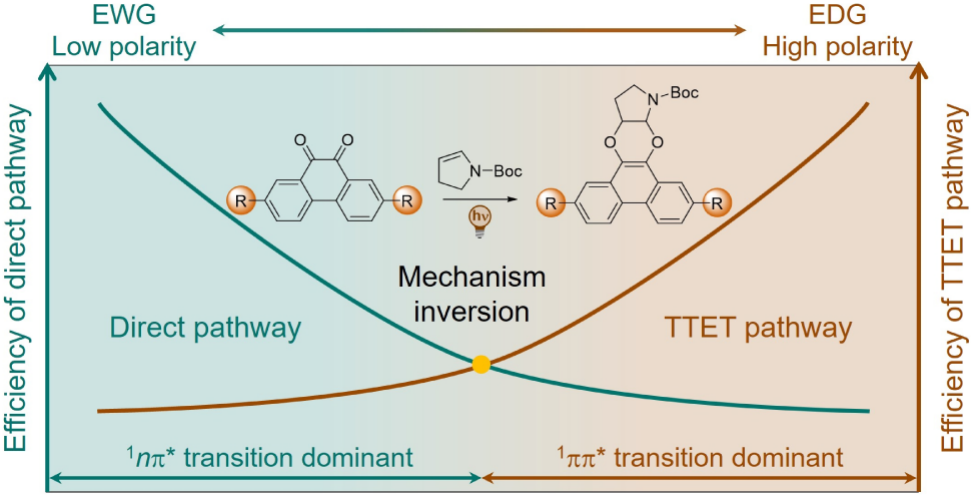
Mechanism inversion scheme between the direct pathway and TTET-mediated pathway of the **PQ–ERA** photoclick reaction under the influence of electronic and solvent polarity effects.

## Conclusion

In summary, we have demonstrated for the first time that the reactivity and mechanism of **PQ–ERA** photoclick reactions can be fully modulated by strategic substituent tuning on 2,2’-substituted **PQ** derivatives and by varying the polarity of the employed solvent. Specifically, **PQ**s with EWGs exhibit exceptional reactivity via the general photoclick pathway with a record-breaking second-order reaction rate of up to 11,470 M^–1^ s^–1^. In contrast, introducing EDGs blocks the conventional pathway but facilitates the TTET-mediated pathway with the help of a photosensitizer, thereby enabling efficient activation under green light. Moreover, the solvent polarity was also verified to play a decisive role in determining the reaction mechanisms, with low-polarity media predominantly supporting the general photoclick mechanism, whereas high polarity environments show TTET performance. Transient absorption spectroscopy supported by comprehensive computational analyses provides detailed mechanistic insight in the modulation of excitation energies of the ^1^ *n*π* and ^1^ππ* states under the influence of electronic and solvent polarity effects. As a result, ISC and triplet quantum yields are dominantly affected by these “tuning knobs”, resulting in different photoclick reaction pathways. This study not only illustrates the structure–mechanism relationship for the **PQ**-based photoclick reaction but also provides a solid foundation for the rational design of photoclick systems operable under long-wavelength visible light, thereby broadening the applicability of photoclick chemistry to complex biological and material systems. We envision that this novel ultrafast **PQ–ERA** photoclick reaction is not limited to the transformations shown in the present studies but will also be advantageous in a wide range of applications, such as surface photopatterning, labeling of biomacromolecules, and photochemical cross-linking.

## Supplementary Material



## Notes

Experimental procedures and characterization data for all new compounds, photophysical and chemical studies, and details regarding the computational calculation (PDF)

## References

[1] Montalti, M. ; Credi, A. ; Prodi, L. ; Gandolfi, M. T. ; Handbook of Photochemistry; CRC Press, 2006.

[2] Tornøe C. W., Christensen C., Meldal M. (2002). Peptidotriazoles on Solid Phase:
[1,2,3]-Triazoles by Regiospecific Copper­(I)-Catalyzed 1,3-Dipolar
Cycloadditions of Terminal Alkynes to Azides. J. Org. Chem..

[3] Rostovtsev V. V., Green L. G., Fokin V. V., Sharpless K. B. (2002). A Stepwise
Huisgen Cycloaddition Process: Copper­(I)-Catalyzed Regioselective
“Ligation” of Azides and Terminal Alkynes. Angew. Chem., Int. Ed..

[4] Kolb H. C., Finn M. G., Sharpless K. B. (2001). Click Chemistry:
Diverse Chemical
Function from a Few Good Reactions. Angew. Chem.,
Int. Ed..

[5] Agard N. J., Prescher J. A., Bertozzi C. R. (2004). A Strain-Promoted
[3 + 2] Azide–Alkyne
Cycloaddition for Covalent Modification of Biomolecules in Living
Systems. J. Am. Chem. Soc..

[6] Tasdelen M. A., Yagci Y. (2013). Light-Induced Click
Reactions. Angew. Chem.,
Int. Ed..

[7] Fairbanks B. D., Macdougall L. J., Mavila S., Sinha J., Kirkpatrick B. E., Anseth K. S., Bowman C. N. (2021). Photoclick Chemistry: A Bright Idea. Chem. Rev..

[8] Fu Y., Simeth N. A., Szymanski W., Feringa B. L. (2024). Visible and Near-Infrared
Light-Induced Photoclick Reactions. Nat. Rev.
Chem..

[9] Fu Y., Zhang X., Wu L., Wu M., James T. D., Zhang R. (2025). Bioorthogonally Activated Probes for Precise Fluorescence Imaging. Chem. Soc. Rev..

[10] Song W., Wang Y., Qu J., Lin Q. (2008). Selective Functionalization
of a Genetically Encoded Alkene-Containing Protein via “Photoclick
Chemistry” in Bacterial Cells. J. Am.
Chem. Soc..

[11] Hoyle C. E., Bowman C. N. (2010). Thiol-Ene Click Chemistry. Angew.
Chem., Int. Ed..

[12] Poloukhtine A. A., Mbua N. E., Wolfert M. A., Boons G.-J., Popik V. V. (2009). Selective
Labeling of Living Cells by a Photo-Triggered Click Reaction. J. Am. Chem. Soc..

[13] Zhang L., Zhang X., Yao Z., Jiang S., Deng J., Li B., Yu Z. (2018). Discovery
of Fluorogenic Diarylsydnone-Alkene Photoligation:
Conversion of Ortho -Dual-Twisted Diarylsydnones into Planar Pyrazolines. J. Am. Chem. Soc..

[14] Stuckhardt C., Wissing M., Studer A. (2021). Photo Click
Reaction of Acylsilanes
with Indoles. Angew. Chem., Int. Ed..

[15] Fu Y., Helbert H., Simeth N. A., Crespi S., Spoelstra G. B., van Dijl J. M., van Oosten M., Nazario L. R., van der
Born D., Luurtsema G., Szymanski W., Elsinga P. H., Feringa B. L. (2021). Ultrafast
Photoclick Reaction for Selective18F-Positron Emission Tomography
Tracer Synthesis in Flow. J. Am. Chem. Soc..

[16] Fu Y., Simeth N. A., Toyoda R., Brilmayer R., Szymanski W., Feringa B. L. (2023). Molecular Engineering To Enhance
Reactivity and Selectivity in an Ultrafast Photoclick Reaction. Angew. Chem., Int. Ed..

[17] Li J., Kong H., Huang L., Cheng B., Qin K., Zheng M., Yan Z., Zhang Y. (2018). Visible Light-Initiated
Bioorthogonal Photoclick Cycloaddition. J. Am.
Chem. Soc..

[18] Arumugam S., Orski S. V., Locklin J., Popik V. V. (2012). Photoreactive Polymer
Brushes for High-Density Patterned Surface Derivatization Using a
Diels–Alder Photoclick Reaction. J. Am.
Chem. Soc..

[19] Arumugam S., Popik V. V. (2012). Attach, Remove, or Replace: Reversible Surface Functionalization
Using Thiol–Quinone Methide Photoclick Chemistry. J. Am. Chem. Soc..

[20] Xie C., Sun W., Lu H., Kretzschmann A., Liu J., Wagner M., Butt H.-J., Deng X., Wu S. (2018). Reconfiguring Surface
Functions Using Visible-Light-Controlled Metal-Ligand Coordination. Nat. Commun..

[21] Truong V. X., Tsang K. M., Ercole F., Forsythe J. S. (2017). Red Light Activation
of Tetrazine-Norbornene Conjugation for Bioorthogonal Polymer Cross-Linking
across Tissue. Chem. Mater..

[22] Rizzo R., Ruetsche D., Liu H., Zenobi-Wong M. (2021). Optimized
Photoclick (Bio)­Resins for Fast Volumetric Bioprinting. Adv. Mater..

[23] Bailey S. J., Hopkins E., Rael K. D., Hashmi A., Urueña J. M., Wilson M. Z., Read
de Alaniz J. (2023). Design, Synthesis, and Application
of a Water-soluble Photocage for Aqueous Cyclopentadiene-based Diels-Alder
Photoclick Chemistry in Hydrogels. Angew. Chem.,
Int. Ed..

[24] Spoelstra G. B., Blok S. N., Reali
Nazario L., Noord L., Fu Y., Simeth N. A., IJpma F. F. A., van Oosten M., van Dijl J. M., Feringa B. L., Szymanski W., Elsinga P. H. (2024). Synthesis and Preclinical Evaluation
of Novel 18F-Vancomycin-Based
Tracers for the Detection of Bacterial Infections Using Positron Emission
Tomography. Eur. J. Nucl. Med. Mol. Imaging.

[25] Lang K., Davis L., Wallace S., Mahesh M., Cox D. J., Blackman M. L., Fox J. M., Chin J. W. (2012). Genetic Encoding
of Bicyclononynes and Trans -Cyclooctenes for Site-Specific Protein
Labeling in Vitro and in Live Mammalian Cells via Rapid Fluorogenic
Diels–Alder Reactions. J. Am. Chem. Soc..

[26] An P., Lewandowski T. M., Erbay T. G., Liu P., Lin Q. (2018). Sterically
Shielded, Stabilized Nitrile Imine for Rapid Bioorthogonal Protein
Labeling in Live Cells. J. Am. Chem. Soc..

[27] Kumar G. S., Lin Q. (2021). Light-Triggered Click Chemistry. Chem. Rev..

[28] Davis A. R., Maegerlein J. A., Carter K. R. (2011). Electroluminescent Networks via Photo
“Click” Chemistry. J. Am. Chem.
Soc..

[29] Lim R. K. V., Lin Q. (2010). Azirine Ligation: Fast and Selective Protein Conjugation
via Photoinduced Azirine–Alkene Cycloaddition. Chem. Commun..

[30] Pauloehrl T., Delaittre G., Bruns M., Meißler M., Börner H. G., Bastmeyer M., Barner-Kowollik C. (2012). (Bio)­Molecular
Surface Patterning by Phototriggered Oxime Ligation. Angew. Chem., Int. Ed..

[31] Zhang Z., Wang W., O’Hagan M., Dai J., Zhang J., Tian H. (2022). Stepping Out of the Blue: From Visible
to Near-IR Triggered Photoswitches. Angew. Chem.,
Int. Ed..

[32] Kuntze K., Viljakka J., Virkki M., Huang C. Y., Hecht S., Priimagi A. (2023). Red-Light Photoswitching of Indigos in Polymer Thin
Films. Chem. Sci..

[33] Wegener M., Hansen M. J., Driessen A. J. M., Szymanski W., Feringa B. L. (2017). Photocontrol of Antibacterial Activity:
Shifting from
UV to Red Light Activation. J. Am. Chem. Soc..

[34] Bléger D., Hecht S. (2015). Visible-Light-Activated Molecular Switches. Angew. Chem., Int. Ed..

[35] Moreno J., Gerecke M., Grubert L., Kovalenko S. A., Hecht S. (2016). Sensitized Two-NIR-Photon Z→E
Isomerization of a Visible-Light-Addressable
Bistable Azobenzene Derivative. Angew. Chem.,
Int. Ed..

[36] Klaue K., Han W., Liesfeld P., Berger F., Garmshausen Y., Hecht S. (2020). Donor-Acceptor Dihydropyrenes Switchable with Near-Infrared Light. J. Am. Chem. Soc..

[37] Weinstain R., Slanina T., Kand D., Klán P. (2020). Visible-to-NIR-Light
Activated Release: From Small Molecules to Nanomaterials. Chem. Rev..

[38] Szymański W., Beierle J. M., Kistemaker H. A. V., Velema W. A., Feringa B. L. (2013). Reversible
Photocontrol of Biological Systems by the Incorporation of Molecular
Photoswitches. Chem. Rev..

[39] An P., Yu Z., Lin Q. (2013). Design of
Oligothiophene-Based Tetrazoles for Laser-Triggered
Photoclick Chemistry in Living Cells. Chem.
Commun..

[40] Lederhose P., Wüst K. N. R., Barner-Kowollik C., Blinco J. P. (2016). Catalyst Free Visible
Light Induced Cycloaddition as an Avenue for Polymer Ligation. Chem. Commun..

[41] Kodura D., Rodrigues L. L., Walden S. L., Goldmann A. S., Frisch H., Barner-Kowollik C. (2022). Orange-Light-Induced
Photochemistry Gated by PH and
Confined Environments. J. Am. Chem. Soc..

[42] Yu Z., Ohulchanskyy T. Y., An P., Prasad P. N., Lin Q. F. (2013). Two-Photon-Triggered
Photoclick Chemistry in Live Mammalian Cells. J. Am. Chem. Soc..

[43] Lederhose P., Chen Z., Müller R., Blinco J. P., Wu S., Barner-Kowollik C. (2016). Near-Infrared
Photoinduced Coupling Reactions Assisted
by Upconversion Nanoparticles. Angew. Chem.,
Int. Ed..

[44] Wu Y., Zheng J., Xing D., Zhang T. (2020). Near-Infrared Light
Controlled Fluorogenic Labeling of Glycoengineered Sialic Acids in
Vivo with Upconverting Photoclick Nanoprobe. Nanoscale.

[45] Doze A. M., Fu Y., Di Donato M., Hilbers M. F., Luurtsema G., Elsinga P. H., Buma W. J., Szymanski W., Feringa B. L. (2024). With or without a Co-Solvent? Highly Efficient Ultrafast
Phenanthrenequinone-Electron Rich Alkene (PQ-ERA) Photoclick Reactions. Chem. Sci..

[46] Fu Y., Alachouzos G., Simeth N. A., Di Donato M., Hilbers M. F., Buma W. J., Szymanski W., Feringa B. L. (2024). Triplet-Triplet Energy Transfer:
A Simple Strategy
for an Efficient Visible Light-Induced Photoclick Reaction. Angew. Chem., Int. Ed..

[47] Fu Y., Wu K., Alachouzos G., Simeth N. A., Freese T., Falkowski M., Szymanski W., Zhang H., Feringa B. L. (2023). Efficient, Near-Infrared
Light-Induced Photoclick Reaction Enabled by Upconversion Nanoparticles. Adv. Funct. Mater..

[48] Fu Y., Alachouzos G., Simeth N. A., Di Donato M., Hilbers M. F., Buma W. J., Szymanski W., Feringa B. L. (2023). Establishing PQ-ERA Photoclick Reactions
with Unprecedented
Efficiency by Engineering of the Nature of the Phenanthraquinone Triplet
State. Chem. Sci..

[49] Huang L., Han G. (2024). Triplet–Triplet
Annihilation Photon Upconversion-Mediated
Photochemical Reactions. Nat. Rev. Chem..

[50] Hou L., Ringström R., Maurer A. B., Abrahamsson M., Andréasson J., Albinsson B. (2022). Optically Switchable NIR Photoluminescence
of PbS Semiconducting Nanocrystals Using Diarylethene Photoswitches. J. Am. Chem. Soc..

[51] Hou L., Larsson W., Hecht S., Andréasson J., Albinsson B. (2022). A General Approach for All-Visible-Light
Switching
of Diarylethenes through Triplet Sensitization Using Semiconducting
Nanocrystals. J. Mater. Chem. C.

[52] Zhao W., Cheung T. S., Jiang N., Huang W., Lam J. W. Y., Zhang X., He Z., Tang B. Z. (2019). Boosting the Efficiency
of Organic Persistent Room-Temperature Phosphorescence by Intramolecular
Triplet-Triplet Energy Transfer. Nat. Commun..

[53] Fredrich S., Göstl R., Herder M., Grubert L., Hecht S. (2016). Switching
Diarylethenes Reliably in Both Directions with Visible Light. Angew. Chem., Int. Ed..

[54] Zhang Z., Zhang J., Wu B., Li X., Chen Y., Huang J., Zhu L., Tian H. (2018). Diarylethenes
with
a Narrow Singlet–Triplet Energy Gap Sensitizer: A Simple Strategy
for Efficient Visible-Light Photochromism. Adv.
Opt. Mater..

[55] Zhang Z., Wang W., Jin P., Xue J., Sun L., Huang J., Zhang J., Tian H. (2019). A Building-Block
Design
for Enhanced Visible-Light Switching of Diarylethenes. Nat. Commun..

[56] Bortolus P., Monti S. (1979). Cis-Trans Photoisomerization
of Azobenzene. Solvent and Triplet Donors
Effects. J. Phys. Chem..

[57] Isokuortti J., Kuntze K., Virkki M., Ahmed Z., Vuorimaa-Laukkanen E., Filatov M. A., Turshatov A., Laaksonen T., Priimagi A., Durandin N. A. (2021). Expanding Excitation
Wavelengths
for Azobenzene Photoswitching into the Near-Infrared Range via Endothermic
Triplet Energy Transfer. Chem. Sci..

[58] Gemen J., Church J. R., Ruoko T.-P., Durandin N., Białek M. J., Weißenfels M., Feller M., Kazes M., Odaybat M., Borin V. A. (2023). Disequilibrating Azobenzenes by Visible-Light
Sensitization under Confinement. Science.

[59] Cnossen A., Hou L., Pollard M. M., Wesenhagen P. V., Browne W. R., Feringa B. L. (2012). Driving
Unidirectional Molecular Rotary Motors with Visible Light by Intra-
And Intermolecular Energy Transfer from Palladium Porphyrin. J. Am. Chem. Soc..

[60] Danowski W., Castiglioni F., Sardjan A. S., Krause S., Pfeifer L., Roke D., Comotti A., Browne W. R., Feringa B. L. (2020). Visible-Light-Driven
Rotation of Molecular Motors in a Dual-Function Metal–Organic
Framework Enabled by Energy Transfer. J. Am.
Chem. Soc..

[61] Miyaura N., Yamada K., Suzuki A. (1979). A New Stereospecific Cross-Coupling
by the Palladium-Catalyzed Reaction of 1-Alkenylboranes with 1-Alkenyl
or 1-Alkynyl Halides. Tetrahedron Lett..

[62] Schulte A. M., Alachouzos G., Szymański W., Feringa B. L. (2022). Strategy for Engineering
High Photolysis Efficiency of Photocleavable Protecting Groups through
Cation Stabilization. J. Am. Chem. Soc..

[63] Alachouzos G., Schulte A. M., Mondal A., Szymanski W., Feringa B. L. (2022). Computational Design, Synthesis, and Photochemistry
of Cy7-PPG, an Efficient NIR-Activated Photolabile Protecting Group
for Therapeutic Applications**. Angew. Chem.,
Int. Ed..

[64] van
Vliet S., Alachouzos G., de Vries F., Pfeifer L., Feringa B. L. (2022). Visible Light Activated BINOL-Derived Chiroptical Switches
Based on Boron Integrated Hydrazone Complexes. Chem. Sci..

[65] Marenich A. V., Cramer C. J., Truhlar D. G. (2009). Universal Solvation Model Based on
Solute Electron Density and on a Continuum Model of the Solvent Defined
by the Bulk Dielectric Constant and Atomic Surface Tensions. J. Phys. Chem. B.

[66] Zheng J., Xu X., Truhlar D. G. (2011). Minimally
Augmented Karlsruhe Basis Sets. Theor. Chem.
Acc..

[67] Yu H. S., He X., Li S. L., Truhlar D. G. (2016). MN15: A
Kohn–Sham Global-Hybrid
Exchange–Correlation Density Functional with Broad Accuracy
for Multi-Reference and Single-Reference Systems and Noncovalent Interactions. Chem. Sci..

[68] Lower S. K., El-Sayed M. A. (1966). The Triplet State
and Molecular Electronic Processes
in Organic Molecules. Chem. Rev..

[69] Zhang J., Tu Y., Shen H., Lam J. W. Y., Sun J., Zhang H., Tang B. Z. (2023). Regulating
the Proximity Effect of Heterocycle-Containing
AIEgens. Nat. Commun..

