# Supplementary material for: Mechanism Inversion in Visible Light-Induced Photoclick Reactions
Source: J Am Chem Soc. 2025 Sep 19;147(39):35903–12. doi: 10.1021/jacs.5c12759 (PMC12498399; doi:10.1021/jacs.5c12759)
Supplement: Supplementary file 1 [file ja5c12759_si_001.pdf]

Supporting Information

Mechanism inversion in visible light-induced photoclick reactions

Youxin Fu,[a,b],†,* Jingwen Zhou,[a,b],†, Xinyi Zou,[a] Liuhui Shi,[a] Xing Zhang,[c] Anna M. Doze,[b] Michiel F. Hilbers,[d] Wybren Jan Buma,[d,e] Jianyu Zhang,[b],* and Ben L. Feringa[b],*

[a]College of Science, Nanjing Forestry University, Nanjing, 210037, P.R. China.

[b]Centre for Systems Chemistry, Stratingh Institute for Chemistry, Faculty for Science and Engineering, University of Groningen, Nijenborgh 4, 9747 AG Groningen, The Netherlands.

[c]Co-Innovation Center for Sustainable Forestry in Southern China, College of Ecology and Environment, Nanjing Forestry University, Nanjing 210037, China.

[d]Van't Hoff Institute for Molecular Sciences, University of Amsterdam Science Park 904, 1098 XH Amsterdam, The Netherlands.

[e]Institute for Molecules and Materials, FELIX Laboratory, Radboud University, Toernooiveld 7c, 6525 ED Nijmegen, The Netherlands.

Table of Contents

1. General Information 3
2. Synthesis of PQ compounds and photoclick products 6
  2.1. Synthesis of compound PQ-2DiI 7
  2.2. Synthesis of PQ-CF_3 8
  2.3. Synthesis of PQ-CHO 8
  2.4. Synthesis of PQ-$COCH_3$ 8
  2.5. Synthesis of PQ-H 9
  2.6. Synthesis of PQ-^{t}Bu 9
  2.7. Synthesis of PQ-CH_3 9
  2.8. Synthesis of PQ-OCH_3 9
  2.9. Synthesis of DiIbodipy 10
  2.10. Synthesis of PQ-CF_3-PY 11
  2.11. Synthesis of PQ-CHO-PY 12
  2.12. Synthesis of PQ-$COCH_3$-PY 12
  2.13. Synthesis of PQ-H-PY 12
  2.14. Synthesis of PQ-^{t}Bu-PY 13
  2.15. Synthesis of PQ-CH_3-PY 13
  2.16. Synthesis of PQ-OCH_3-PY 13
3. NMR Spectra 15
4. Photophysical and Photochemical Studies by UV-Vis Spectroscopy and Transient Absorption Spectroscopy 32
  4.1. UV-Vis Spectra 32
  4.2. Analysis of reactions rates 32
  4.3. Nanosecond Transient Absorption Spectroscopy 51
5. Computational analysis 54
6. References 60

1. General Information

Synthesis and isolation. Solvents used were of analytical grade. All other chemicals were used as received unless otherwise indicated. Deionized water was used throughout. All oxygen or moisture-sensitive reactions were performed in dried glassware and under N_2 atmosphere using standard Schlenk techniques. Dry solvents were obtained from an MBraun solvent purification system (SPS). Column chromatography was performed on silica gel (Silica 60 M, 0.04-0.063 mm.

Reaction monitoring. Reactions were monitored using thin-layer chromatography (TLC) on aluminium sheets coated with silica gel 60 F254 (MERCK). Components were visualized by UV-light (254 nm, 365 nm) and potassium permanganate or Seebach staining.

Analysis. Mass spectra were recorded on an AEI-MS-902 mass spectrometer (EI+) or a LTQ Orbitrap XL (ESI+, ESI-, APCI+). ^{1}H- and ^{13}C NMR were recorded on Bruker AM-400 Spectrometer (400 MHz and 100.59 MHz, respectively using DMSO-d_6 as solvent. Data are reported as follows: chemical shifts, multiplicity (s = singlet, d = doublet, t = triplet, q = quartet, m = multiplet), coupling constants (Hz), and integration. UV-Vis spectra were recorded on a SHIMADZU UV-2700i UV-Visible spectrophotometer in a quartz cuvette with 1 cm pathlength at 20 °C. Commercially available LEDs (power intensity: 3W, 440 nm, 455 nm, 520 nm) was used as the light source for the **PQ-ERA** photoclick reaction. The detailed emission profile of LEDs can be found in Fig. S1-S3.

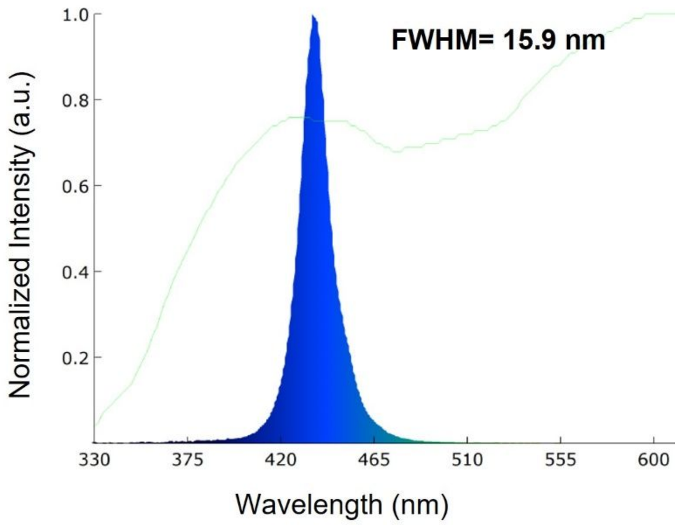

Fig. S1. Emission spectra of 440 nm LED.

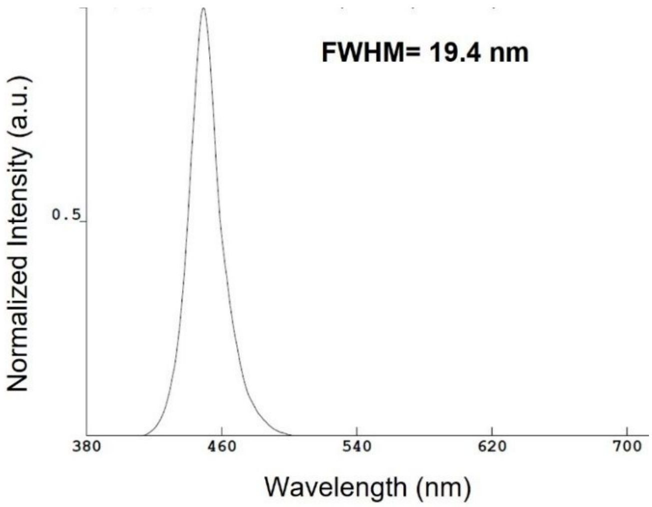

Fig. S2. Emission spectra of 455 nm LED.

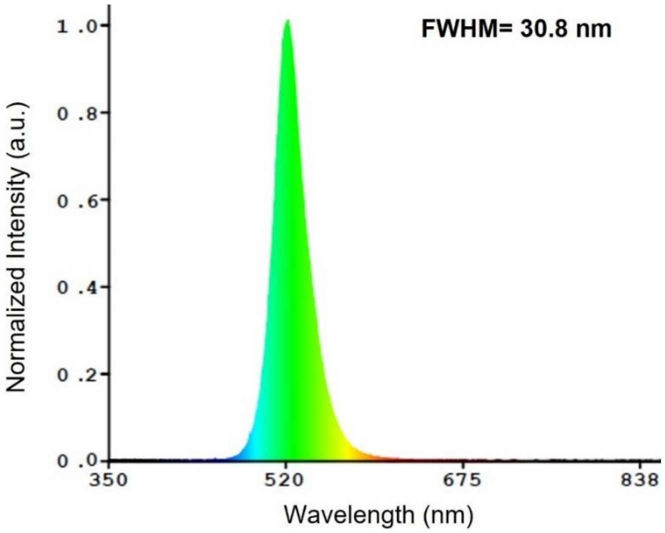

Fig. S3. Emission spectra of 520 nm LED.

Nanosecond Transient Absorption Spectroscopy. Nanosecond transient absorption spectra have been recorded using the setup described previously.[1–3] As a general rule, samples were prepared with an optical density between 1.0 and 1.5, while transient absorption spectra were taken using excitation at the wavelength of maximum absorption using a pulse energy of 0.1 mJ. In some cases, poor solubility and/or low triplet quantum yields constrained us to use a lower optical density and/or higher pulse energies. We therefore specify for all spectra shown in the main manuscript and in the Supporting Information the employed optical density and pulse energies. All data have been analyzed using a global fit procedure as implemented in Glotaran.[4] Since our previous studies showed that -as also observed in the present study- excitation of PQs is accompanied by the formation of the pertaining ketyl radical, a parallel decay kinetic scheme has been used, leading to Decay Associated Difference Spectra (DADS) and rate constants that are reported in the main manuscript and in the Supporting Information.

Computational methods. All theoretical calculations of PQ derivatives were carried out based on the density functional theory methods. The ground-state geometries are optimized without any symmetry constraints at the MN15/Def2-TZVP level, which was utilized to evaluate energy levels with comparatively high accuracy.[1,5] The geometries of excited singlet and triplet were all optimized using the time-dependent DFT (TD-DFT) method at the same level of theory. To investigate the solvent effect on the energy level and electron transition of these compounds, the solvation model based on the polarizable continuum model (PCM) and self-consistent reaction field (SCRF) was considered in the calculations with the solvents of acetonitrile, toluene, and ethyl acetate, respectively.[6] Analytical frequency calculations were also performed at the same level of theory to confirm that the optimized structures were at a minimum point. The adiabatic and vertical energy levels were summarized based on their lowest ground- and excited-state geometries. All the above calculations were carried out using Gaussian 16 program (Revision A.03).[7] The hole-electron analysis and frontier molecular orbitals were analysis using Multiwfn (Version 3.8)[8,9] and displayed using the IQmol molecular viewer

package (Version 3.0.1).[10] All coordinates of optimized geometries of PQ derivatives are provided in the corresponding excel file as the supporting file.

Photocycloaddition of PQs with traps in solution. Stock solutions of **PQs** (10 mM, in DMSO), and *N*-boc-2,3-dihydro-1*H*-pyrrole (**PY**, 100 mM, in MeCN), were prepared respectively. From them, a solution of **PQs/PY** (50/500) was prepared in MeCN (2.0 mL) in a quartz cuvette and degassed by N_2 for 3 min. 420 nm, 440 nm, 455nm, and 520 nm LED were used as light source for photoclick reactions and positioned at a fixed distance to the cuvette. Changes in the absorption were monitored by UV-Visible spectrophotometer. The setup the irradiation system were shown below (455 nm LED was used as an example, Fig. S4 and S5).

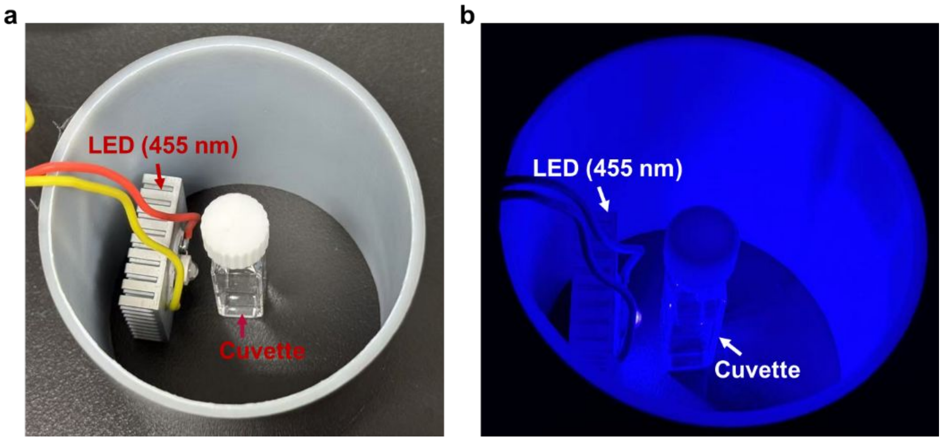

Fig. S4. Photograph of LED-system.

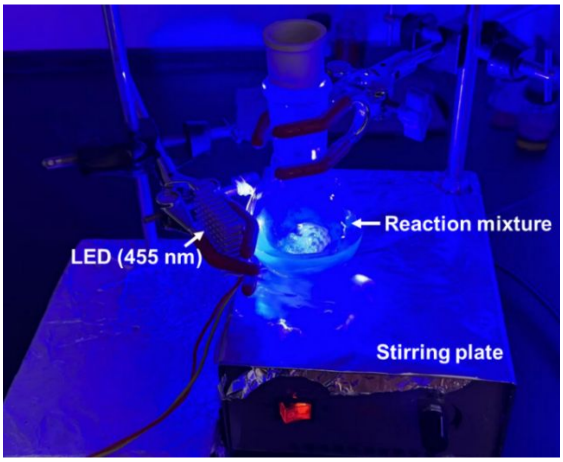

Fig. S5. Photograph of the **PQ-ERA** reaction setup.

2. Synthesis of PQ compounds and photoclick products

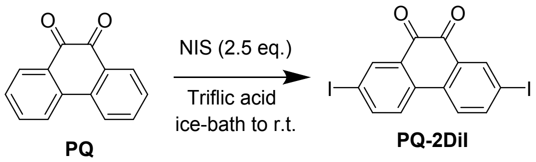

Scheme S1. Synthesis of **PQ-2DiI**.

2.1. Synthesis of compound PQ-2DiI

To 9,10-Phenanthrenequinone (1.6 g, 7.8 mmol), trifluoromethanesulfonic acid (10.1 mL, 120 mmol) was added in a round bottom flask under nitrogen atmosphere and the resulting mixture was cooled to 0°C. *N*-Iodosuccinimide (3.5 g, 15 mmol) was added slowly over 3 min and the reaction mixture was allowed to gradually warm to room temperature. After 6 h at room temperature, the reaction mixture was poured onto ice, filtered and dried to afford **PQ-2DiI** (3.35 g, 7.3 mmol) in 95% yield as an orange solid. ^{1}H NMR (400 MHz, DMSO-d_6) δ = 8.22 (d, J = 1.8 Hz, 2H), 8.08 (dd, J = 8.4, 1.8 Hz, 2H), 8.03 (d, J = 8.5 Hz, 2H). ^{13}C NMR (101 MHz, DMSO-d_6) δ =177.3, 143.6, 137.5, 134.5, 133.4, 126.9, 96.4, 96.4. The obtained data is in accordance with the literature.[1]

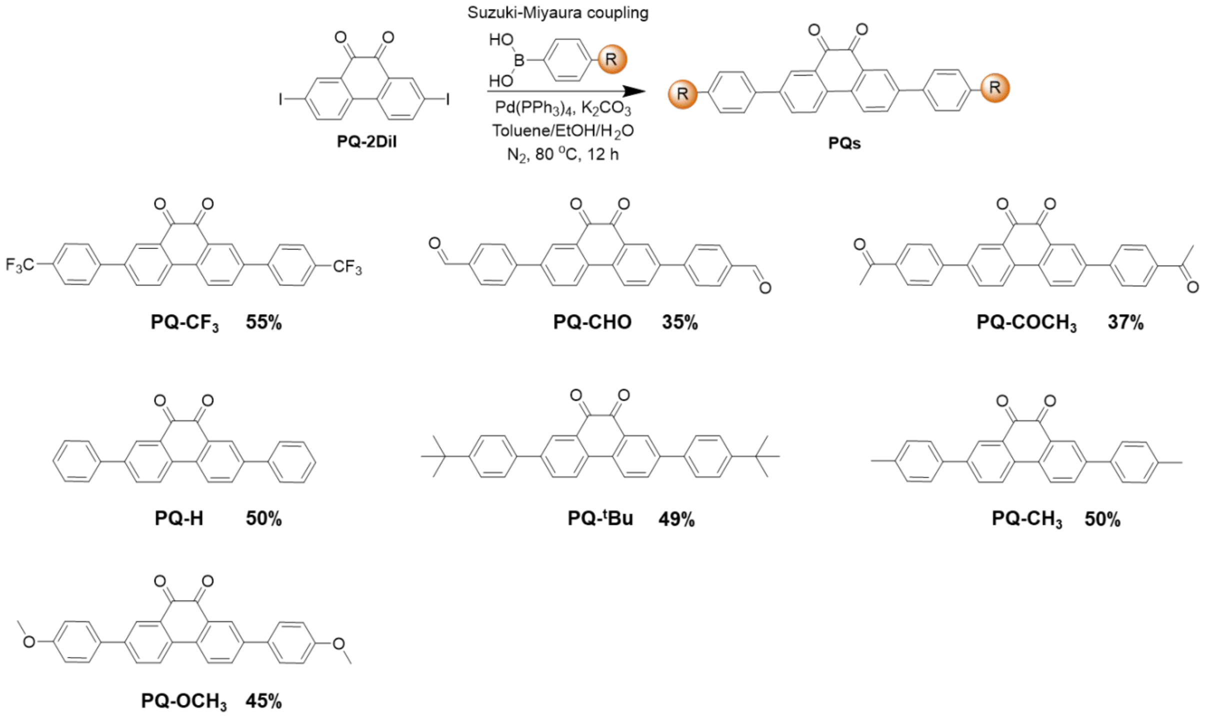

Scheme S2. Synthesis of **PQs.**

2.2. Synthesis of PQ-CF_3

A Schlenk tube containing **PQ-2DiI** (322 mg, 0.7 mmol), and (4 (trifluoromethyl)phenyl)boronic acid (285 mg, 0.15 mmol), and $Pd(PPh_3)_4$ (46 mg, 0.04 mmol) was evacuated and backfilled with N_2 for three times. After the addition of degassed K_2CO_3 solution (304 mg in 3.5 mL H_2O), ethanol (4 ml), and toluene (15 mL), the tube was sealed under N_2 atmosphere and heated at 80 °C for 12 h. After being cooled to room temperature, the reaction mixture was diluted with dichloromethane, the phases were separated and the organic layer was washed with water and dried over anhydrous Na_2SO_4. Upon removal of solvents in vacuo, the residual was purified by column chromatography over silica gel eluted with ethyl acetate/dichloromethane (1/5, v/v) to obtain **PQ-CF_3** as an orange solid in 55% yield (0.39 mmol, 191 mg). ^{1}H NMR (400 MHz, *$CDCl_3$*) δ 8.49 (d, *J* = 2.1 Hz, 2H), 8.20 (d, *J* = 8.4 Hz, 2H), 8.02 (dd, *J* = 8.3, 2.1 Hz, 2H), 7.81 (q, *J* = 8.5 Hz, 8H). ^{13}C NMR (151 MHz, *$CDCl_3$*) δ 179.9, 141.9, 141.1, 135.0, 134.4, 131.5, 129.0, 127.2, 126.1, 125.0.

2.3. Synthesis of PQ-CHO

The title compound was synthesized using the same method as for **PQ-CF_3** employing (4-formylphenyl)boronic acid (1.63 mmol, 245 mg) to obtain **PQ-CHO** as a dark red solid in 35% yield (0.23 mmol, 95 mg). ^{1}H NMR (400 MHz, DMSO-*d_6*) δ 10.10 (s, 2H), 8.54 (d, *J* = 8.1 Hz, 2H), 8.40 (s, 2H), 8.25 (d, *J* = 5.6 Hz, 2H), 8.09 (d, *J* = 2.8 Hz, 8H). ^{13}C NMR (151 MHz, *$CDCl_3$*) δ 180.5, 143.4, 139.5, 136.8, 135.2, 134.3, 131.1, 130.8, 130.7, 129.5, 128.3, 126.2, 123.9, 20.4.

2.4. Synthesis of PQ-$COCH_3$

The title compound was synthesized using the same method as for **PQ-CF_3** employing (4-acetylphenyl)boronic acid (1.63 mmol, 267 mg) to obtain **PQ-$COCH_3$** as a dark red solid in 37% yield (0.24 mmol, 107 mg). ^{1}H NMR (400 MHz, DMSO-*d_6*) δ 8.52 (d, *J* = 8.3 Hz, 2H), 8.38 (s, 2H), 8.23 (d, *J* = 9.2 Hz, 2H), 8.12 (d, *J* = 8.3 Hz, 4H), 8.00 (d, *J* = 8.4 Hz, 4H), 2.65

(s, 6H). ^{13}C NMR (151 MHz, *CDCl$_3$*) δ 197.4, 142.8, 136.9, 135.0, 134.3, 131.5, 129.16, 127.0, 124.9, 29.7, 26.7.

2.5. Synthesis of PQ-H

The title compound was synthesized using the same method as for **PQ-CF$_3$** employing phenylboronic acid (1.63 mmol, 199 mg) to obtain **PQ-H** as a dark red solid in 50% yield (0.33 mmol, 117 mg). ^1H NMR (400 MHz, *CDCl$_3$*) δ 8.47 (d, J = 2.1 Hz, 2H), 8.14 (d, J = 8.3 Hz, 2H), 8.00 (dd, J = 8.3, 2.1 Hz, 2H), 7.72 (d, J = 7.1 Hz, 4H), 7.53 (t, J = 7.4 Hz, 4H), 7.45 (t, J = 7.3 Hz, 2H). ^{13}C NMR (151 MHz, *CDCl$_3$*) δ 142.3, 138.6, 134.3, 129.1, 128.8, 128.5, 126.9, 124.7.

2.6. Synthesis of PQ-^{t}Bu

The title compound was synthesized using the same method as for **PQ-CF$_3$** employing (4-(tert-butyl)phenyl)boronic acid (1.63 mmol, 290 mg) to obtain **PQ-^{t}Bu** as a dark red solid in 50% yield (0.33 mmol, 154 mg). ^1H NMR (400 MHz, DMSO-d_6) δ 8.42 (d, J = 8.5 Hz, 2H), 8.27 (d, J = 1.9 Hz, 2H), 8.11 (dd, J = 8.2, 2.0 Hz, 2H), 7.75 (d, J = 8.3 Hz, 4H), 7.56 (d, J = 8.3 Hz, 4H), 1.35 (s, 18H). ^{13}C NMR (151 MHz, *CDCl$_3$*) δ 180.5, 151.8, 142.0, 135.6, 134.3, 134.1, 131.2, 128.5, 126.5, 126.1, 124.6, 34.7, 31.3.

2.7. Synthesis of PQ-CH$_3$

The title compound was synthesized using the same method as for **PQ-CF$_3$** employing p-tolylboronic acid (1.63 mmol, 222 mg) to obtain **PQ-CH$_3$** as a dark red solid in 49% yield (0.32 mmol, 124 mg). ^1H NMR (400 MHz, DMSO-d_6) δ 8.42 (d, J = 8.3 Hz, 2H), 7.98 – 7.93 (m, 2H), 7.82 (d, J = 8.2 Hz, 2H), 7.35 (dd, J = 12.1, 5.2 Hz, 8H), 2.32 (s, 6H). ^{13}C NMR (151 MHz, *CDCl$_3$*) δ 180.5, 142.1, 138.5, 135.6, 134.3, 134.0, 131.1, 129.8, 128.4, 126.6, 124.6, 21.2.

2.8. Synthesis of PQ-OCH$_3$

The title compound was synthesized using the same method as for **PQ-CF$_3$** employing (4-methoxyphenyl)boronic acid (1.63 mmol, 248 mg) to obtain **PQ-OCH$_3$** as a dark red solid in

45% yield (0.29 mmol, 123 mg). ^{1}H NMR (400 MHz, DMSO-d_6) δ 8.38 (d, J = 8.5 Hz, 2H), 8.23 (d, J = 2.1 Hz, 2H), 8.08 (dd, J = 8.4, 2.1 Hz, 2H), 7.78 (d, J = 8.8 Hz, 4H), 7.10 (d, J = 8.8 Hz, 4H), 3.84 (s, 6H). ^{13}C NMR (101 MHz, DMSO-d_6) δ 179.51, 160.07, 140.59, 133.99, 133.11, 131.99, 130.82, 128.18, 126.40, 125.75, 115.10, 55.73. HR-MS (ESI) *m/z*, calculated for $[M+H]^+$: 421.1434 ; found: 421.1430.

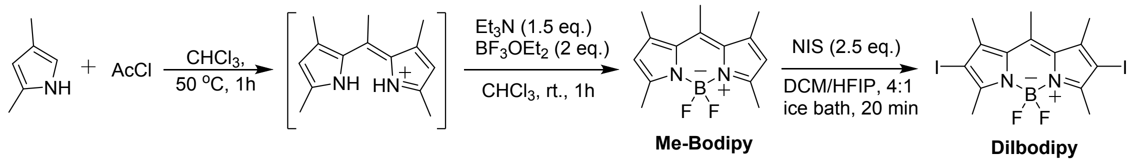

Scheme S3. Synthesis of **DiIbodipy.**

2.9. Synthesis of DiIbodipy

Acetyl chloride (108 mg, 1.38 mmol) was added to a solution of 2,4-dimethylpyrrole (238 mg, 2.50 mmol) in chloroform (2.5 mL) and the resulting mixture was heated to 50 °C for 1 h. Then, the reaction was cooled in an ice-water bath, and Et_3N (522 µL, 3.75 mmol) was added dropwise, followed by BF_3OEt_2 (620 µL, 5.00 mmol) dropwise. The reaction mixture was stirred at room temperature for 1 h, and then sat. $NaHCO_3$ (10 mL) was added, along with dichloromethane (10 mL). The layers were separated, and the aqueous layer was extracted once more with dichloromethane (10 mL). The combined organic layers were dried over $MgSO_4$, filtered, and concentrated. The residue was purified by column chromatography over silica gel eluted with toluene/pentane to yield **Me-Bodipy** as a dark brown powder (171 mg, 0.65 mmol, 52% yield). ^{1}H NMR (400 MHz, *$CDCl_3$*) δ = 6.05 (s, 2H), 2.57 (s, 3H), 2.52 (s, 6H), 2.41 (s, 6H). ^{19}F NMR (376 MHz, *$CDCl_3$*) δ = -146.69. The obtained data is in accordance with the literature.[11]

N-Iodosuccinimide (337 mg, 1.50 mmol) was added to a solution of **Me-Bodipy** (157 mg, 0.6 mmol) in dichloromethane (4.8 mL) and hexafluoroisopropanol (1.2 mL) and the solution was cooled in an ice-water bath and stirred for 20 min. Sat. $Na_2S_2O_3$ (10 mL) was added, along with Et_2O (20 mL). The layers were separated, and the organic layer was washed once more with sat. $NaHCO_3$ (aqueous, 10 mL). The organic layer was separated, dried over $MgSO_4$, filtered

and concentrated to yield **DiIbodipy** as a deep red powder (256 mg, 0.50 mmol, 83% yield). ^{1}H NMR (400 MHz, *CDCl$_3$*) δ = 2.64 (s, 3H), 2.61 (s, 6H), 2.47 (s, 6H). ^{19}F NMR (376 MHz, *CDCl$_3$*) δ = -145.9. The obtained data is in accordance with the literature.[1,12]

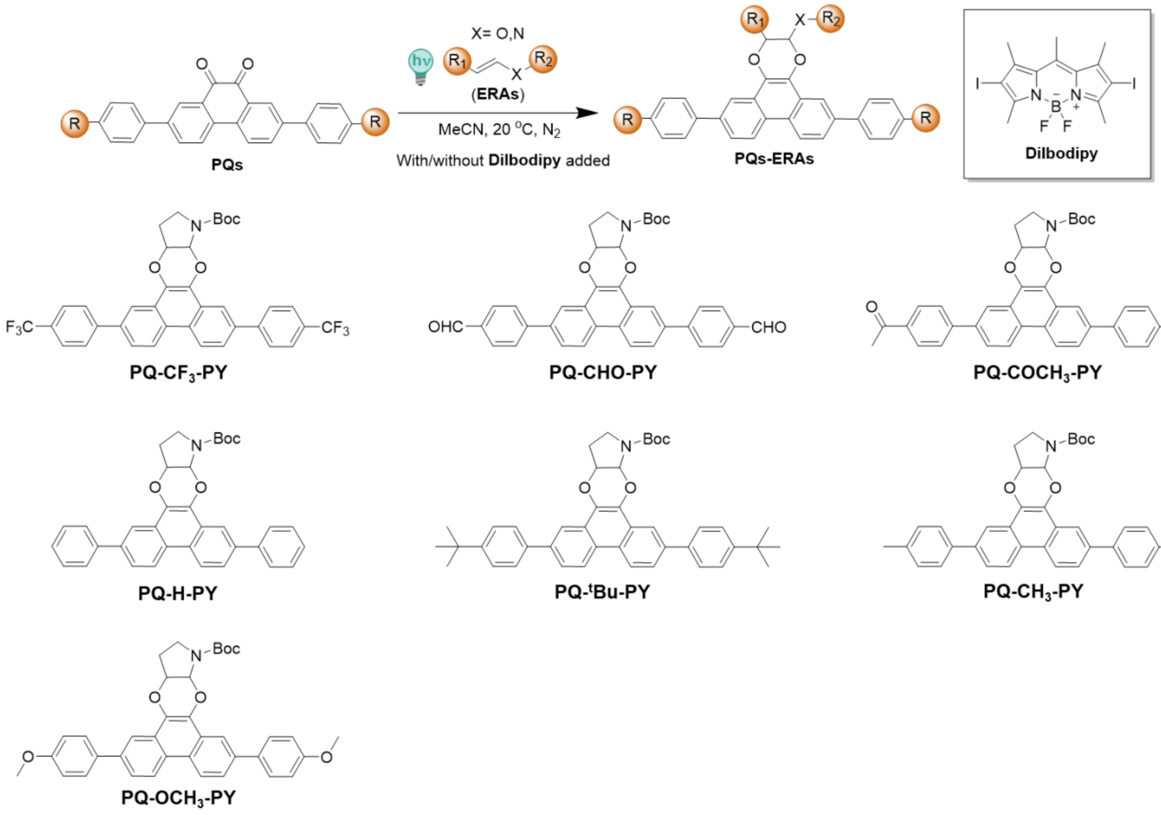

Scheme S4. Synthesis of **PQs-PY** derivatives.

2.10. Synthesis of PQ-CF$_3$-PY

PQ-CF$_3$ (0.065 mmol, 32 mg) and **PY** (0.67 mmol, 113 mg) were dissolved in MeCN (20 mL). Then, the mixture was stirred and irradiated using LED lamp (440 nm, 3 W, λ_{max} = 436.5 nm, FWHM 15.9 nm) at a fixed distance for 1 cm under N_2 atmosphere. The reaction was monitored by TLC. After completion, the volatiles were evaporated and the resulting residue was purified by column chromatography on silica gel (dichloromethane/ethyl acetate = 1:4, v/v) to afford **PQ-CF$_3$-PY** as a colorless powder (30 mg, 0.046 mmol, 70 % yield). ^{1}H NMR (400 MHz, DMSO-*d$_6$*) δ 8.97 (d, *J* = 8.5 Hz, 2H), 8.41 (s, 2H), 8.10 (d, *J* = 8.1 Hz, 2H), 8.04 (t, *J* = 8.8 Hz, 4H), 7.91 (t, *J* = 7.6 Hz, 4H), 5.76 (s, 1H), 5.30 – 5.22 (m, 1H), 3.67 – 3.54 (m, 2H), 2.12 – 1.95 (m, 2H), 1.52 (s, 9H). ^{13}C NMR (101 MHz, DMSO-*d$_6$*) δ 153.7, 143.5, 132.8, 129.3, 126.8,

125.7, 125.6, 124.9, 124.8, 124.6, 124.5, 123.5, 116.6, 28.6. HR-MS (ESI) *m/z*, calculated for $[M+H]^+$: 666.2074; found: 666.2016.

2.11. Synthesis of PQ-CHO-PY

The title compound was synthesized using the same method as for **PQ-CF$_3$-PY**, employing **PQ-CHO** (0.1 mmol, 42 mg) to afford **PQ-CHO-PY** as a colorless powder (29 mg, 0.05 mmol, 50 % yield). ^{1}H NMR (400 MHz, DMSO-d_6) δ 10.11 (s, 2H), 8.98 (d, J = 8.5 Hz, 2H), 8.45 (d, J = 1.5 Hz, 2H), 8.09 (t, J = 8.6 Hz, 10H), 5.76 (s, 1H), 5.33 – 5.23 (m, 1H), 3.67 – 3.55 (m, 1H), 3.52 – 3.35 (m, 1H), 2.15 – 1.95 (m, 2H), 1.53 (s, 9H). ^{13}C NMR (151 MHz, DMSO-d_6) δ 153.7, 141.5, 140.5, 135.3, 130.9, 130.2, 130.1, 128.1, 127.1, 126.9, 126.5, 126.1, 125.71, 125.4, 125.3, 123.4, 120.7, 28.4, 20.7. HR-MS (ESI) *m/z*, calculated for $[M+H]^+$: 586.2225; found: 586.2278.

2.12. Synthesis of PQ-COCH$_3$-PY

The title compound was synthesized using the same method as for **PQ-CF$_3$-PY**, employing **PQ-COCH$_3$** (0.1 mmol, 44 mg) to afford **PQ-COCH$_3$-PY** as a colorless powder (31 mg, 0.051 mmol, 51 % yield). ^{1}H NMR (400 MHz, DMSO-d_6) δ 8.97 (d, J = 9.1 Hz, 2H), 8.47 – 8.40 (m, 2H), 8.13 (dd, J = 8.5, 2.7 Hz, 4H), 8.04 (t, J = 7.7 Hz, 4H), 8.01 – 7.95 (m, 2H), 5.76 (s, 1H), 5.30 – 5.23 (m, 1H), 3.65 – 3.39 (m, 2H), 2.66 (s, 6H), 1.98 (d, J = 8.1 Hz, 2H), 1.54 (s, 9H). ^{13}C NMR (151 MHz, *CDCl$_3$*) δ 197.7, 153.9, 145.5, 136.1, 128.9, 127.5, 126.8, 126.5, 126.3, 126.2, 124.3, 123.5, 119.4, 81.1, 29.6, 28.4, 26.6. HR-MS (ESI) *m/z*, calculated for $[M+H]^+$: 614.2537; found: 614.2594.

2.13. Synthesis of PQ-H-PY

PQ-H (0.1 mmol, 36 mg) and **PY** (1 mmol, 169 mg) were dissolved in MeCN (20 mL) with 0.5 eq of **DiIBodipy** addition. Then, the mixture was stirred and irradiated using LED lamp (520 nm, 3W, λ_{max} = 525.1 nm, FWHM 30.8 nm) at a fixed distance for 1 cm under N_2 atmosphere. The reaction was monitored by TLC. After completion, the volatiles were evaporated and the resulting residue was purified by column chromatography on silica gel

(dichloromethane/ethyl acetate = 1:4, v/v) to afford **PQ-H-PY** as a colorless powder (34 mg, 0.065 mmol, 65 % yield). ^{1}H NMR (400 MHz, DMSO-d_6) δ 8.92 (t, *J* = 9.2 Hz, 2H), 8.34 (d, *J* = 13.3 Hz, 2H), 7.96 (d, *J* = 10.2 Hz, 2H), 7.85 (t, *J* = 8.5 Hz, 4H), 7.56 (t, *J* = 7.6 Hz, 4H), 7.45 (t, *J* = 7.3 Hz, 2H), 5.74 (s, 1H), 5.30 – 5.19 (m, 1H), 3.64 – 3.54 (m, 1H), 3.50 – 3.40 (m, 1H), 2.12 – 1.95 (m, 2H), 1.53 (s, 9H). ^{13}C NMR (151 MHz, DMSO-d_6) δ 153.7, 141.5, 140.4, 135.3, 130.9, 130.2, 130.1, 128.1, 127.1, 126.9, 126.5, 126.1, 125.7, 125.4, 125.3, 123.4, 120.7, 28.4, 20.7. HR-MS (ESI) *m/z*, calculated for $[M+H]^+$: 530.2326; found: 530.2264.

2.14. Synthesis of PQ-^{t}Bu-PY

The title compound was synthesized using the same method as for **PQ-H-PY**, employing **PQ-*t*Bu** (0.1 mmol, 47 mg) to afford **PQ-^{t}Bu-PY** as a colorless powder (48 mg, 0.075 mmol, 75 % yield). ^{1}H NMR (400 MHz, DMSO-d_6) δ 8.86 (d, *J* = 8.8 Hz, 2H), 8.37 – 8.28 (m, 2H), 7.97 – 7.89 (m, 2H), 7.78 (d, *J* = 8.3 Hz, 4H), 7.57 (d, *J* = 8.4 Hz, 4H), 5.76 (s, 1H), 5.29 – 5.19 (m, 1H), 3.64 – 3.50 (m, 2H), 2.05 – 1.94 (m, 2H), 1.54 (s, 9H), 1.36 (s, 18H). ^{13}C NMR (151 MHz, *$CDCl_3$)* δ 150.4, 139.0, 137.9, 126.9, 126.7, 126.4, 126.1, 125.7, 124.2, 123.0, 118.6, 80.9, 78.9, 53.4, 40.1, 39.9, 39.8, 34.5, 31.3, 28.4. HR-MS (ESI) *m/z*, calculated for $[M+H]^+$: 642.3578; found: 642.3532.

2.15. Synthesis of PQ-CH_3-PY

The title compound was synthesized using the same method as for **PQ-H-PY**, employing **PQ-CH_3** (0.1 mmol, 39 mg) to afford **PQ-CH_3-PY** as a colorless powder (45 mg, 0.08 mmol, 80 % yield). ^{1}H NMR (400 MHz, DMSO-d_6) δ 8.85 (d, *J* = 10.2 Hz, 2H), 8.32 (d, *J* = 15.4 Hz, 2H), 7.92 (d, *J* = 10.0 Hz, 2H), 7.74 (t, *J* = 7.6 Hz, 4H), 7.36 (d, *J* = 7.6 Hz, 4H), 5.72 (s, 1H), 5.30 – 5.18 (m, 1H), 3.60 (t, *J* = 9.8 Hz, 1H), 3.49 – 3.40 (m, 1H), 2.39 (s, 6H), 2.02 (dt, *J* = 23.2, 11.1 Hz, 2H), 1.54 (s, 9H). ^{13}C NMR (151 MHz, $CDCl_3$) δ 153.8, 139.0, 137.8, 137.1, 129.52, 127.0, 126.8, 126.4, 126.1, 125.5, 125.4, 124.1, 123.0, 118.3, 80.8, 39.9, 31.4, 29.5, 28.3, 21.0. HR-MS (ESI) *m/z*, calculated for $[M+H]^+$: 558.2639; found: 558.2579.

2.16. Synthesis of PQ-OCH_3-PY

The title compound was synthesized using the same method as for **PQ-H-PY**, employing **PQ-OCH_3** (0.1 mmol, 42 mg) to afford **PQ-OCH_3-PY** as a colorless powder (42 mg, 0.072 mmol, 72 % yield). ^{1}H NMR (400 MHz, DMSO-d_6) δ 8.83 (d, J = 9.0 Hz, 2H), 8.28 (d, J = 13.5 Hz, 2H), 7.90 (dd, J = 8.5, 1.9 Hz, 2H), 7.83 – 7.72 (m, 4H), 7.11 (d, J = 10.3 Hz, 4H), 5.73 (s, 1H), 5.28 – 5.19 (m, 1H), 3.84 (s, 6H), 3.60 (s, 1H), 3.43 (s, 1H), 2.04 – 1.97 (m, 2H), 1.54 (s, 9H). ^{13}C NMR (101 MHz, DMSO-d_6) δ 153.7, 143.9, 143.8, 131.4, 131.3, 129.1, 129.0, 127.0, 126.9, 126.4, 126.3, 125.7, 125.2, 125.1, 125.0, 121.8, 119.7, 80.6, 28.5. HR-MS (ESI) *m/z*, calculated for $[M+Na]^+$: 589.2459; found: 589.2439.

3. NMR Spectra

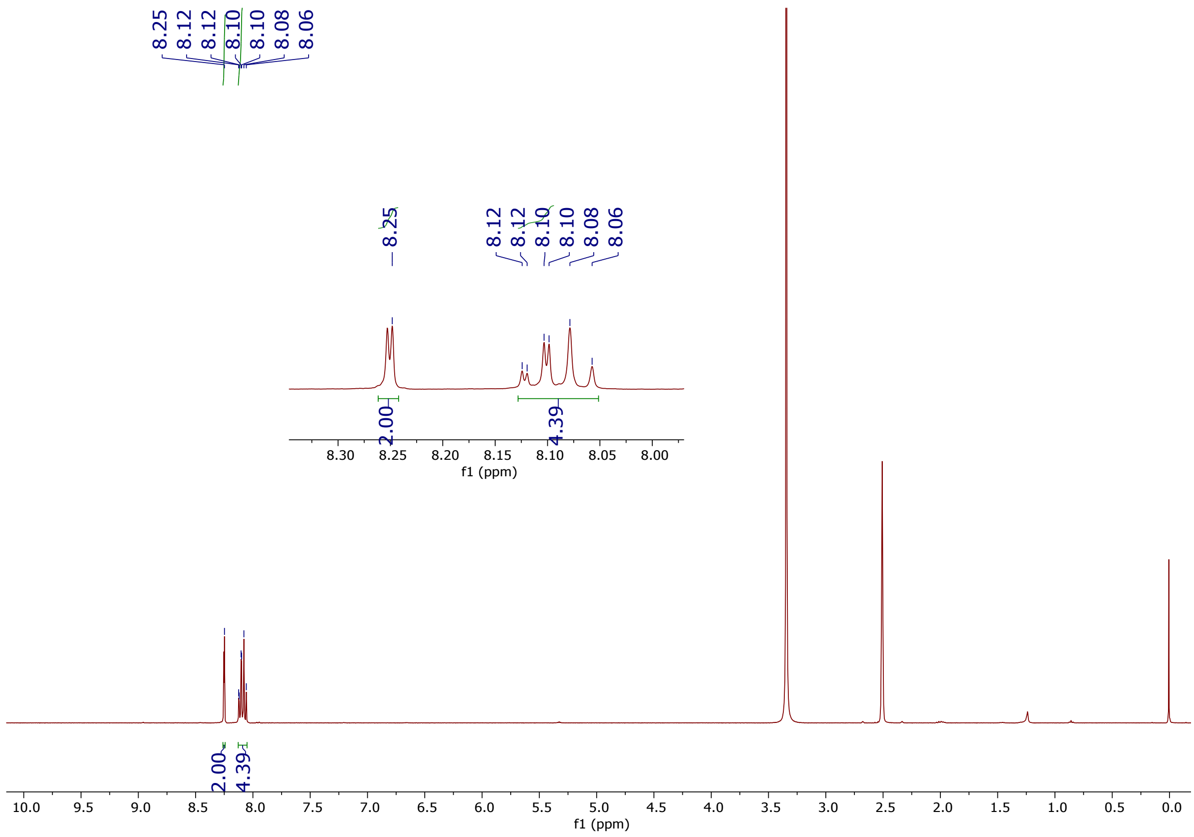

Fig. S6. ^{1}H NMR of **PQ-2DiI** in DMSO-d_6.

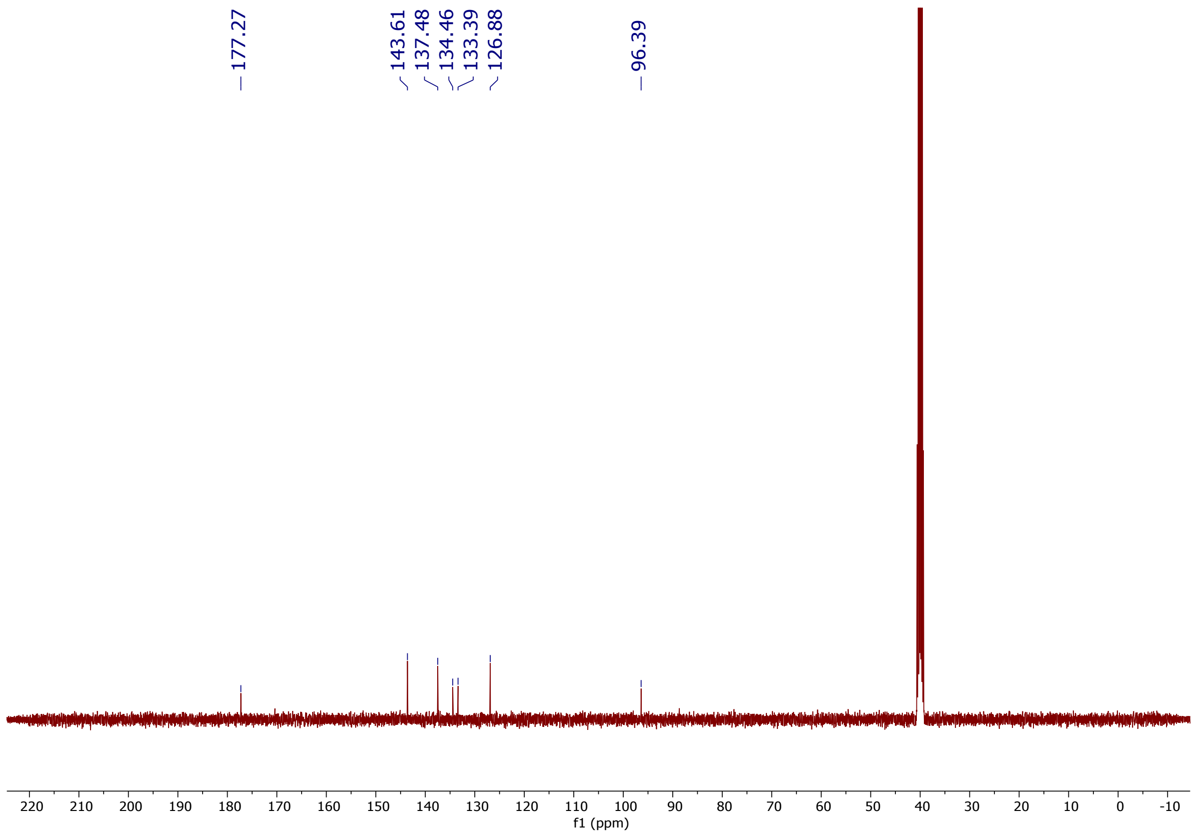

Fig. S7. ^{13}C NMR of **PQ-2DiI** in DMSO-d_6.

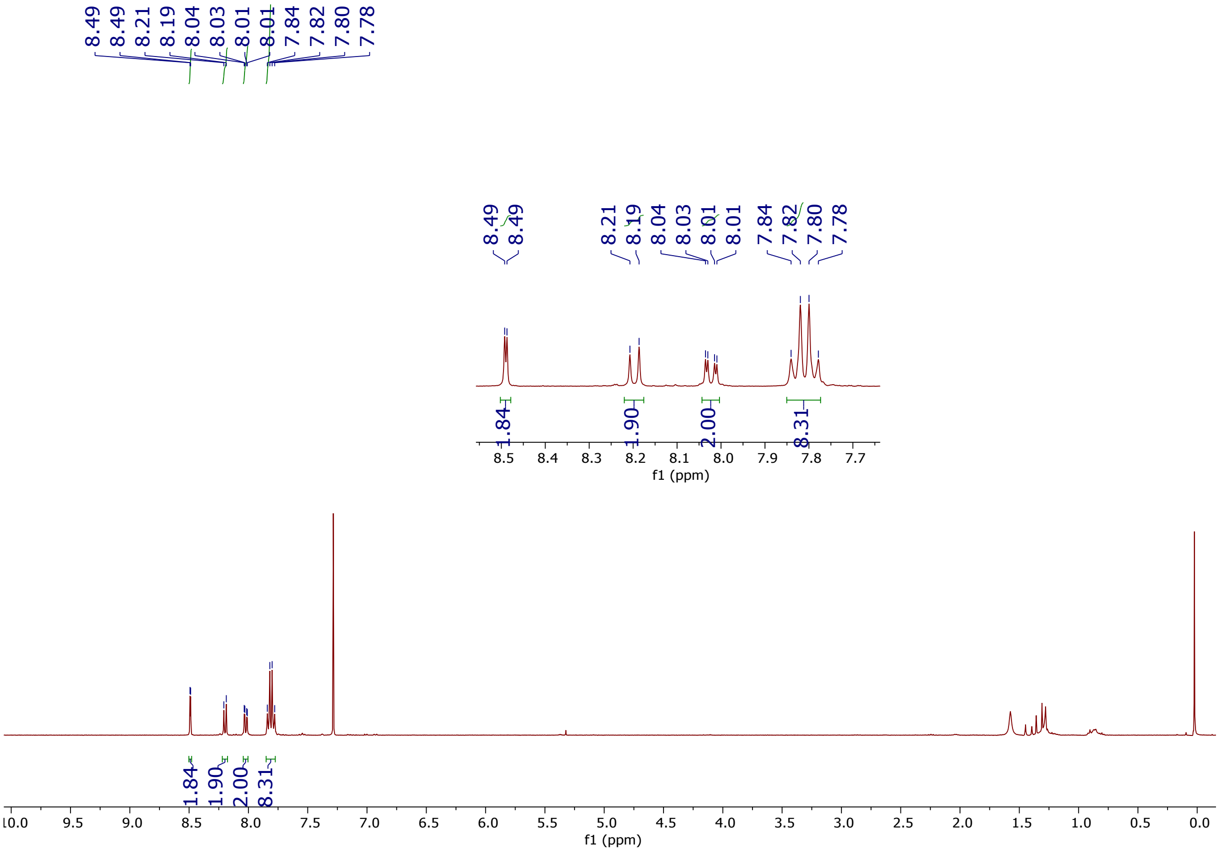

Fig. S8. ^{13}H NMR of **PQ-CF$_3$** in *CDCl$_3$*.

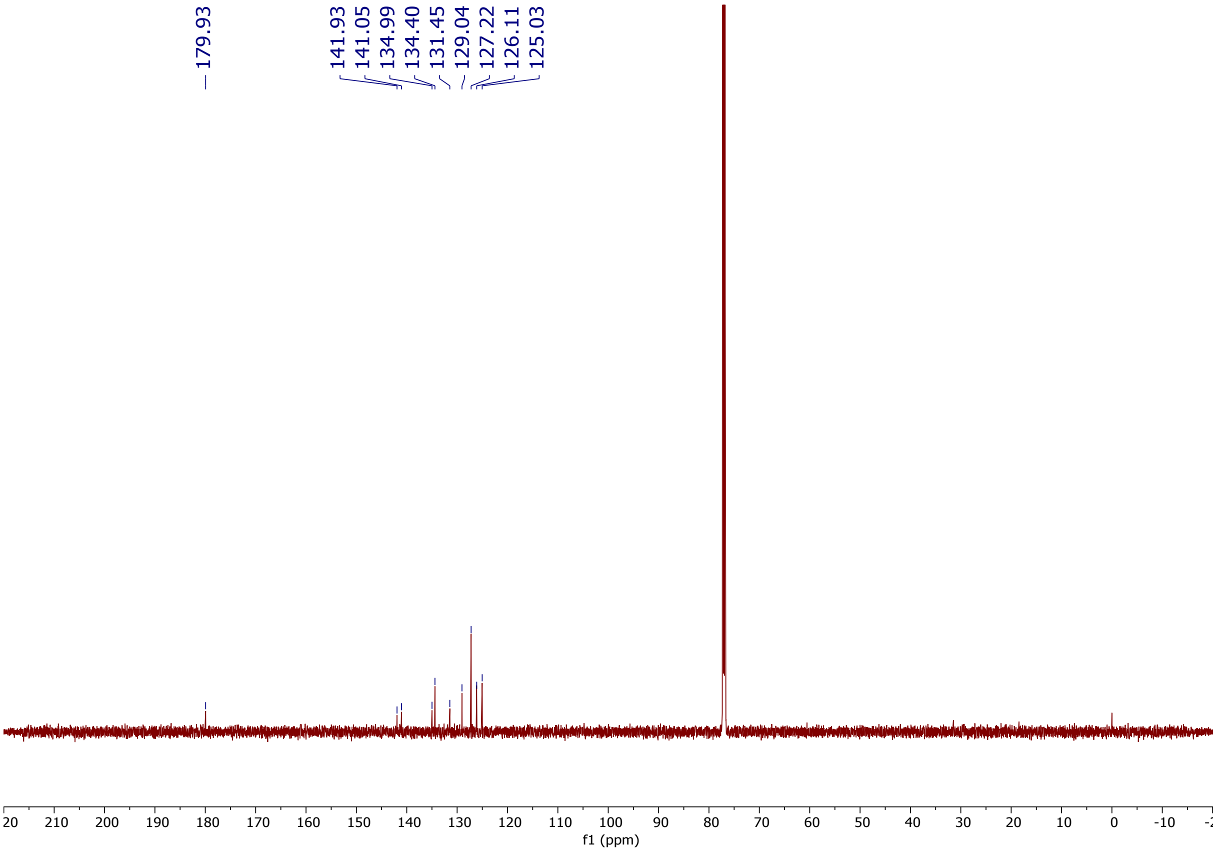

Fig. S9. ^{13}C NMR of **PQ-CF$_3$** in *CDCl$_3$*.

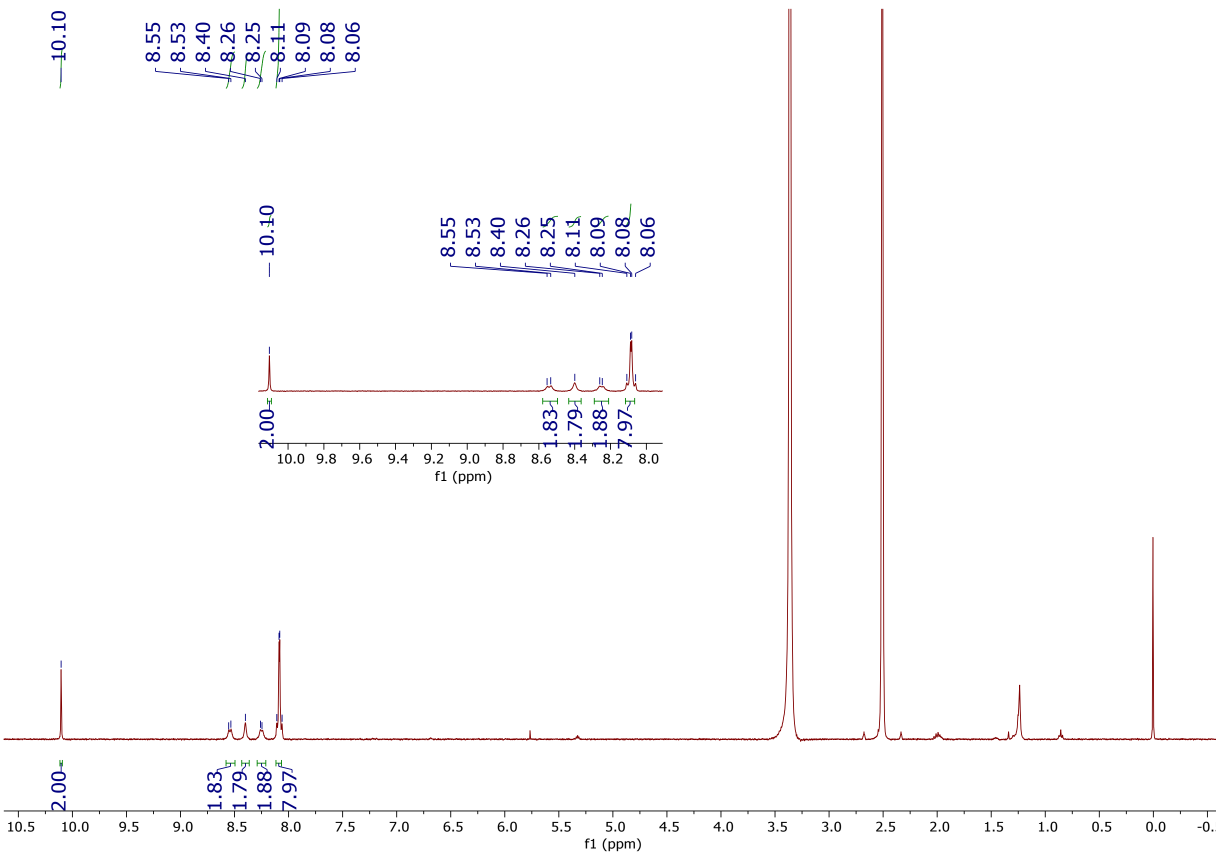

Fig. S10. ^{1}H NMR of **PQ-CHO** in DMSO-d_6.

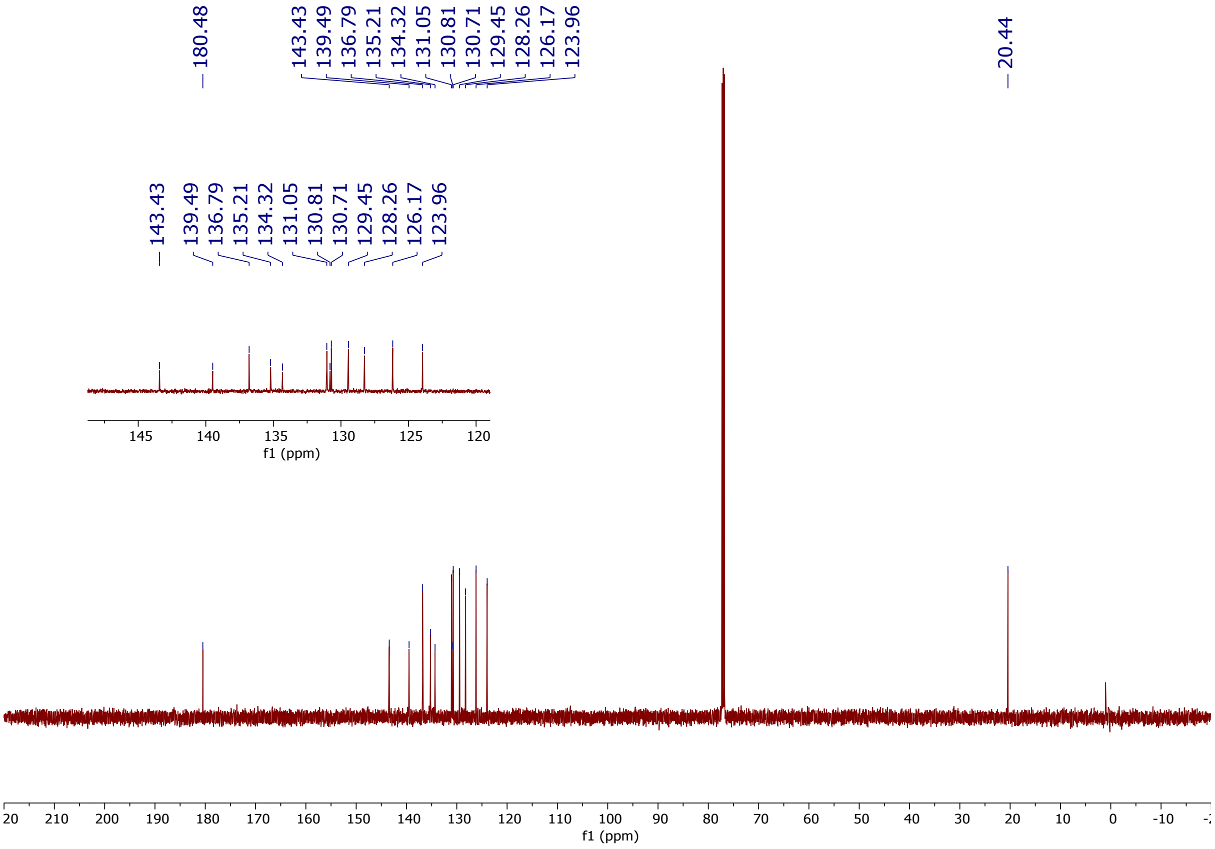

Fig. S11. ^{13}C NMR of **PQ-CHO** in $CDCl_3$.

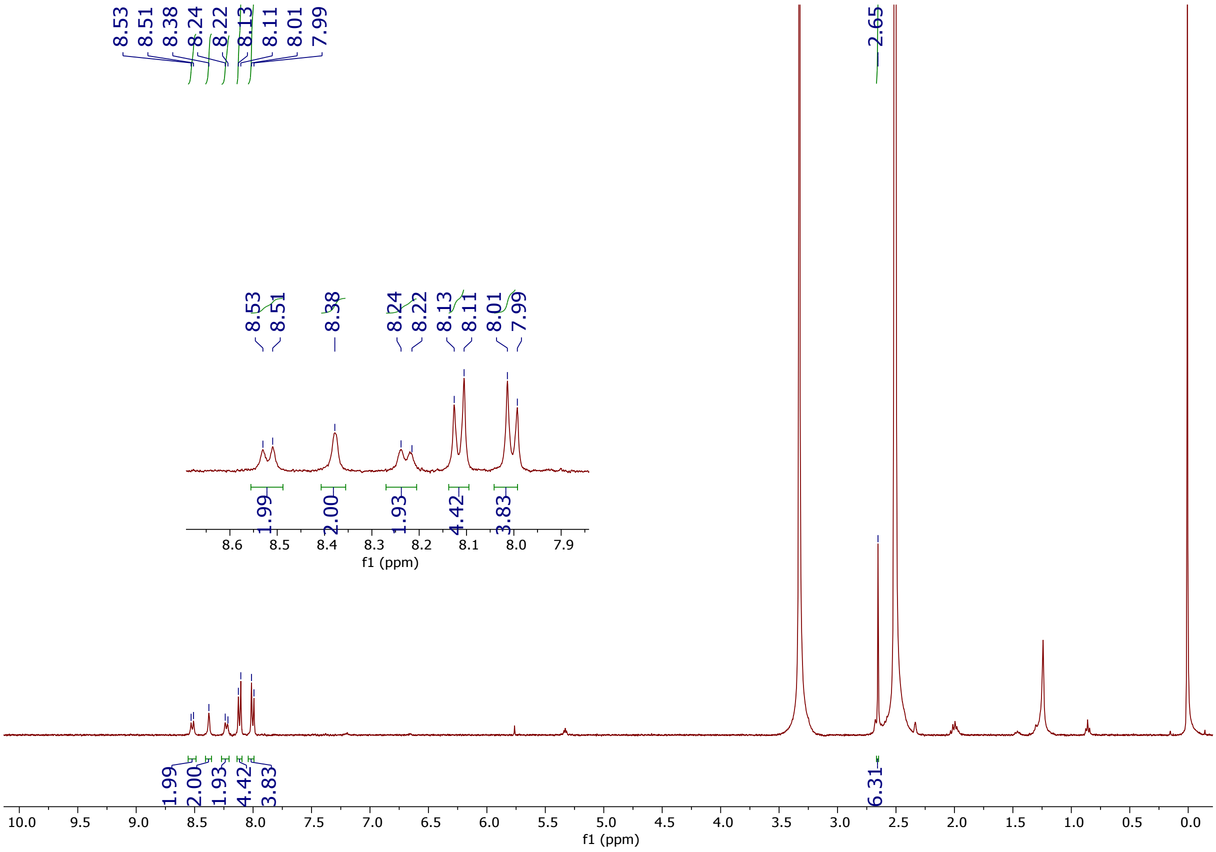

Fig. S12. [13]H NMR of **PQ-COCH$_3$** in *CDCl$_3$*.

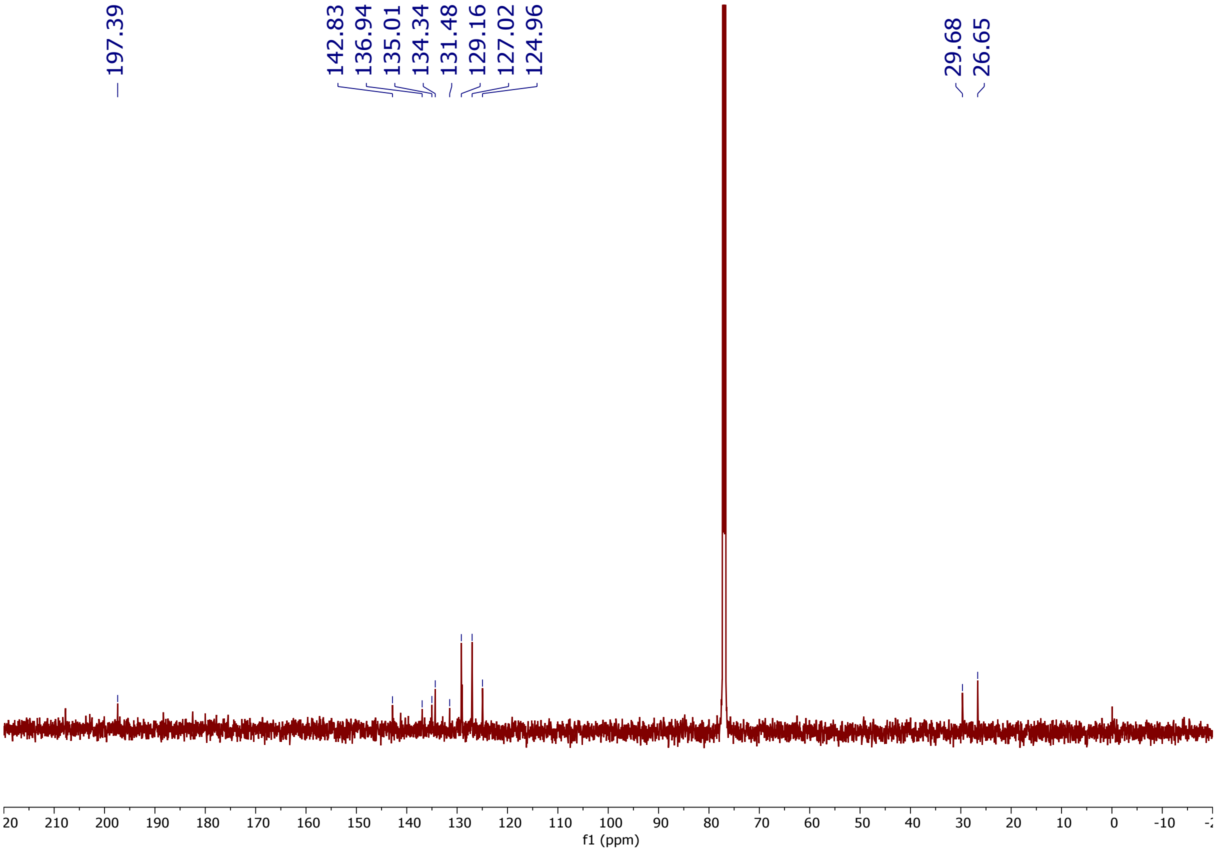

Fig. S13. ^{13}C NMR of **PQ-COCH$_3$** in *CDCl$_3$*.

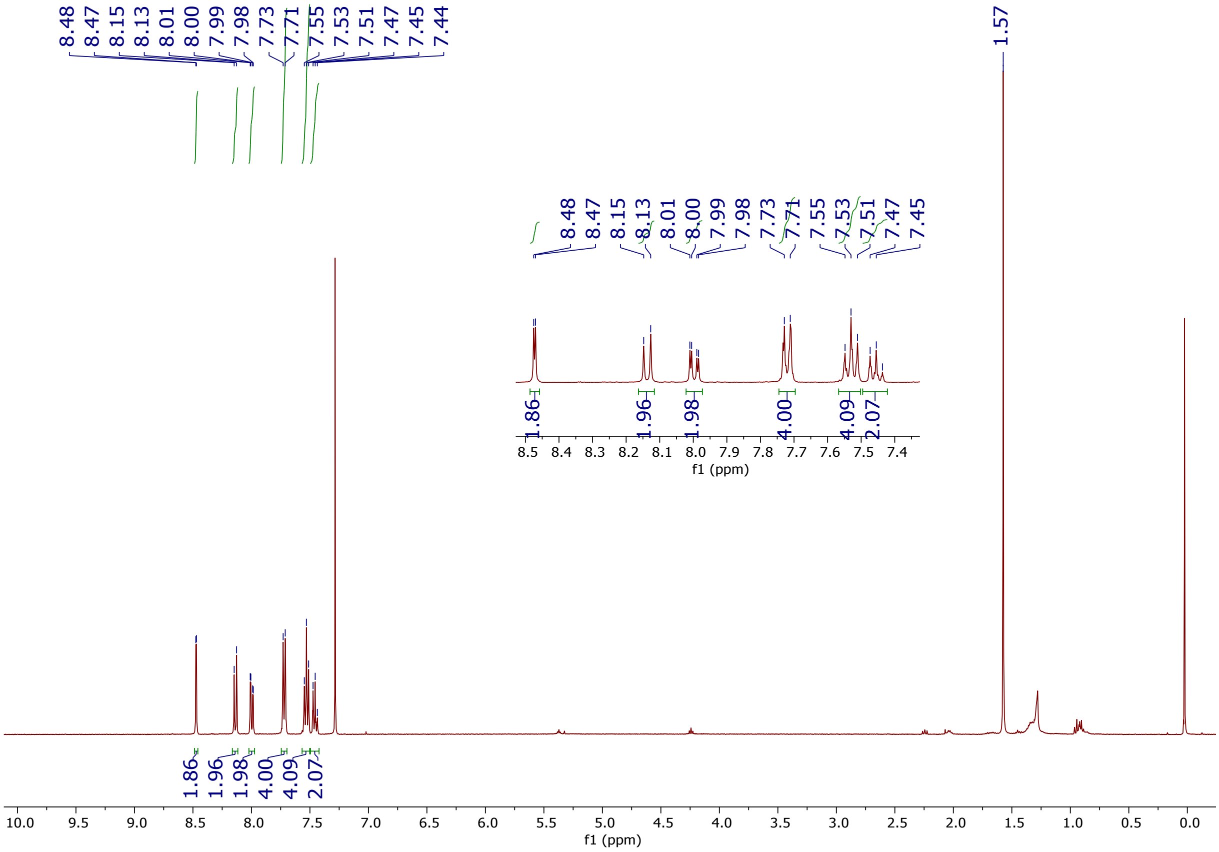

Fig. S14. ^{1}H NMR of **PQ-H** in *$CDCl_3$*.

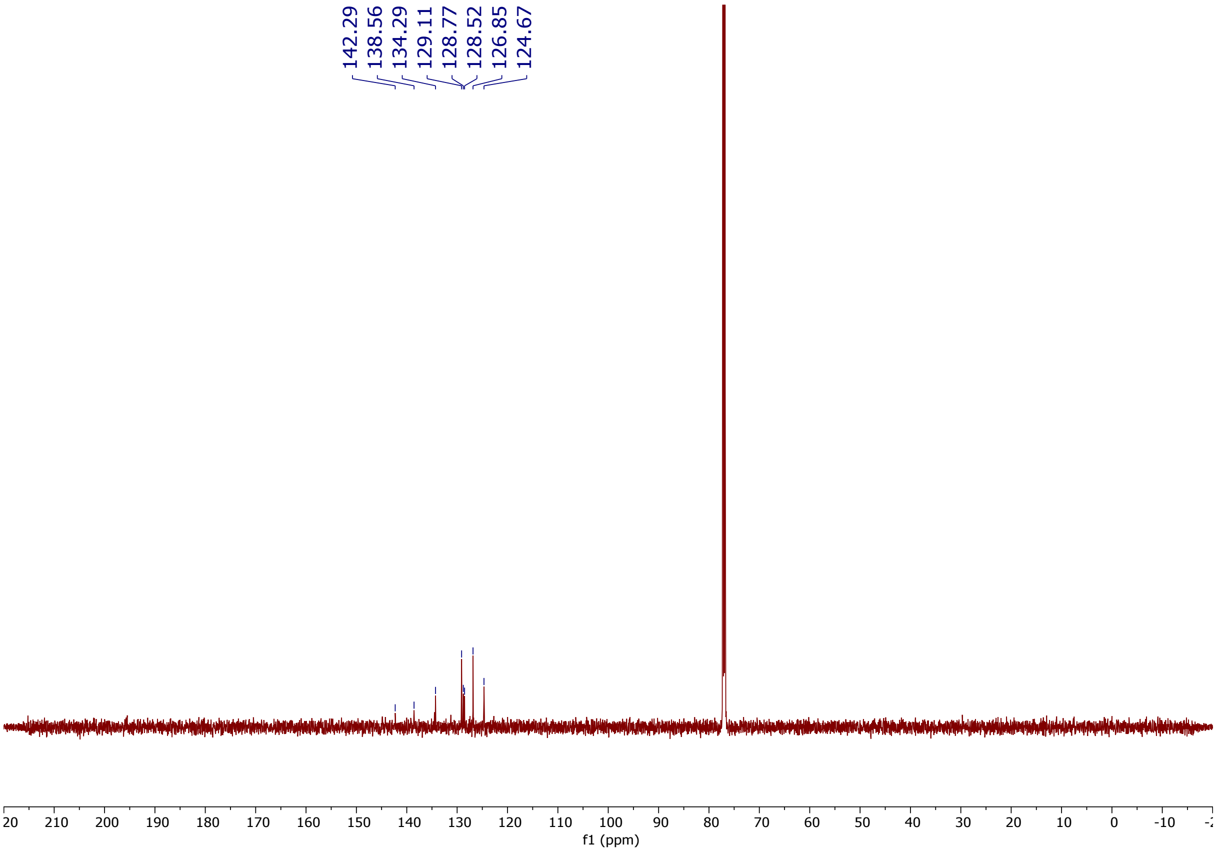

Fig. S15. ^{13}C NMR of **PQ-H** in *$CDCl_3$*.

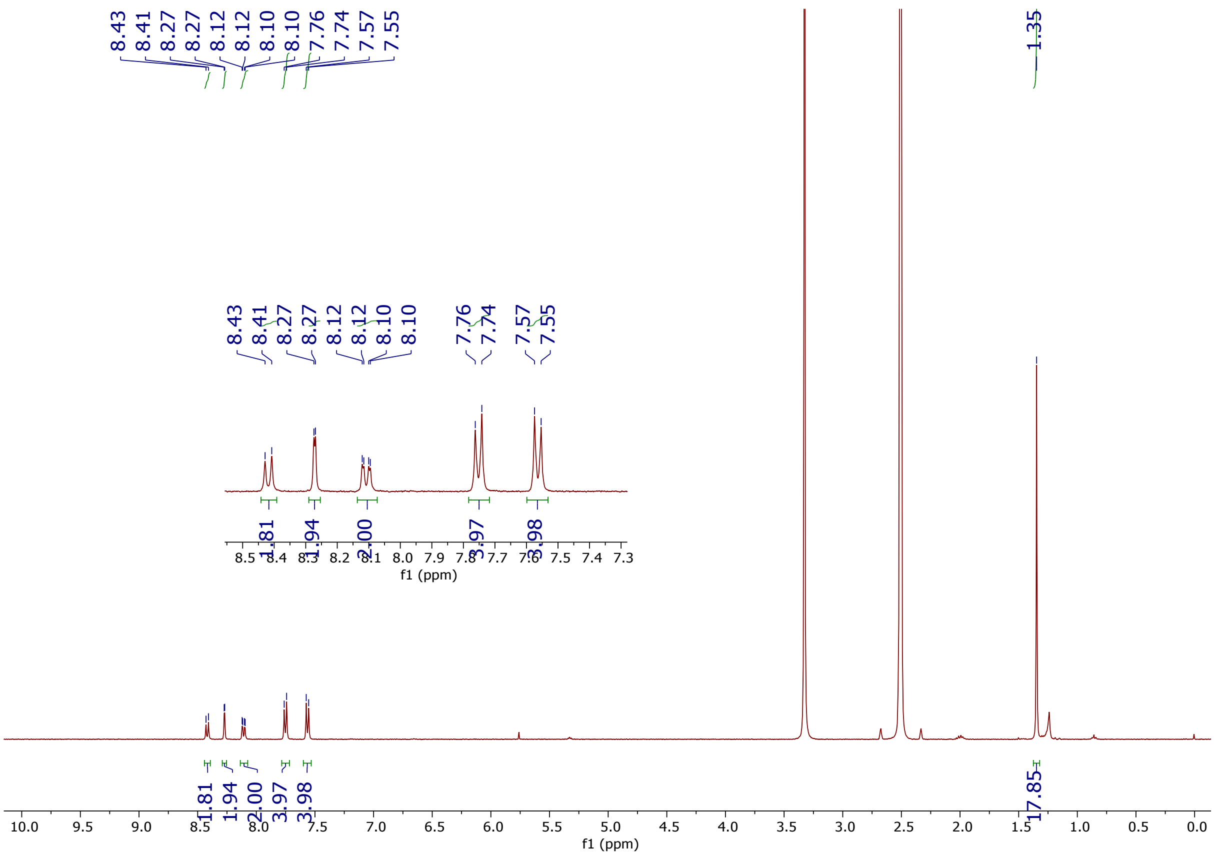

Fig. S16. [13]H NMR of **PQ-^{t}Bu** in DMSO-d_6.

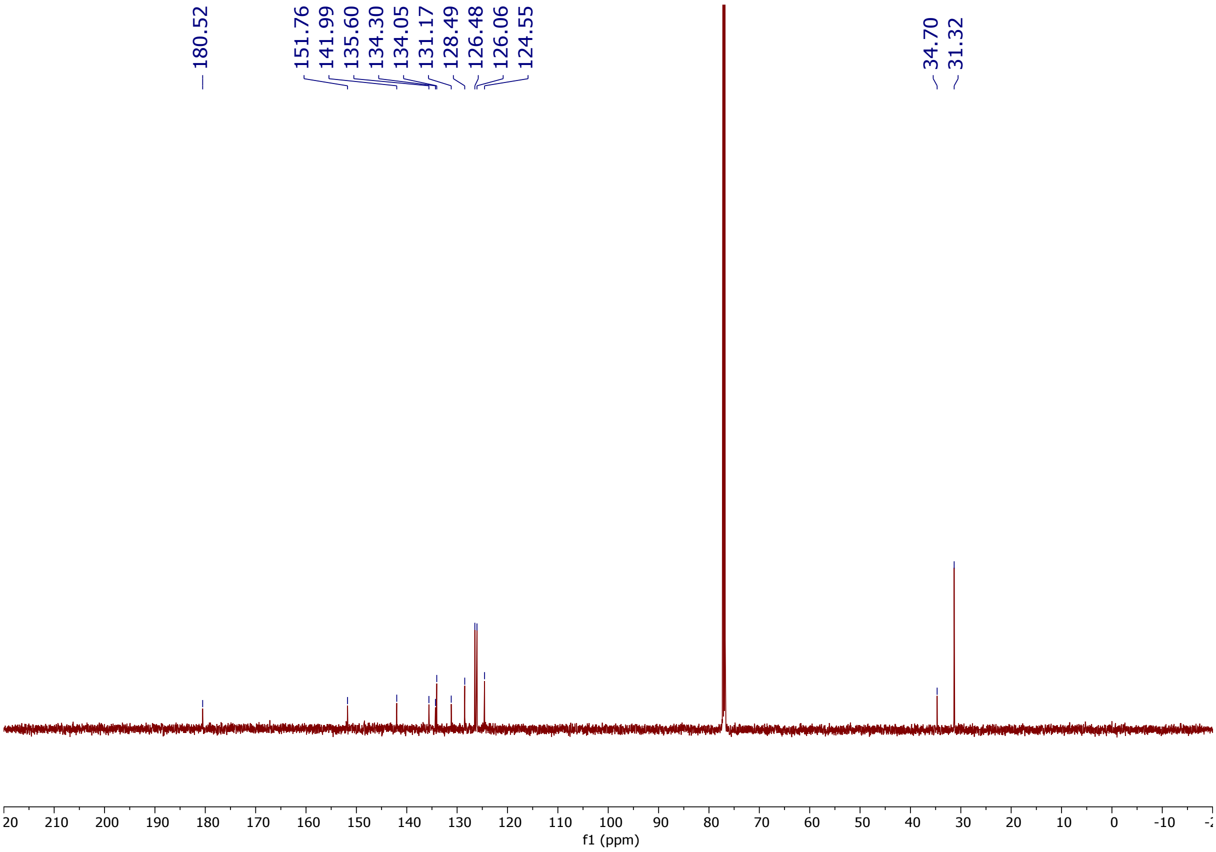

Fig. S17. ^{13}C NMR of **PQ-^{t}Bu** in *CDCl$_3$*.

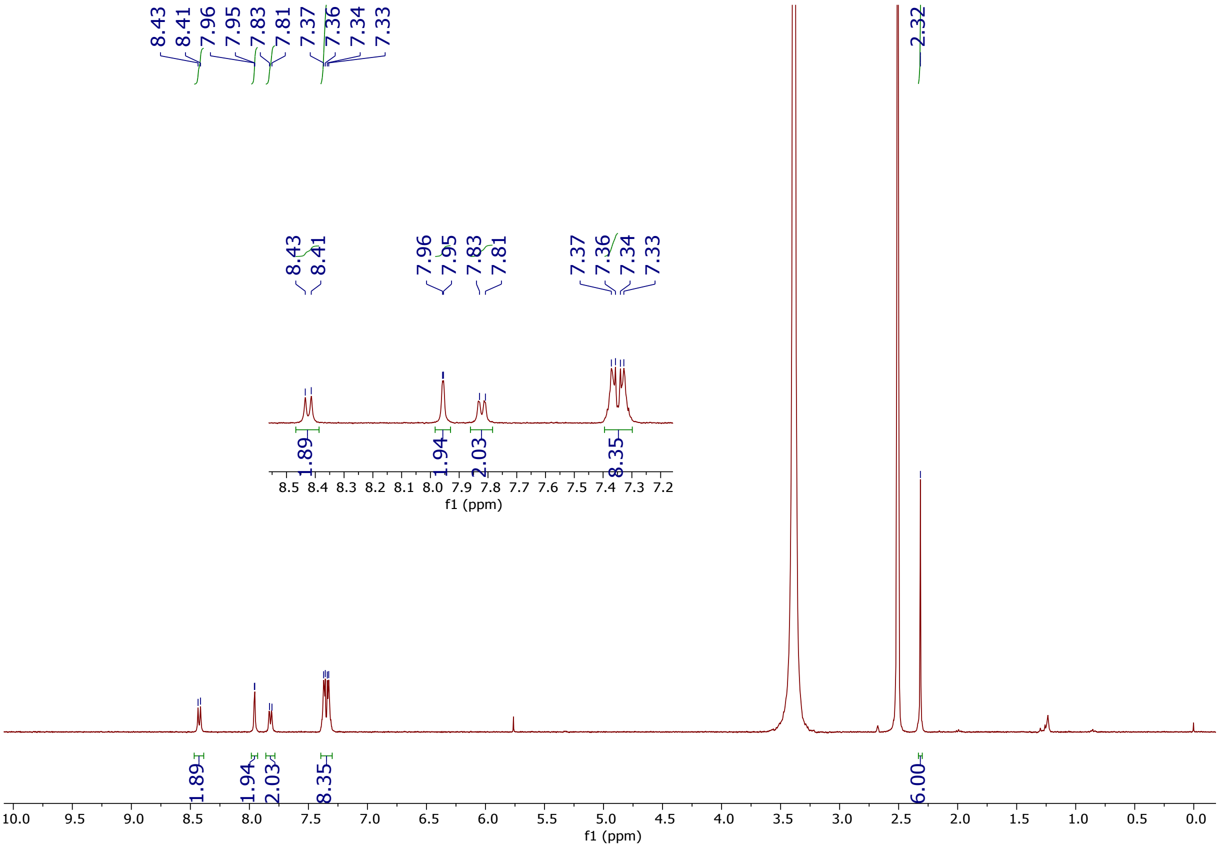

Fig. S18. ^{1}H NMR of **PQ-CH$_3$** in DMSO-d_6.

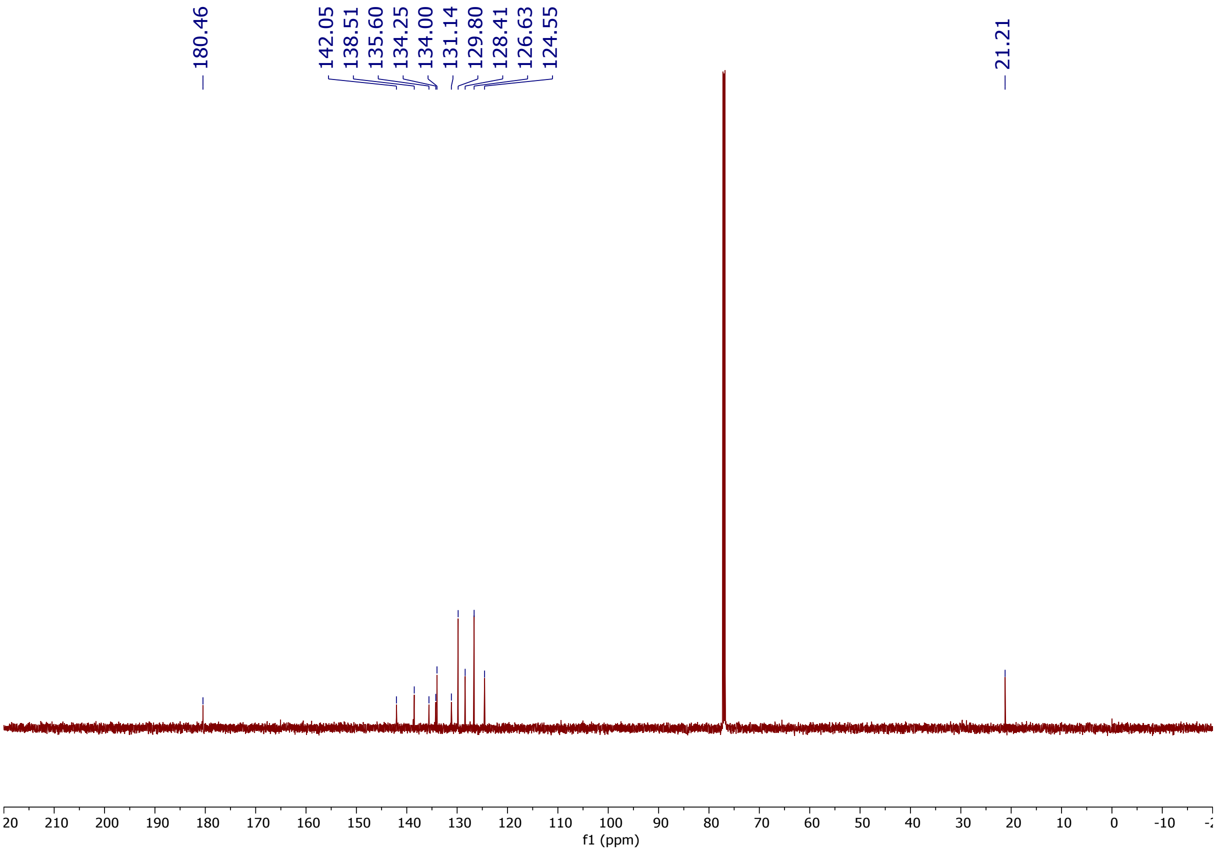

Fig. S19. ^{13}C NMR of **PQ-CH$_3$** in DMSO-d_6.

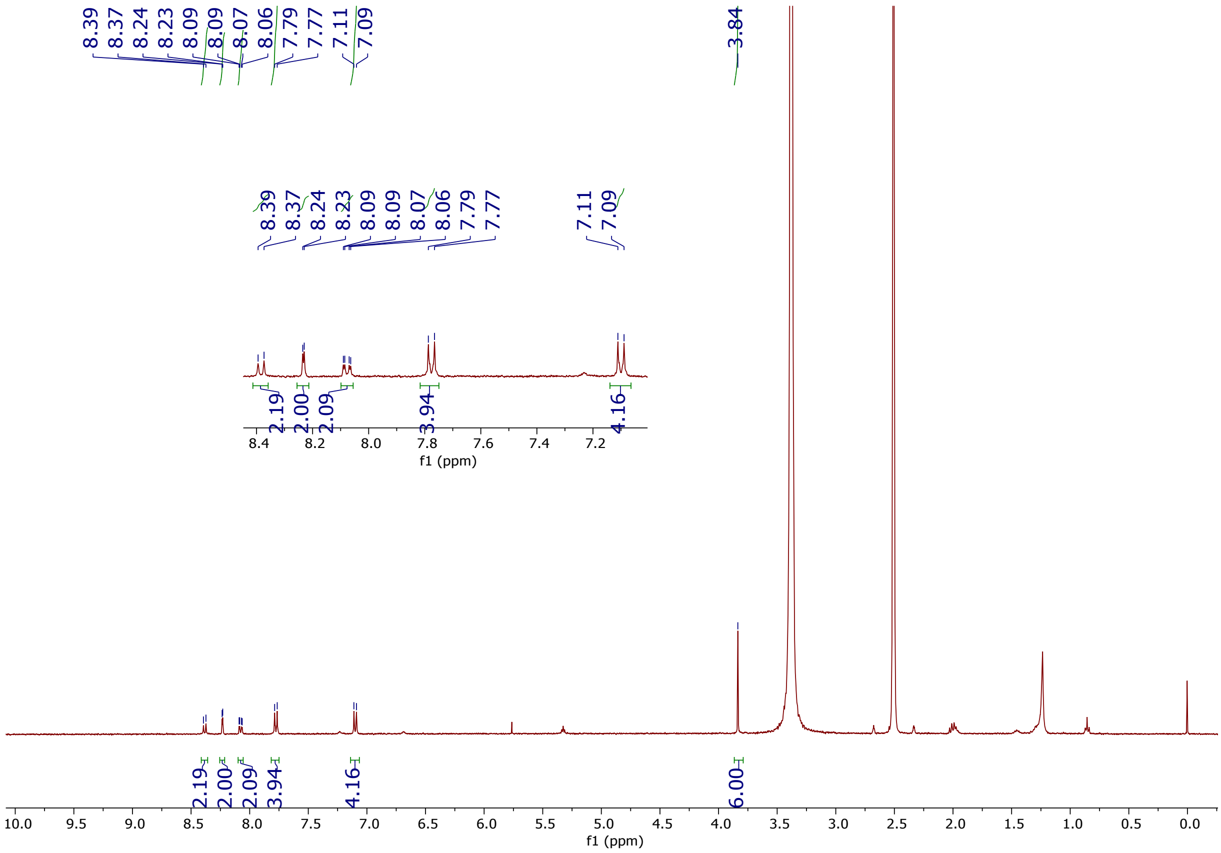

Fig. S20. ^{13}H NMR of **PQ-OCH$_3$** in DMSO-d_6.

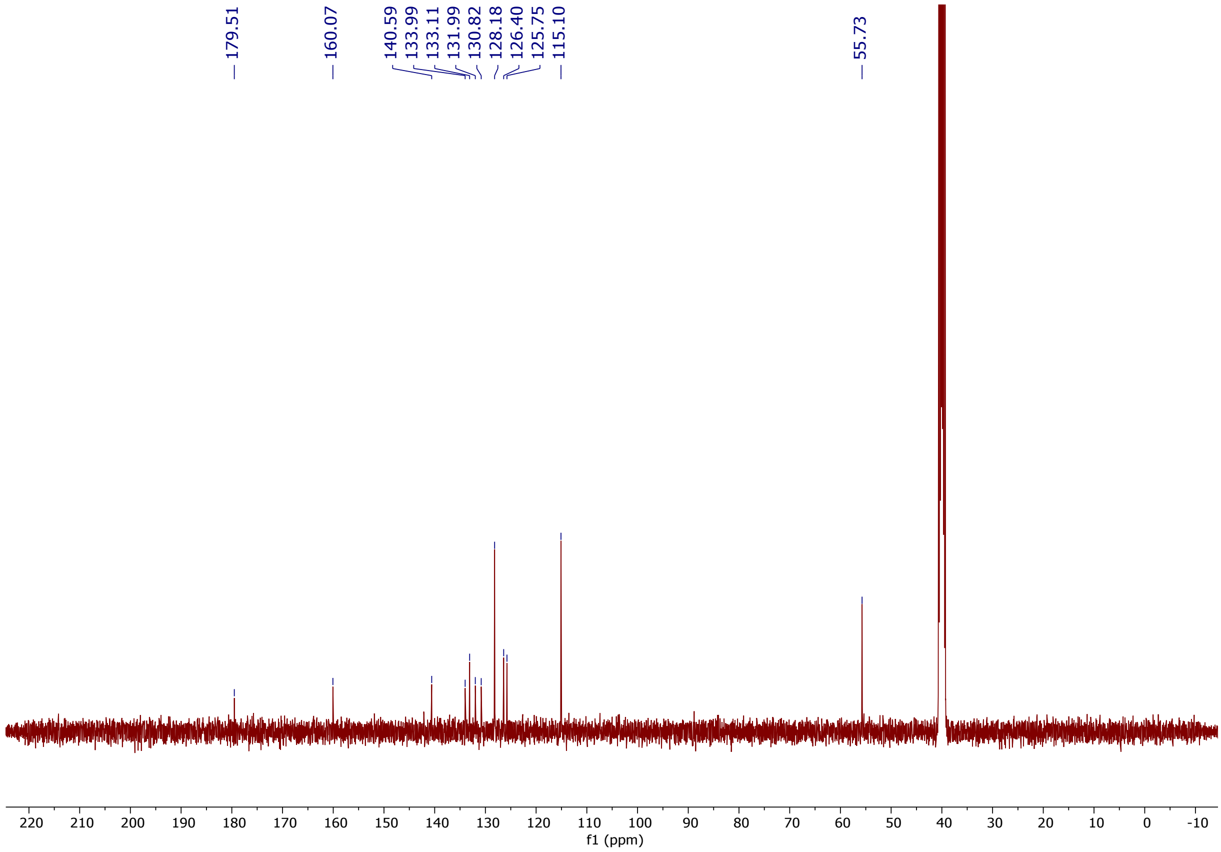

Fig. S21. ^{13}C NMR of **PQ-OCH$_3$** in DMSO-d_6.

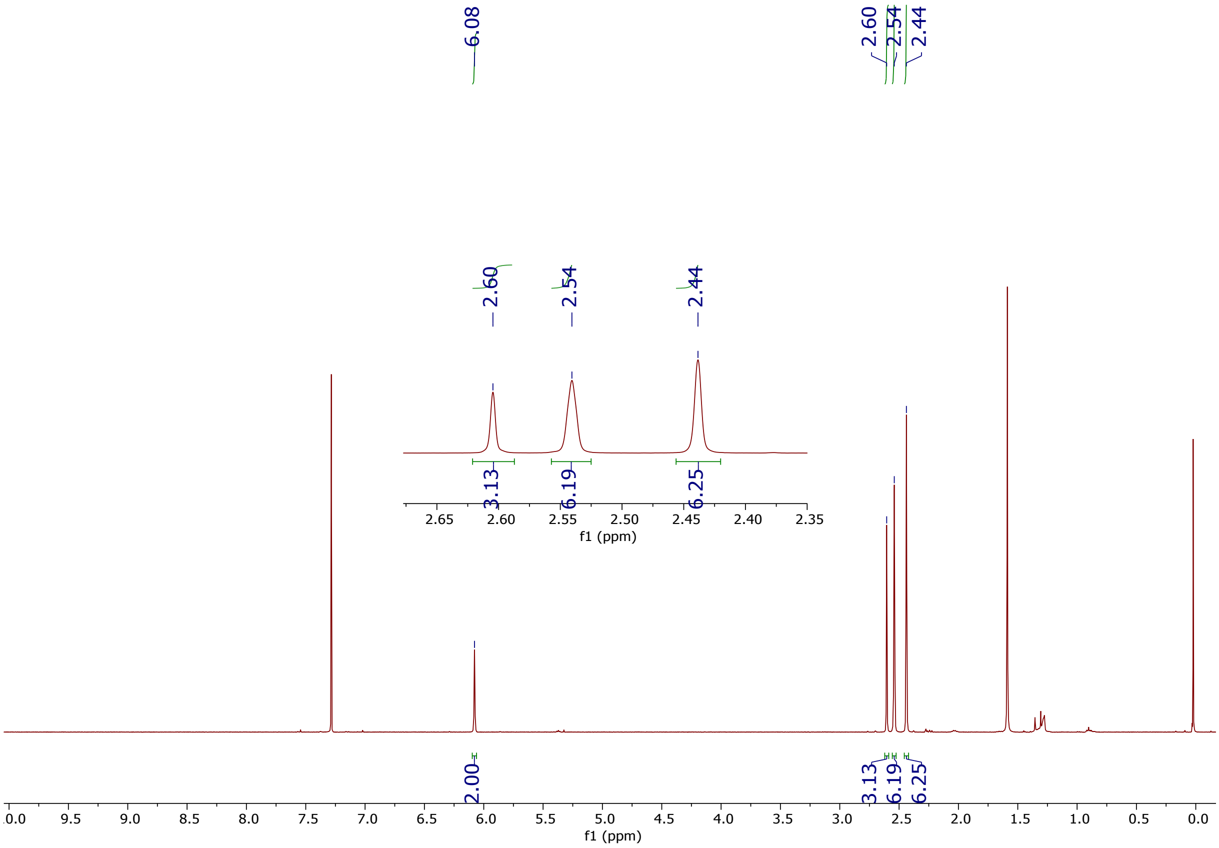

Fig. S22. ^{1}H NMR of **Me-Bodipy** in *CDCl$_3$*.

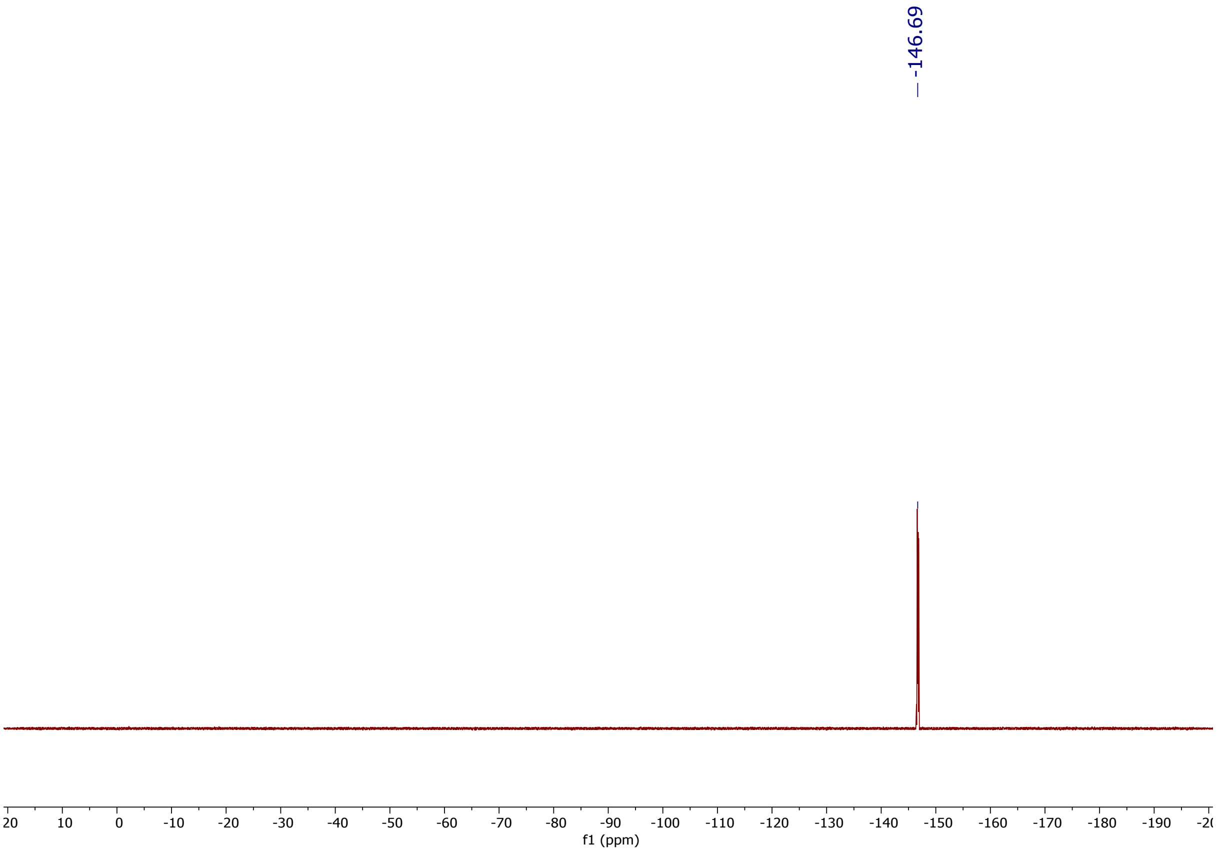

Fig. S23. ^{19}F NMR of **Me-Bodipy** in *CDCl$_3$*.

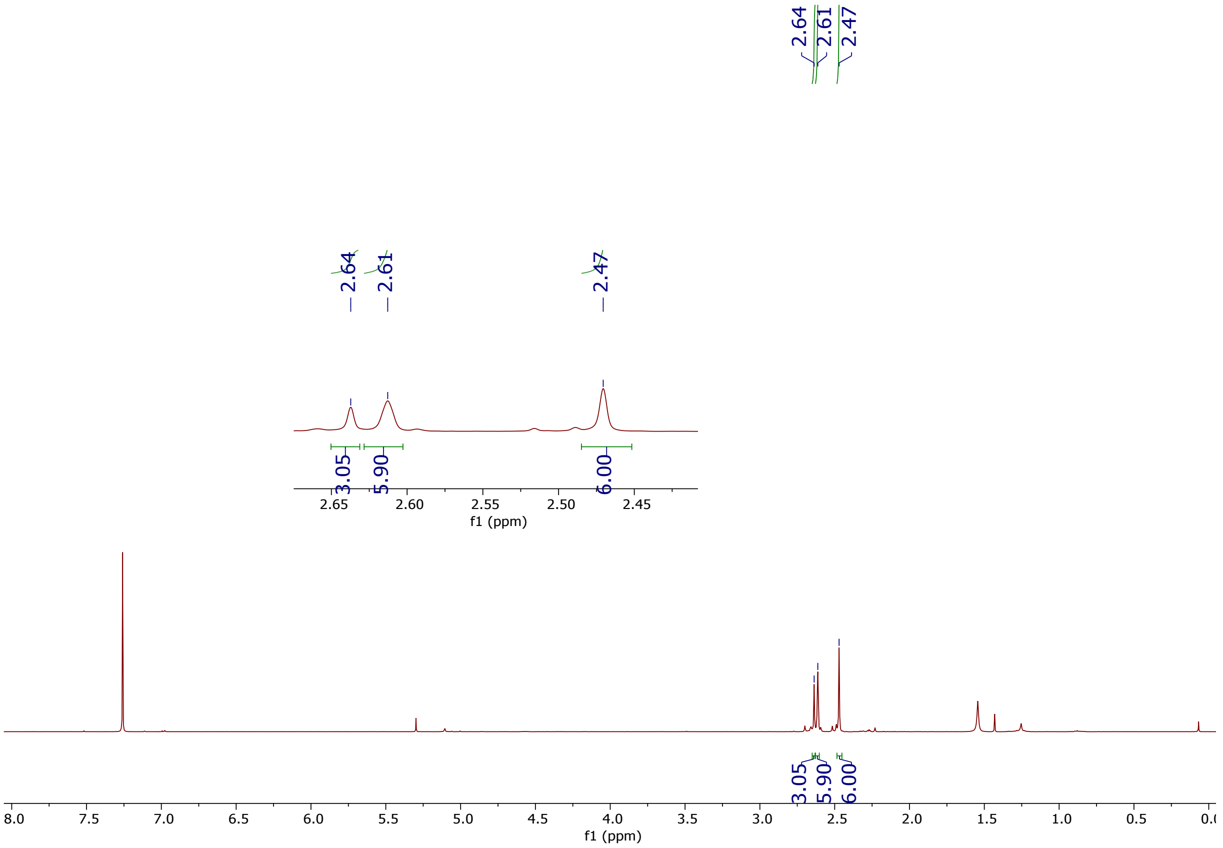

Fig. S24. 13H NMR of **DiIbodipy** in *CDCl₃*.

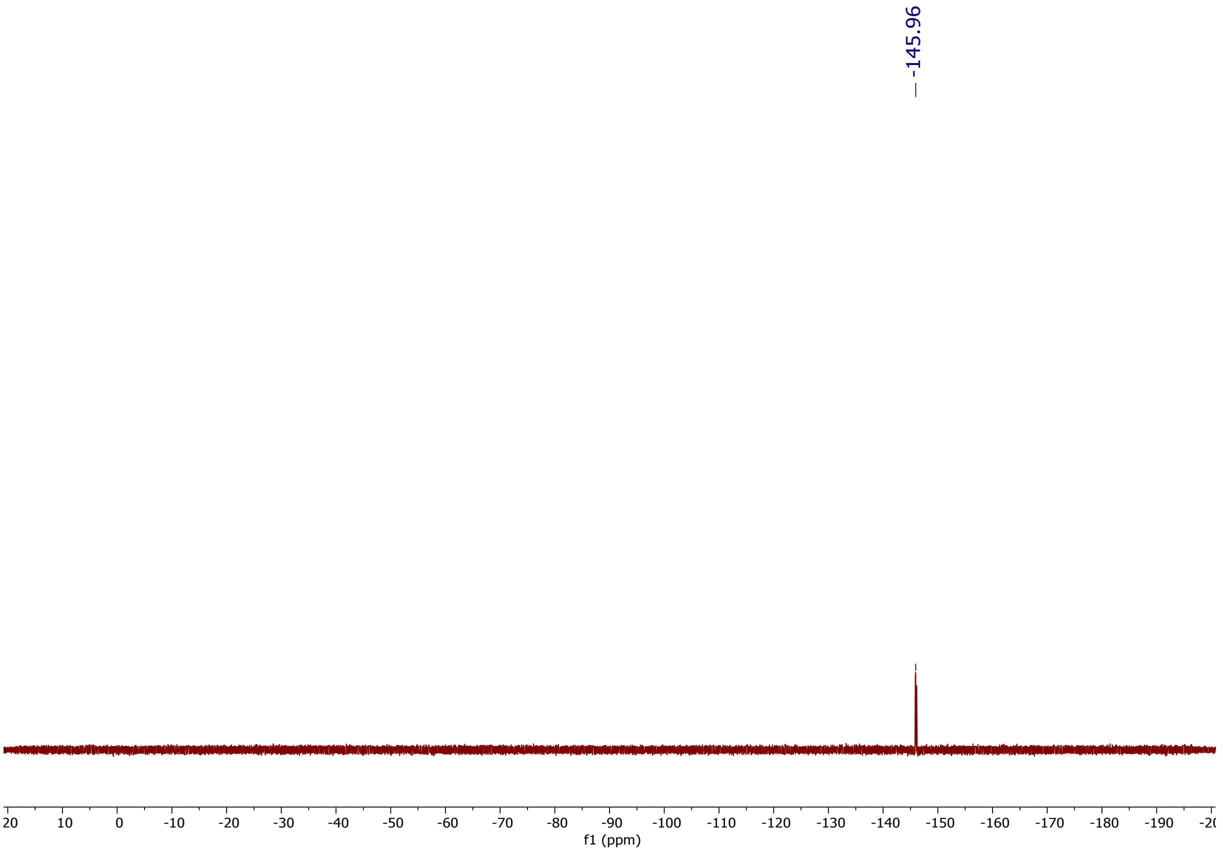

Fig. S25. 19F NMR of **DiIbodipy** in *CDCl₃*.

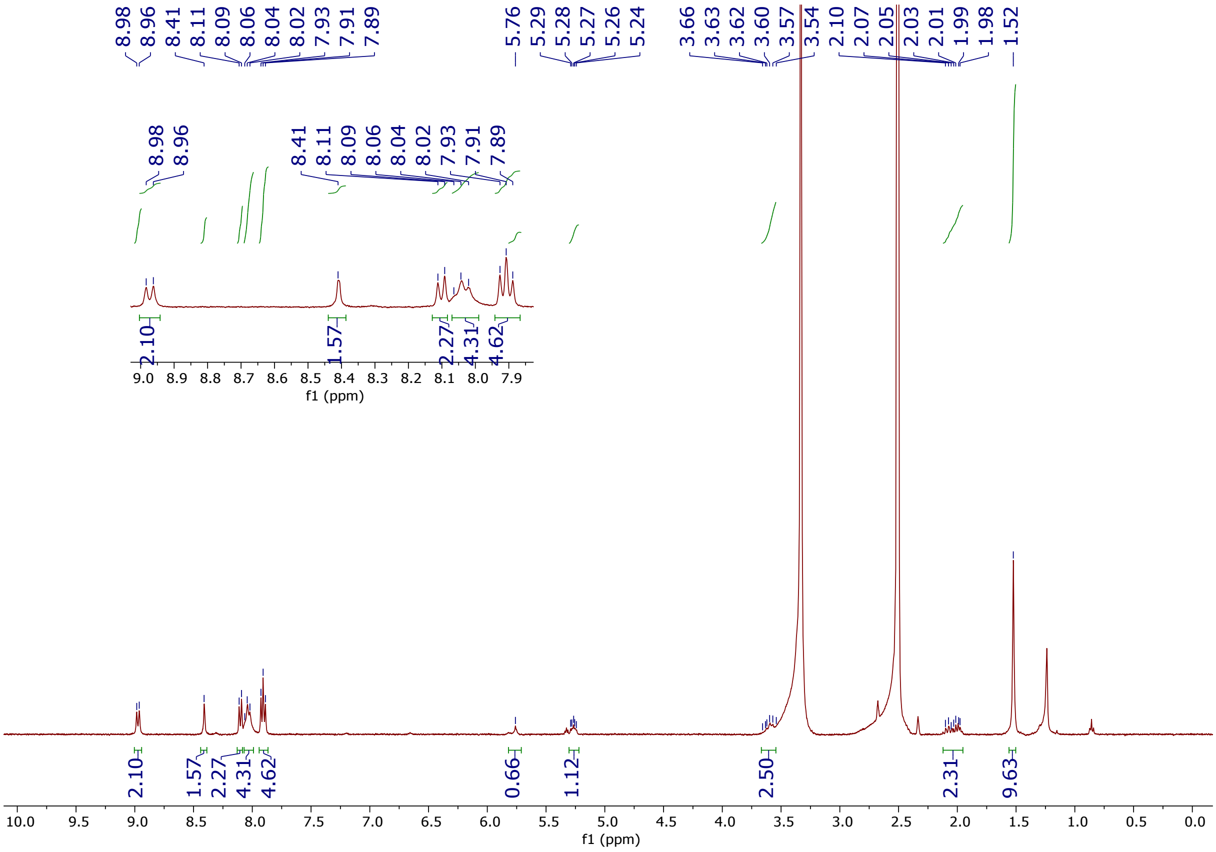

Fig. S26. ^{1}H NMR of **PQ-CF_3-PY** in DMSO-d_6.

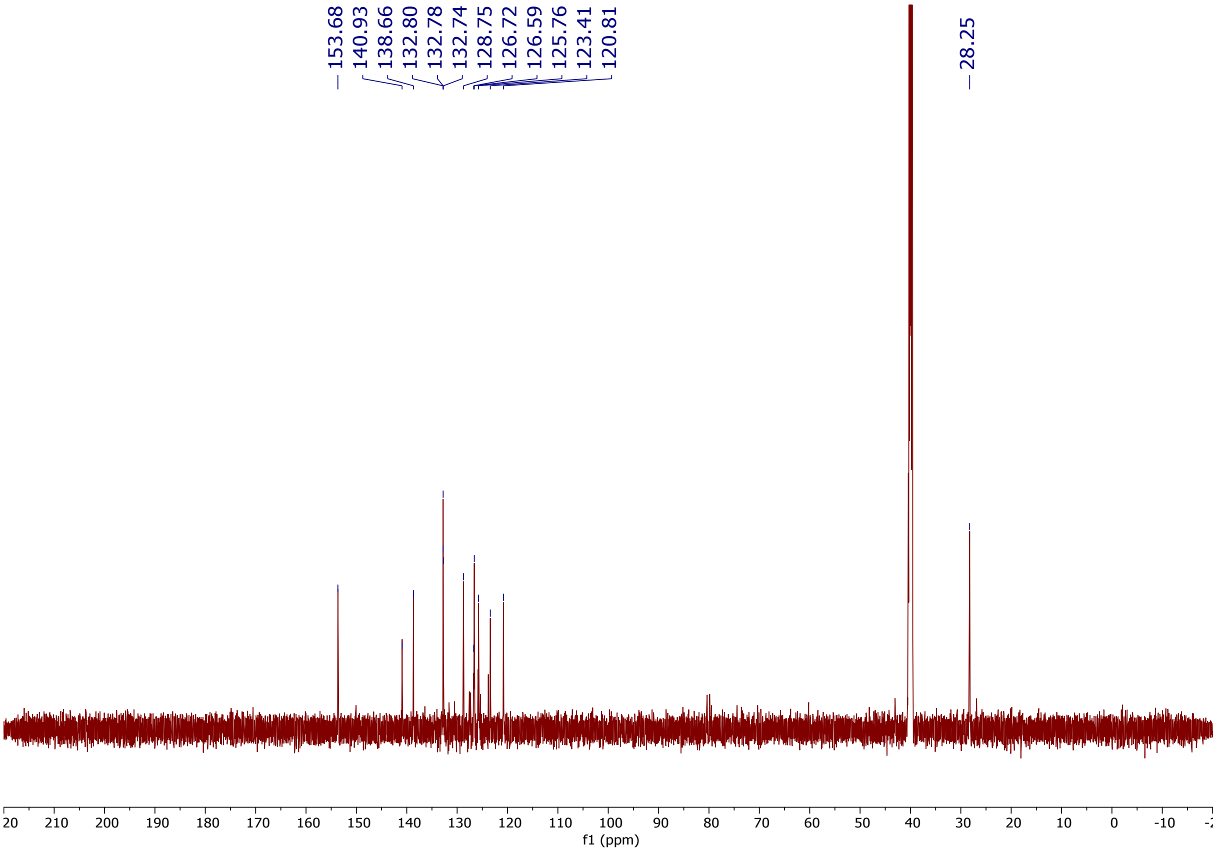

Fig. S27. ^{13}C NMR of **PQ-CF_3-PY** in DMSO-d_6.

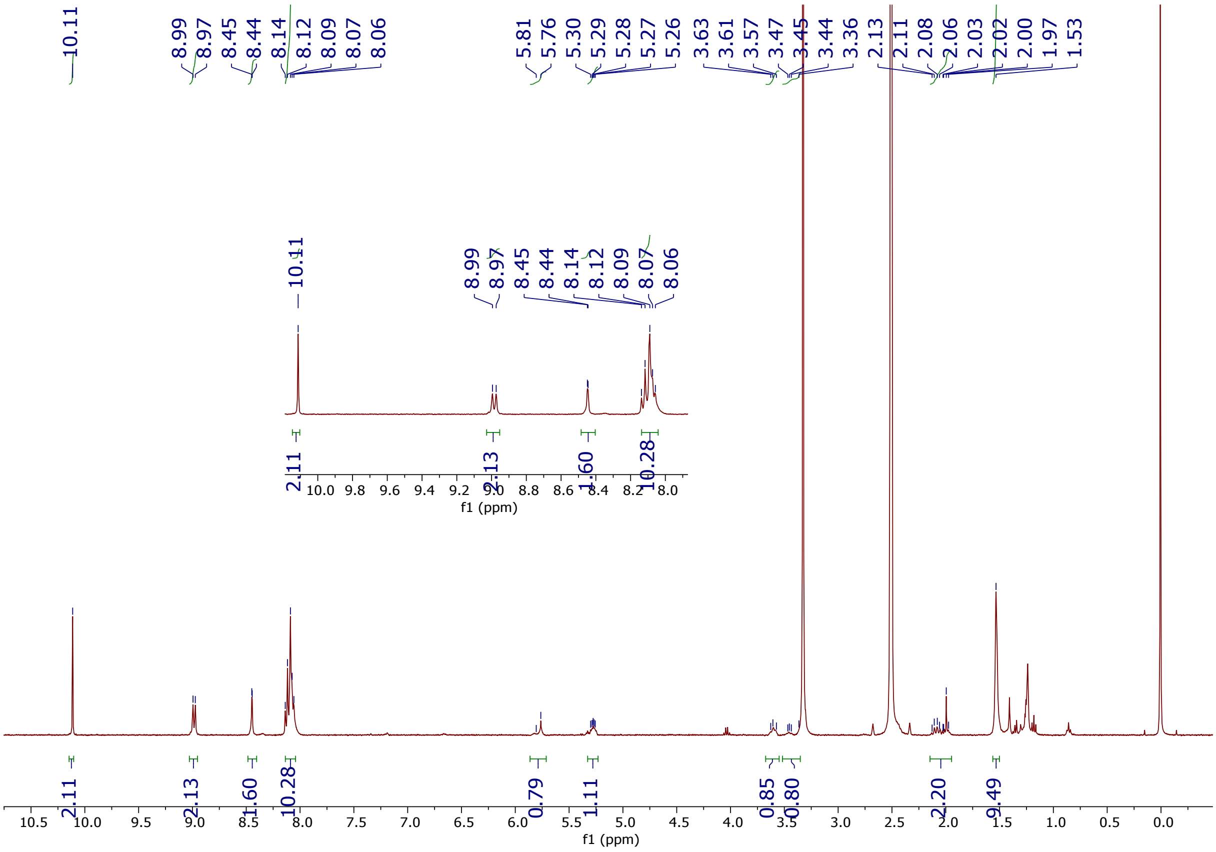

Fig. S28. ^{13}H NMR of **PQ-CHO-PY** in DMSO-d_6.

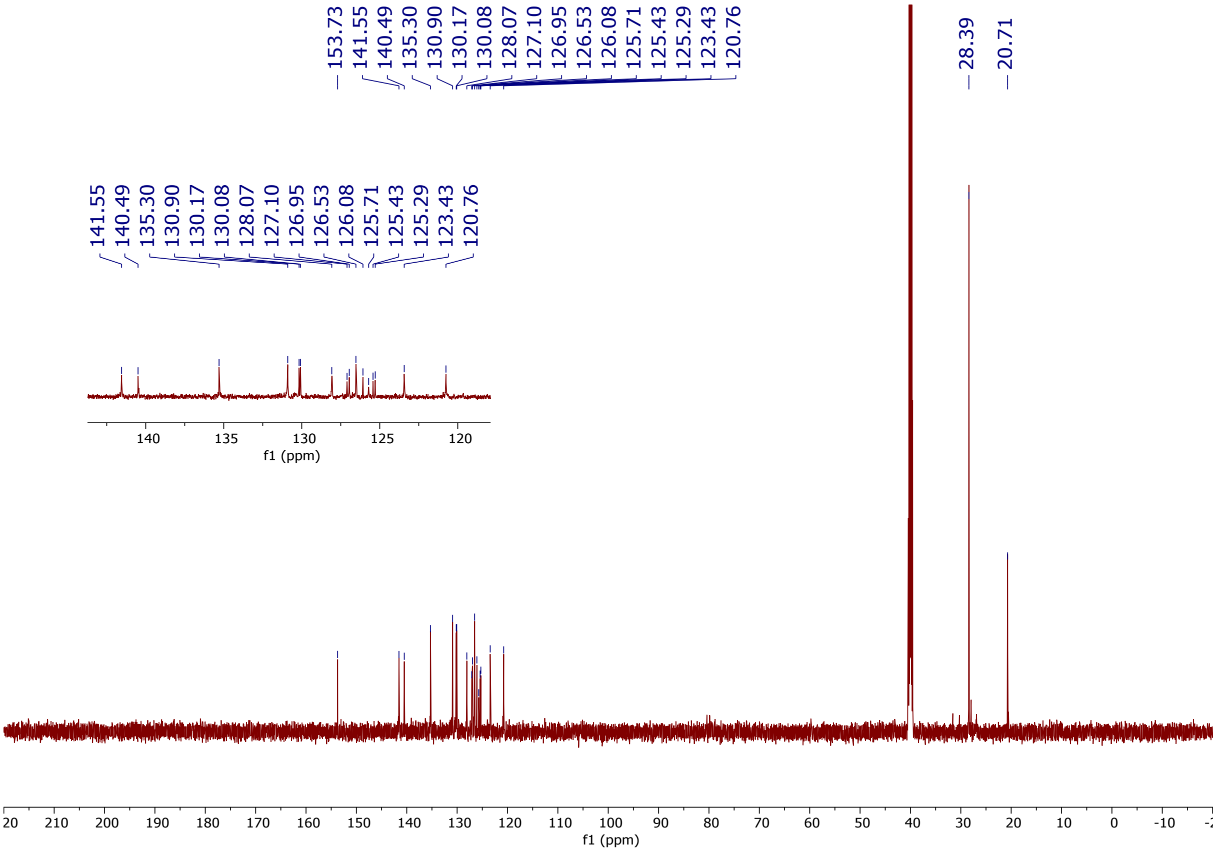

Fig. S29. ^{13}C NMR of **PQ-CHO-PY** in DMSO-d_6.

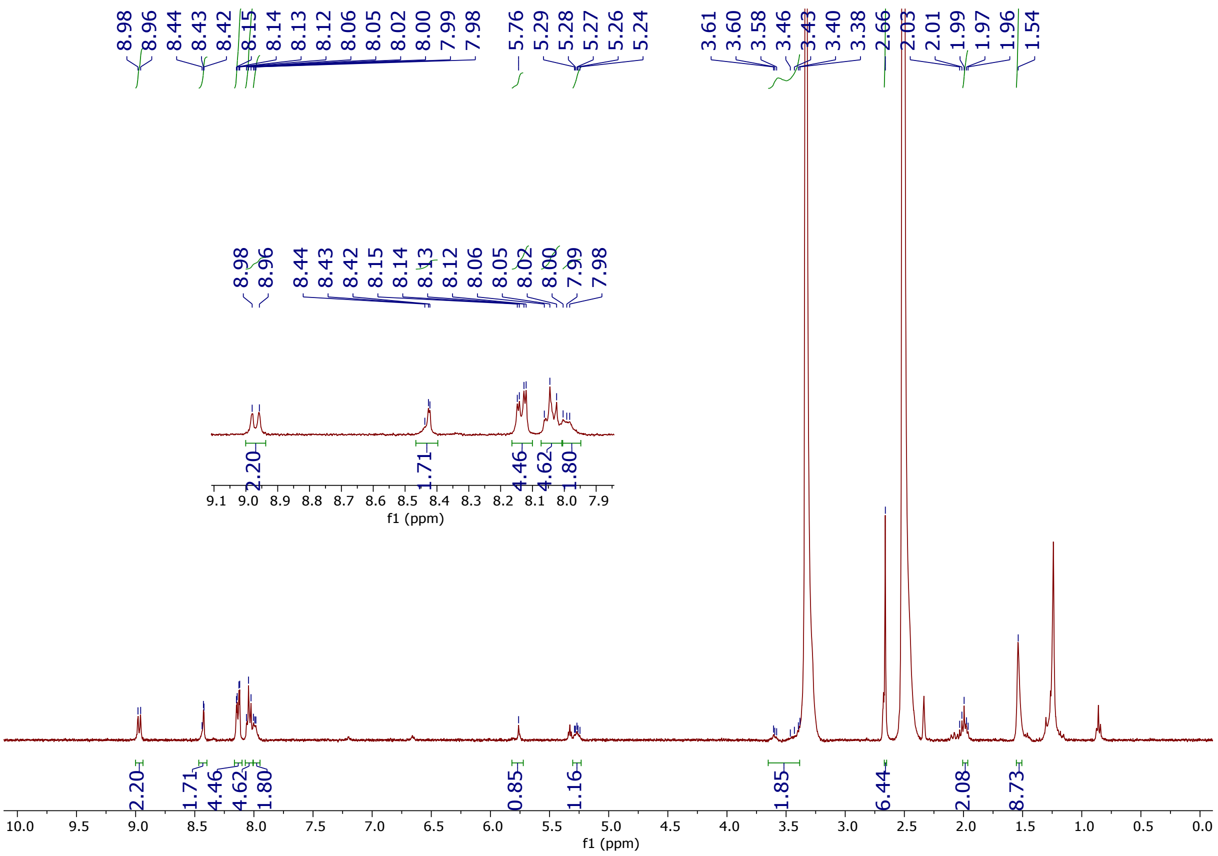

Fig. S30. ^{1}H NMR of **PQ-COCH$_3$-PY** in DMSO-d_6.

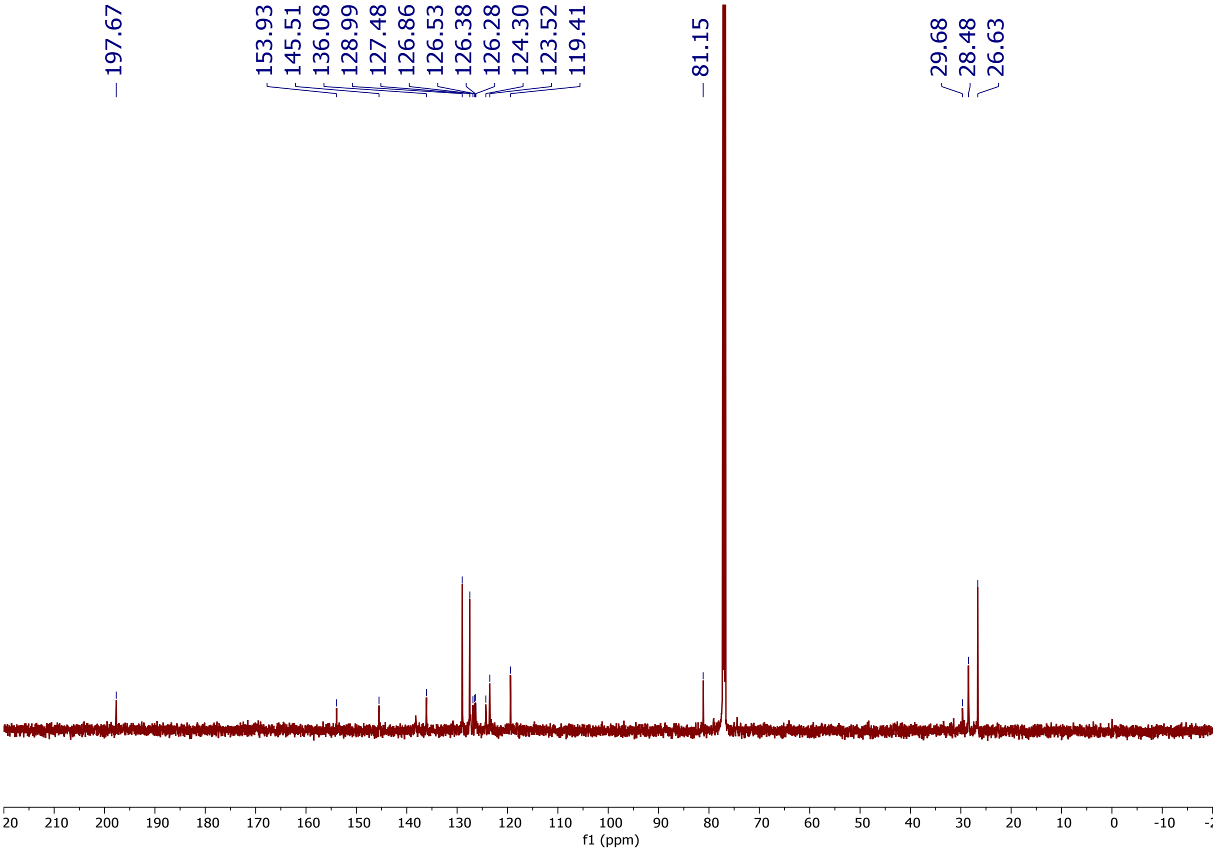

Fig. S31. ^{13}C NMR of **PQ-COCH$_3$-PY** in $CDCl_3$.

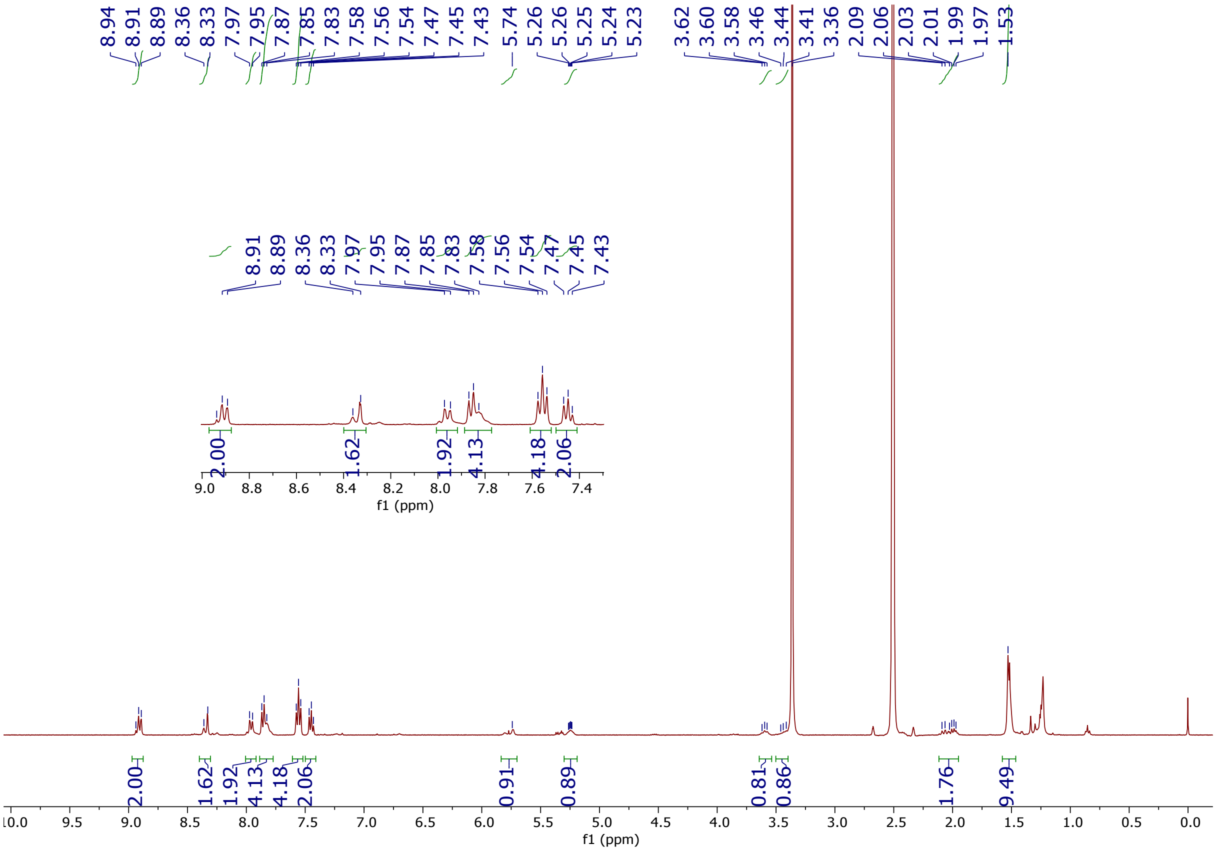

Fig. S32. ^{1}H NMR of **PQ-H-PY** in DMSO-d_6.

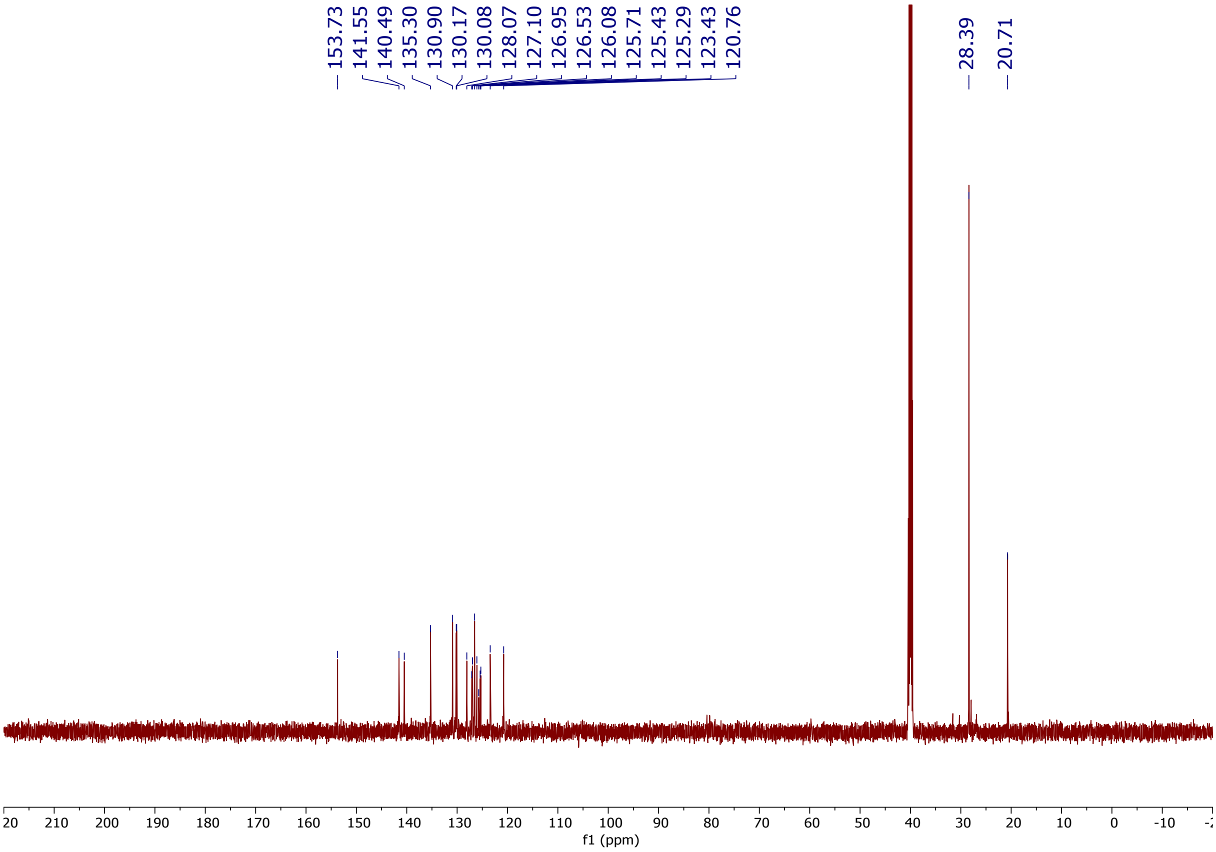

Fig. S33. ^{13}C NMR of **PQ-H-PY** in DMSO-d_6.

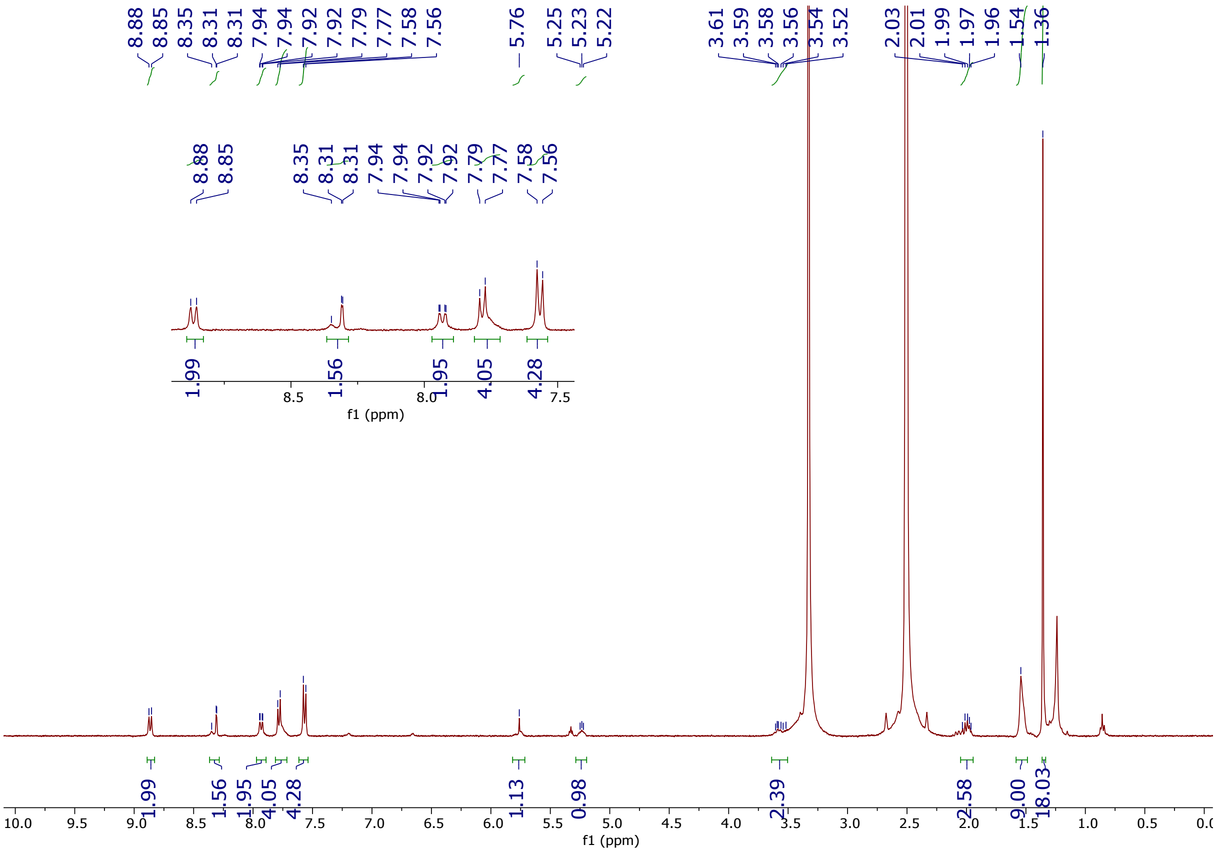

Fig. S34. ^{1}H NMR of **PQ-^{t}Bu-PY** in DMSO-d_6.

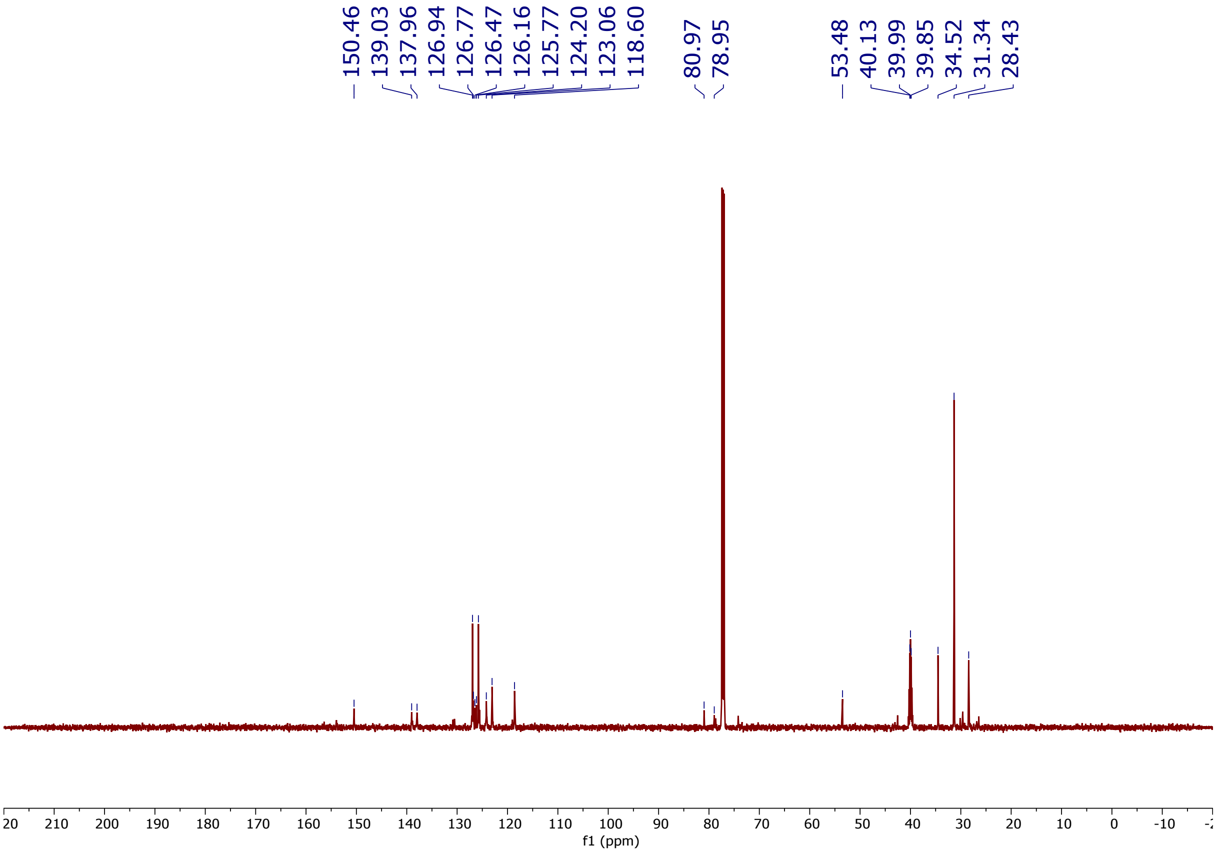

Fig. S35. ^{13}C NMR of **PQ-^{t}Bu-PY** in *CDCl$_3$*.

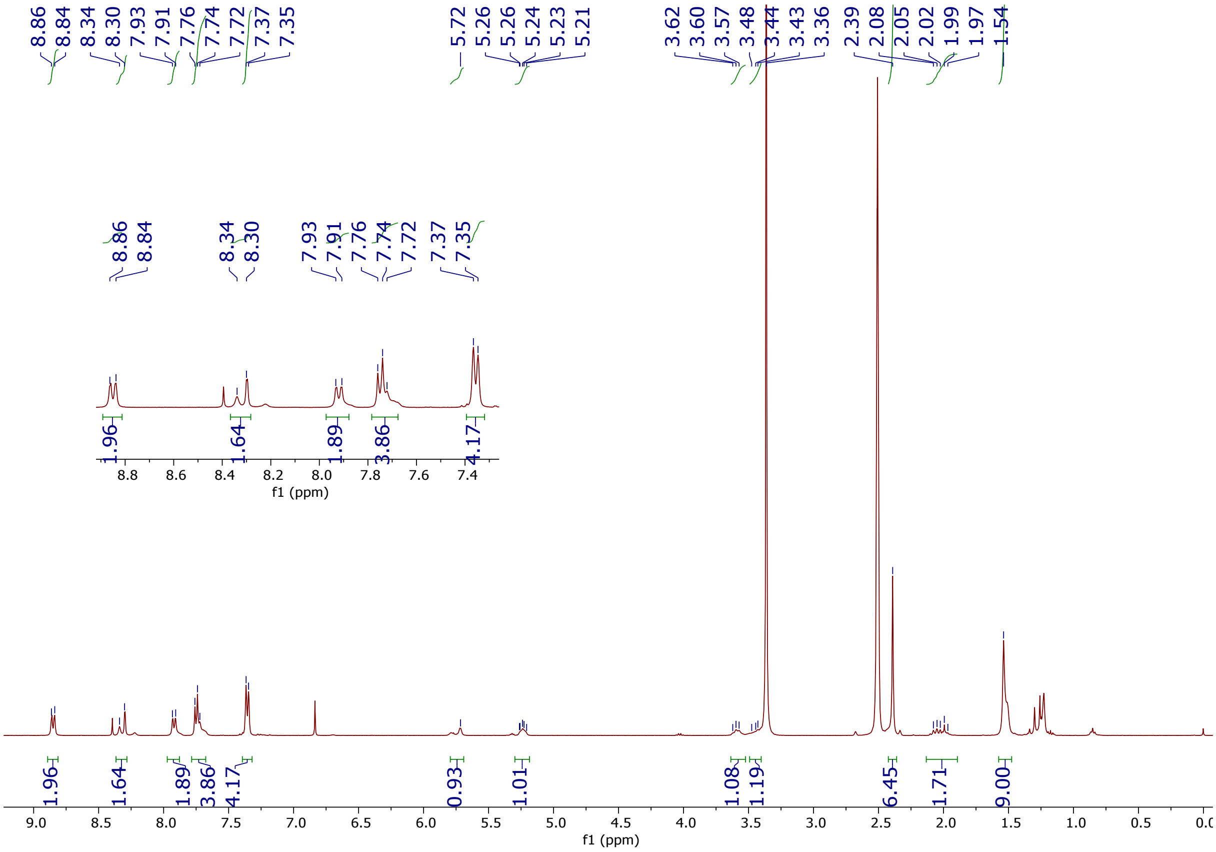

Fig. S36. ^{1}H NMR of **PQ-CH$_3$-PY** in DMSO-d_6.

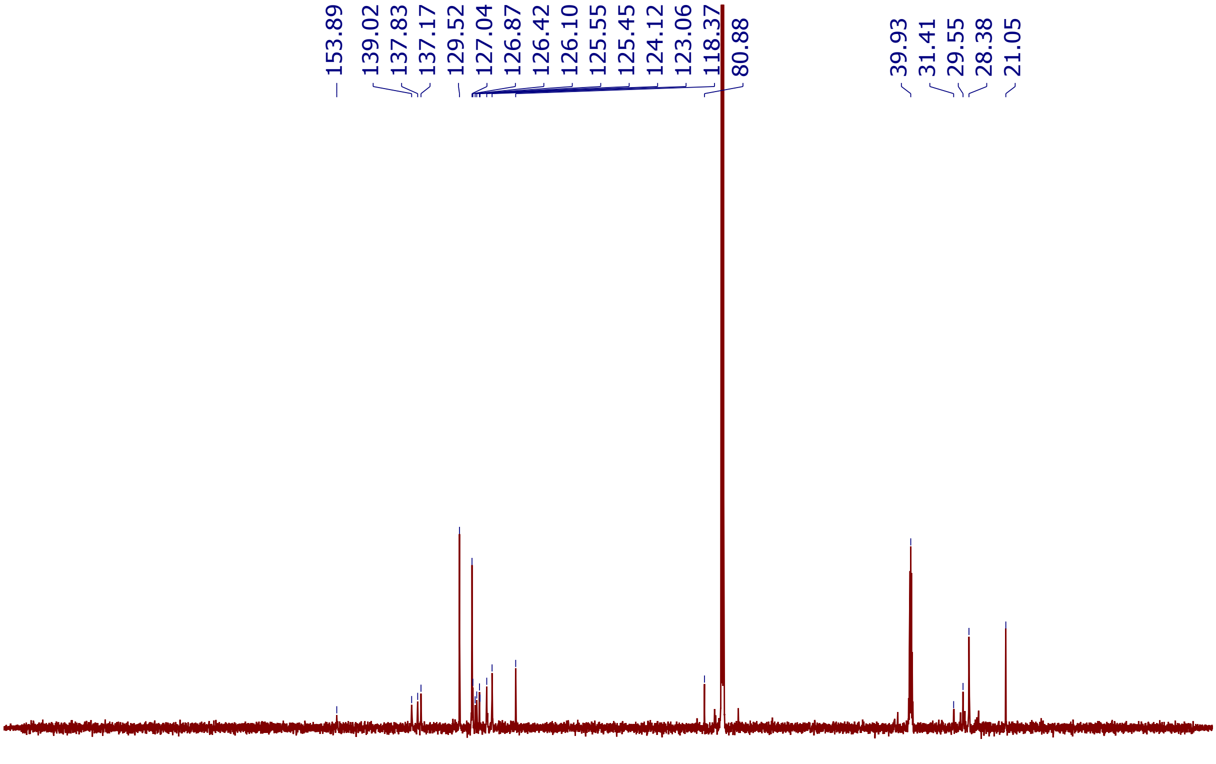

Fig. S37. ^{13}C NMR of **PQ-CH$_3$-PY** in *CDCl$_3$*.

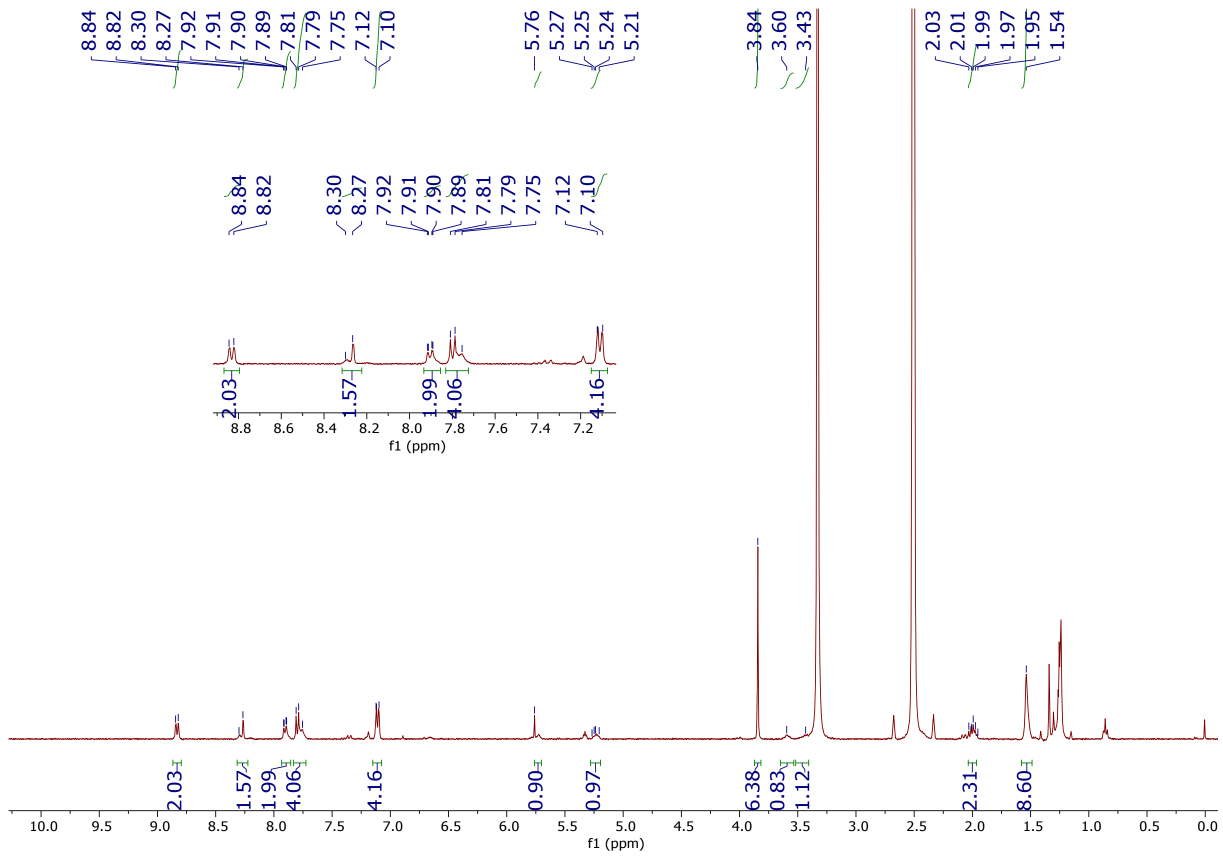

Fig. S38. ^{1}H NMR of **PQ-OCH$_3$-PY** in DMSO-d_6.

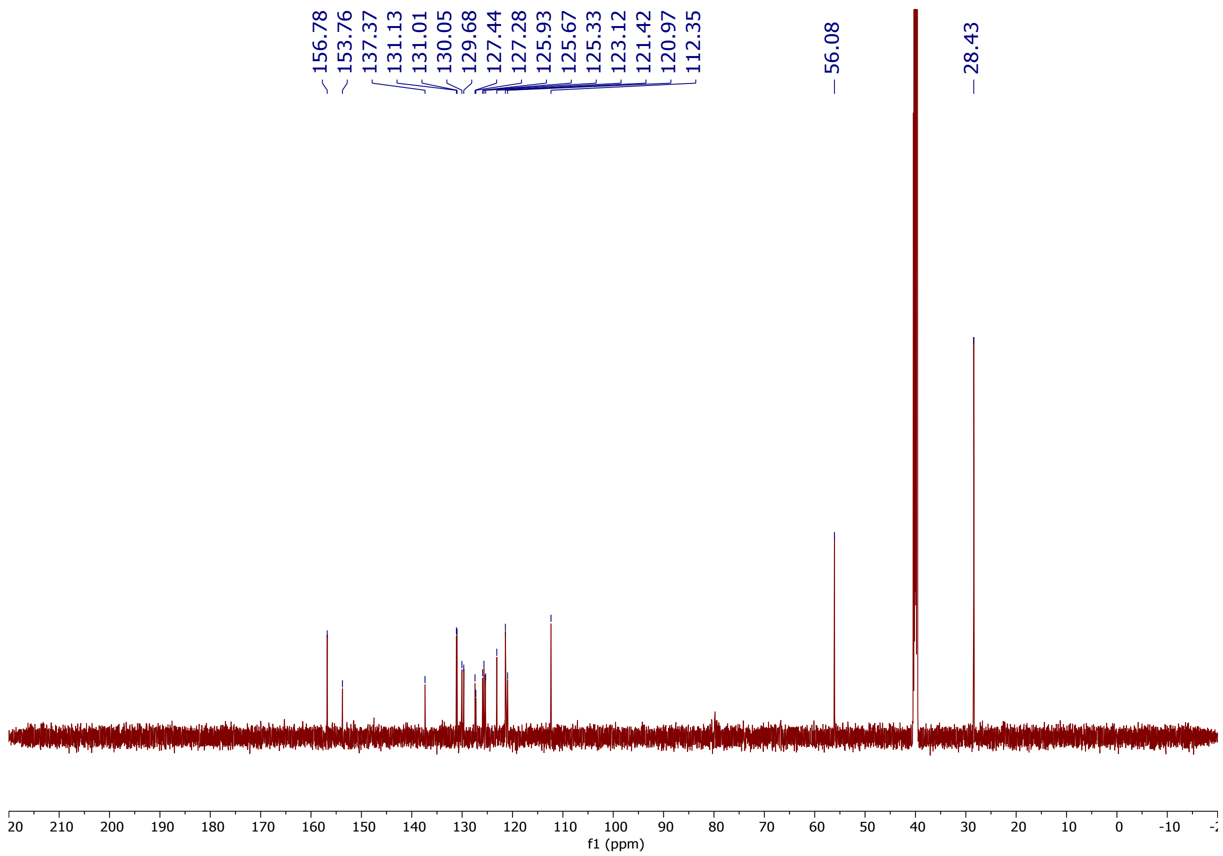

Fig. S39. ^{13}C NMR of **PQ-OCH$_3$-PY** in DMSO-d_6.

4. Photophysical and Photochemical Studies by UV-Vis Spectroscopy and Transient Absorption Spectroscopy

4.1. UV-Vis Spectra

a

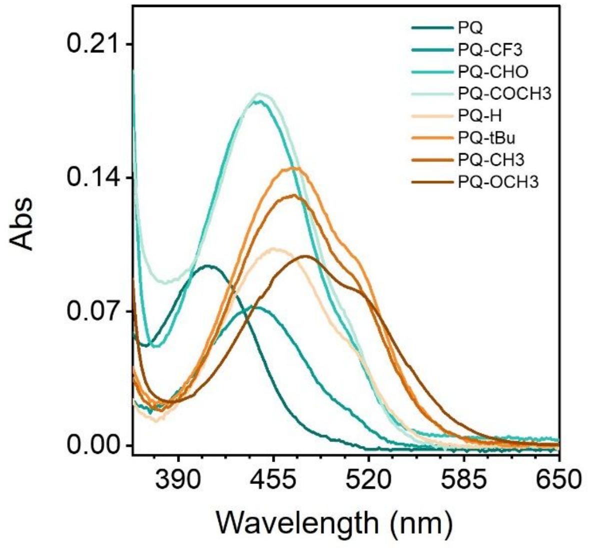

b

PQs	λ_{max} (nm)
PQ	411
PQ-CF_3	440
PQ-CHO	444
PQ-$COCH_3$	448
PQ-H	455
PQ-tBu	467
PQ-CH_3	468
PQ-OCH_3	480, 520

Fig. S40. **a)** Comparison of UV-Vis absorption spectra of **PQs** and **b)** λ_{max}. All measurements were performed in MeCN with 50 µM of **PQ**s at 20 °C.

4.2. Analysis of reactions rates

To assess the different reaction rates of the photo-induced [4+2] cycloaddition of the various phenanthrenequinone (**PQ**s) substrates **PY**, we adapted procedures previously published for the evaluation of the kinetics of chemical[13] and photochemical click reactions.[14] Photochemical transformations are strongly dependent on the used light source and setup, *etc.*, as shown *e.g.* for photocatalyic transformations[15] and photoclick reactions.[16,17] Therefore, we determined the respective rate constants *via* UV-Vis absorption spectroscopy using the fixed LED setup, described above, to achieve reliable comparability of the individual substrates. All reactions were performed at the same concentration (**PQ**s: 50 µM, traps: 500 µM) and under irradiation with the same light source and intensity, independently from the molar attenuation coefficient of the respective **PQ**s at this wavelength. Both the wavelength of irradiation and λ_{obs} were chosen in the spectral regions where only the starting materials, **PQ**, and the photoclick product,

absorb. Rate constants k_{obs} for the different **PQ**s (50 μM) were measured under *pseudo*-first-order conditions with a 10-fold excess of **PY** in MeCN (N_2 atmosphere) by time-dependent analysis. Mixing the appropriate volume of the prepared stock solutions the desired final concentration was derived in sample vials, and the mixture was transferred into a 1 cm optical path quartz optical cuvette, degassed by N_2. Signals were read out by monitoring the absorption signal of the **PQ**s. The data were analyzed using single-exponential fits. All data processing was performed using Origin-pro software.

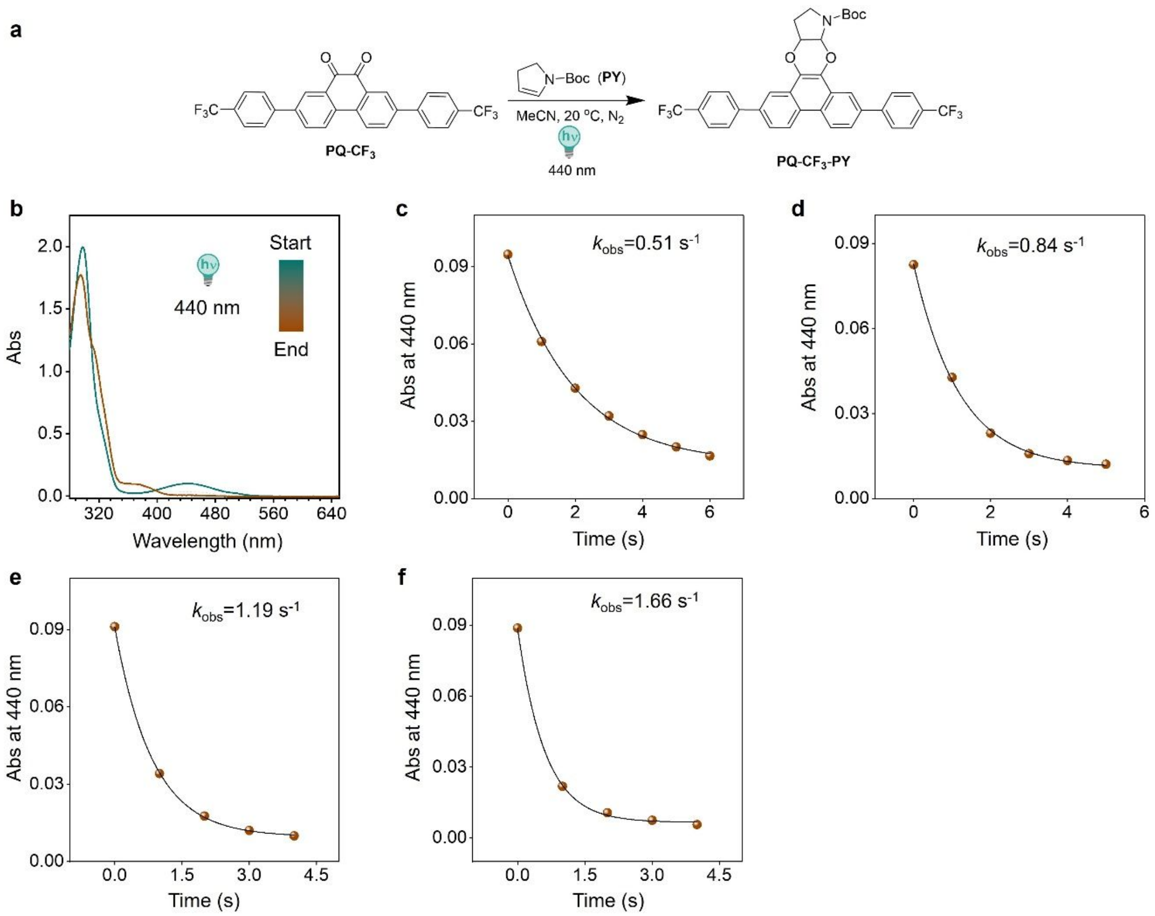

Fig. S41. Photo-induced [4+2] cycloaddition of **PQ-CF$_3$** with **PY**. Reaction scheme **a)**, time-resolved UV-Vis absorption spectra **b)** and kinetic traces of the photocycloaddition between **PQ** (50 μM) with **c) PY** (50 μM), **d) PY** (75 μM) **e) PY** (100 μM) and **f) PY** (150 μM) in 2.5 mL MeCN (N_2 atmosphere) was irradiated with 440 nm LED at 20 °C. The reaction was

monitored by UV-Vis absorption spectra (1 cm cuvette). **PQ-CF$_3$-PY** formation was fitted to an exponential rise to the maximum equation, y = (y$_0$-a) e ^{kobs*t} + b, to give k_{obs}.

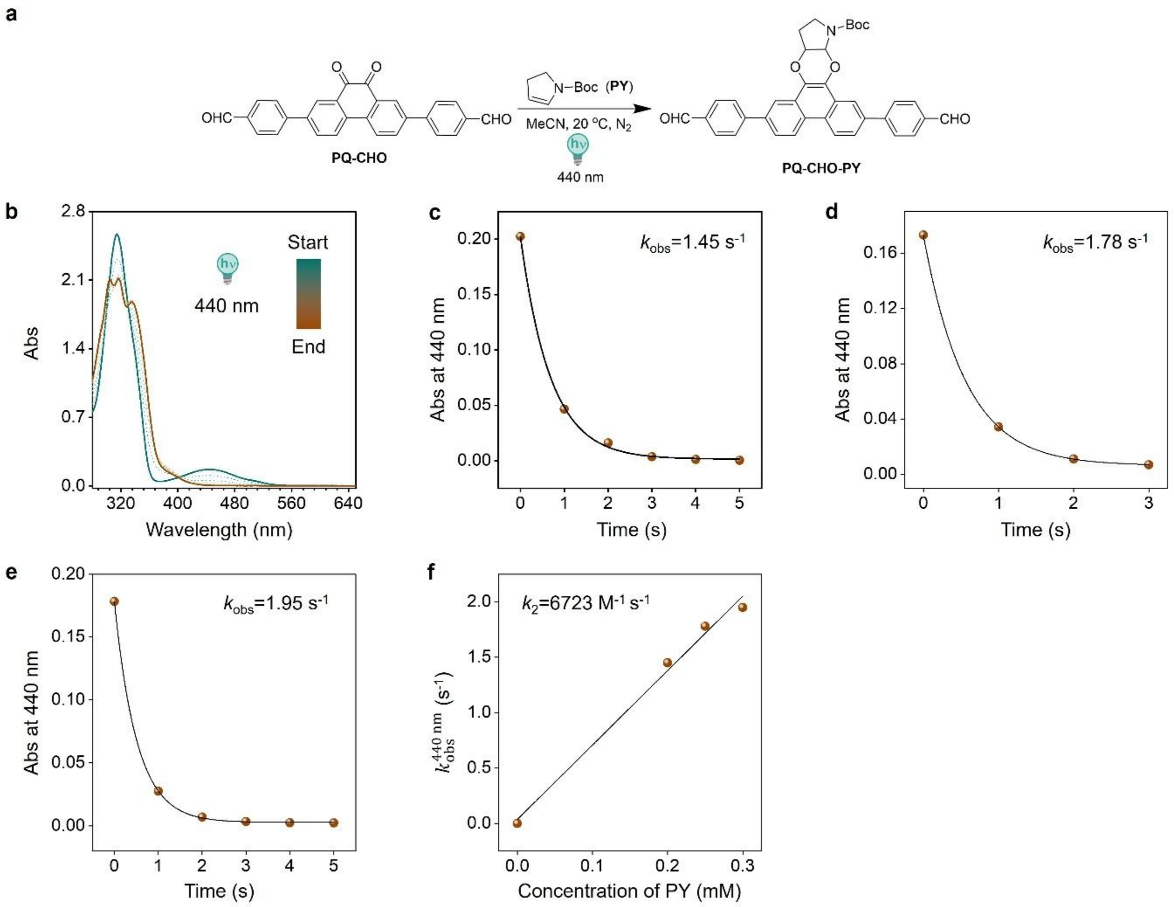

Fig. S42. Photo-induced [4+2] cycloaddition of **PQ-CHO** with **PY**. Reaction scheme **a)**, time-resolved UV-Vis absorption spectra **b)** and kinetic traces of the photocycloaddition between **PQ-CHO** (50 μM) with different concentration of **PY** **c)** 150 μM, **d)** 200 μM, **e)** 250 μM, **f)** 300 μM in 2.5 mL MeCN (N$_2$ atmosphere) was irradiated with 440 nm LED at 20 ^{o}C. The reaction was monitored by UV-Vis absorption spectra (1 cm cuvette). **PQ-CHO-PY** formation was fitted to an exponential rise to the maximum equation, y = (y$_0$-a) e ^{kobs*t} + b, to give k_{obs}. **g)** Plot of k_{obs}^{440} vs **PY** concentration. The second-order rate constant k_2 was determined to be 6723 M^{-1} s^{-1} based on the slope of the fitted line.

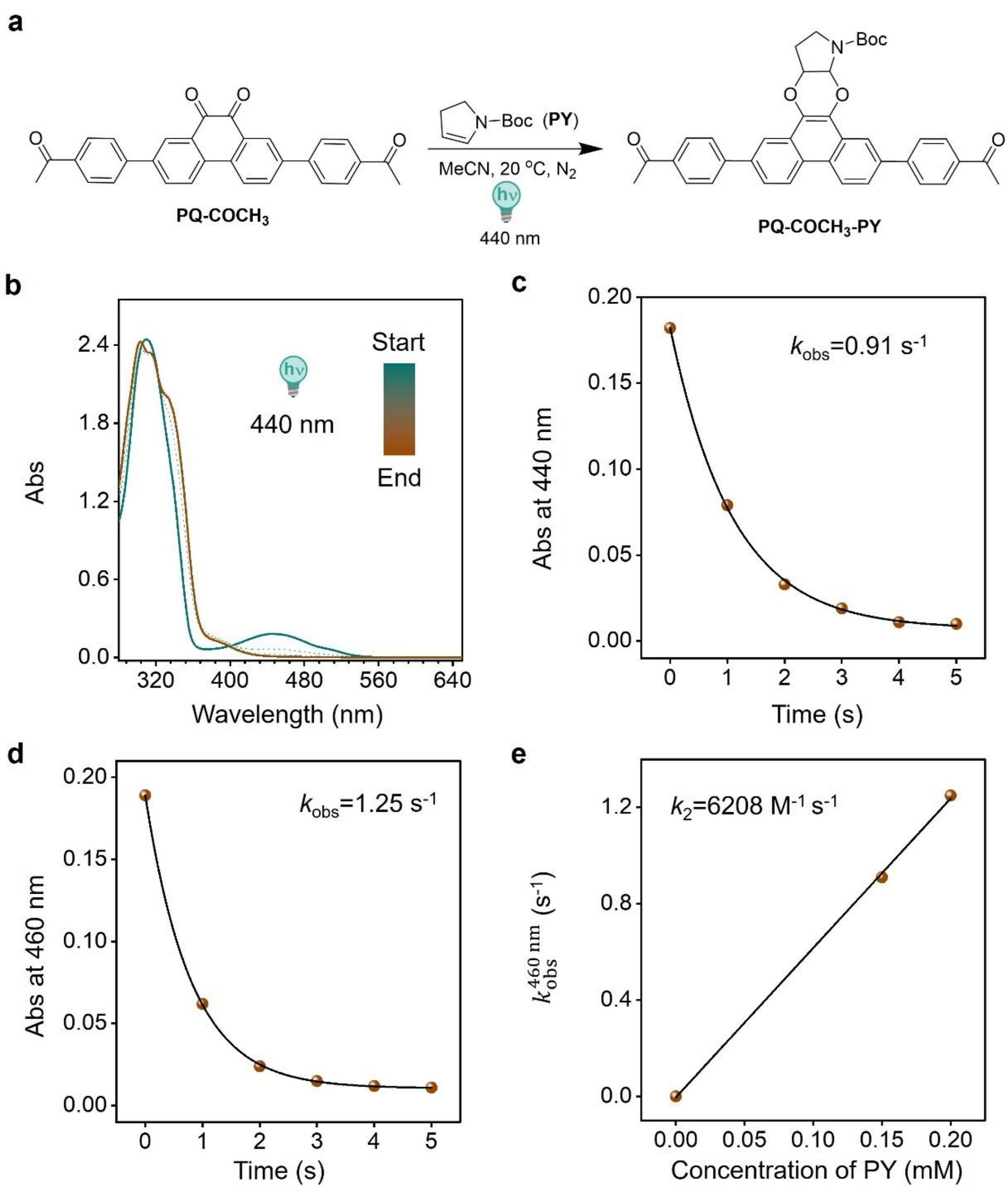

Fig. S43. Photo-induced [4+2] cycloaddition of **PQ-COCH$_3$** with **PY**. Reaction scheme **a)**, time-resolved UV-Vis absorption spectra **b)** and kinetic traces of the photocycloaddition between **PQ-COCH$_3$** (50 μM) with different concentration of **PY** **c)** 150 μM, **d)** 200 μM in 2.5 mL MeCN (N_2 atmosphere) was irradiated with 440 nm LED at 20 ^{o}C. The reaction was monitored by UV-Vis absorption spectra (1 cm cuvette). **PQ-COCH$_3$-PY** formation was fitted to an exponential rise to the maximum equation, $y = (y_0\text{-}a)\ e^{kobs*t} + b$, to give k_{obs}. **e)** Plot of

k_{obs}^{440} *vs* **PY** concentration. The second-order rate constant k_2 was determined to be 6207 M^{-1} s^{-1} based on the slope of the fitted line.

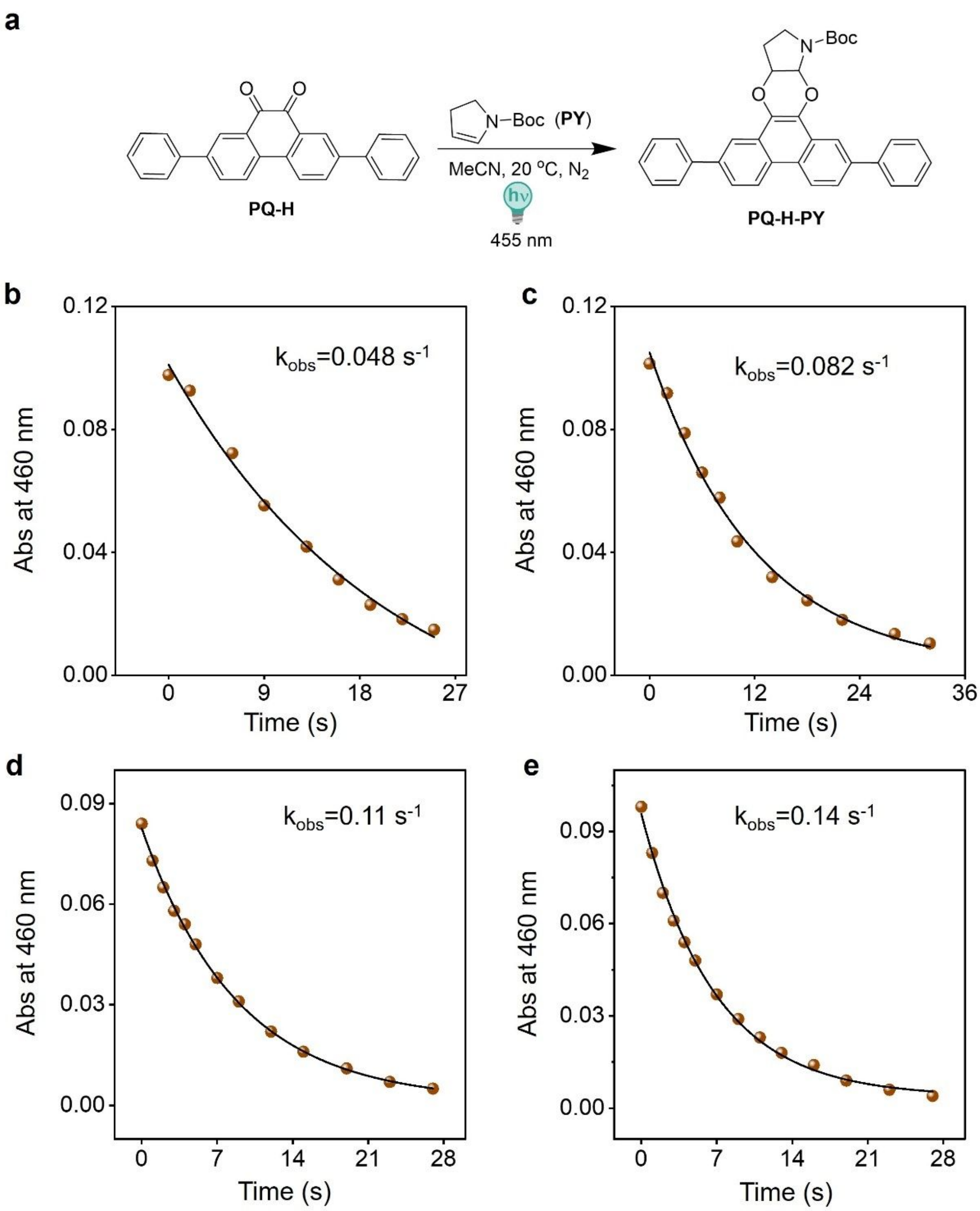

Fig. S44. Reaction scheme **a)** and kinetic traces of the photocycloaddition between **PQ-H** and **PY**. 50 μM **PQ** with different concentrations of **PY** (**(b)** 150 μM , **(c)** 200 μM , **(d)** 250 μM , and **(e)** 300 μM , respectively) in 2.5 mL MeCN (N_2 atmosphere); the reaction mixture was irradiated with 455 nm LED at 20 °C. The formation of the [4+2] cycloaddition product **PQ-**

H-PY was monitored by UV-Vis absorption spectroscopy (λ_{obs}=460 nm, 1 cm cuvette) and the trace was fitted exponentially using the equation, y = (y_0-a) e ^{kobs*t} + b, to give k_{obs}^{460}.

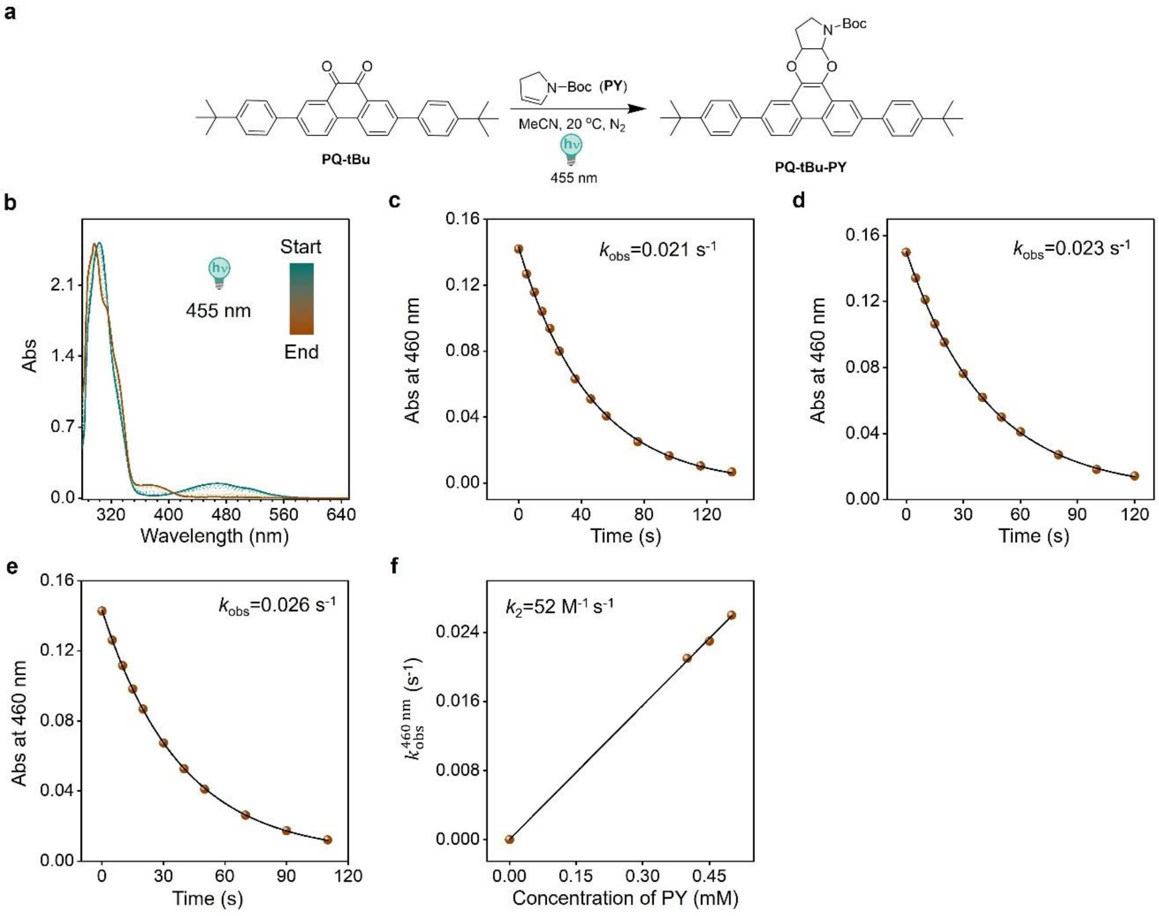

Fig. S45. Photo-induced [4+2] cycloaddition of **PQ-*t*Bu** with **PY**. Reaction scheme **a)**, time-resolved UV-Vis absorption spectra **b)** and kinetic traces of the photocycloaddition between **PQ-*t*Bu** (50 μM) with different concentration of **PY** **c)** 400 μM, **d)** 450 μM, **e)** 500 μM in 2.5 mL MeCN (N_2 atmosphere) was irradiated with 455 nm LED at 20 °C. The reaction was monitored by UV-Vis absorption spectra (1 cm cuvette). **PQ-*t*Bu-PY** formation was fitted to an exponential rise to the maximum equation, y = (y_0-a) e ^{kobs*t} + b, to give k_{obs}. **f)** Plot of k_{obs}^{460} vs **PY** concentration. The second-order rate constant k_2 was determined to be 52 M^{-1} s^{-1} based on the slope of the fitted line.

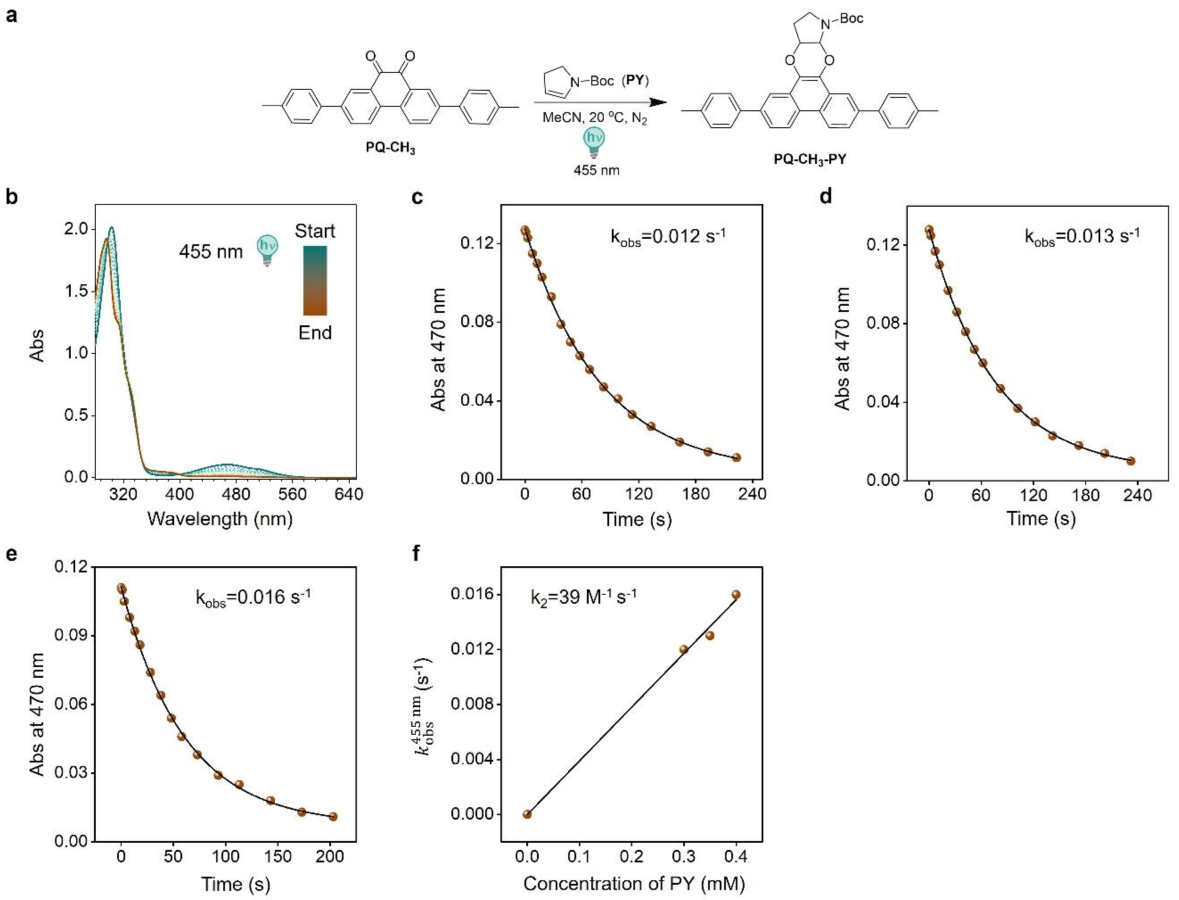

Fig. S46. Photo-induced [4+2] cycloaddition of **PQ-CH$_3$** with **PY**. Reaction scheme **a)**, time-resolved UV-Vis absorption spectra **b)** and kinetic traces of the photocycloaddition between **PQ-CH$_3$** (50 μM) with different concentration of **PY** **c)** 300 μM, **d)** 350 μM, **e)** 400 μM in 2.5 mL MeCN (N_2 atmosphere) was irradiated with 455 nm LED at 20 °C. The reaction was monitored by UV-Vis absorption spectra (1 cm cuvette). **PQ-CH$_3$-PY** formation was fitted to an exponential rise to the maximum equation, y = (y$_0$-a) e $^{kobs\text{*}t}$ + b, to give k_{obs}. **f)** Plot of k_{obs}^{470} vs **PY** concentration. The second-order rate constant k_2 was determined to be 39 M^{-1} s^{-1} based on the slope of the fitted line.

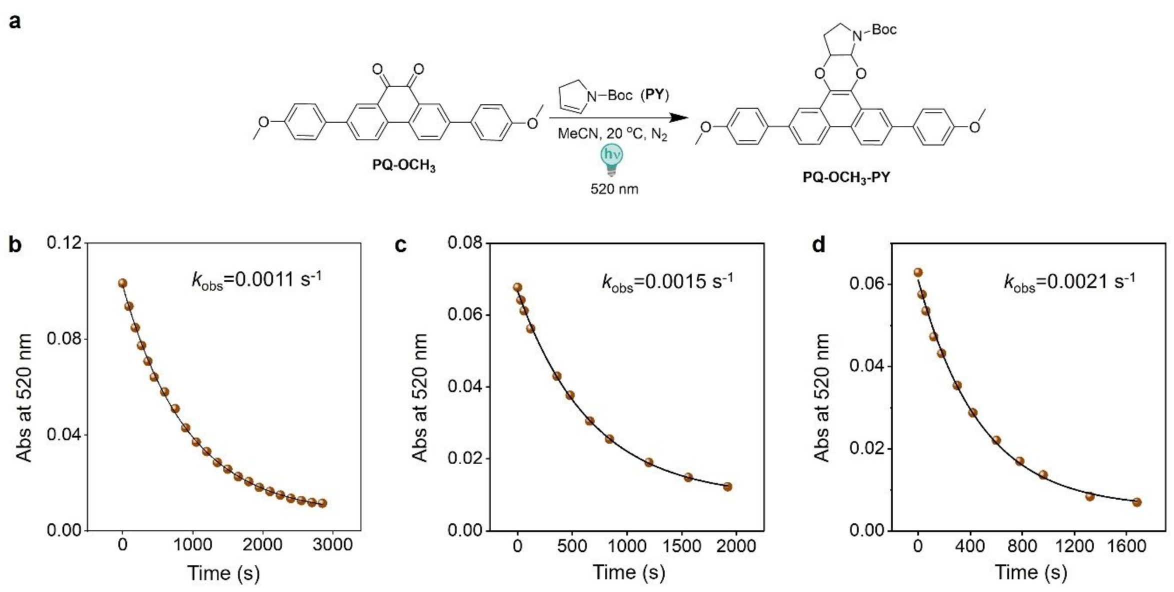

Fig. S47. Reaction scheme **a)** and kinetic traces of the photocycloaddition between **PQ-OCH$_3$** and **PY**. 50 μM **PQ** with different concentrations of **PY** (**(b)** 1.0 mM, (**(c)** 1.5 mM, and **(d)** 2 mM, respectively) in 2.5 mL MeCN (N_2 atmosphere); the reaction mixture was irradiated with 520 nm LED at 20 °C. The formation of the [4+2] cycloaddition product **PQ-OCH$_3$-PY** was monitored by UV-Vis absorption spectroscopy (λ_{obs}=520 nm, 1 cm cuvette) and the trace was fitted exponentially using the equation, y = (y_0-a) e ^{kobs*t} + b, to give k_{obs}^{520}.

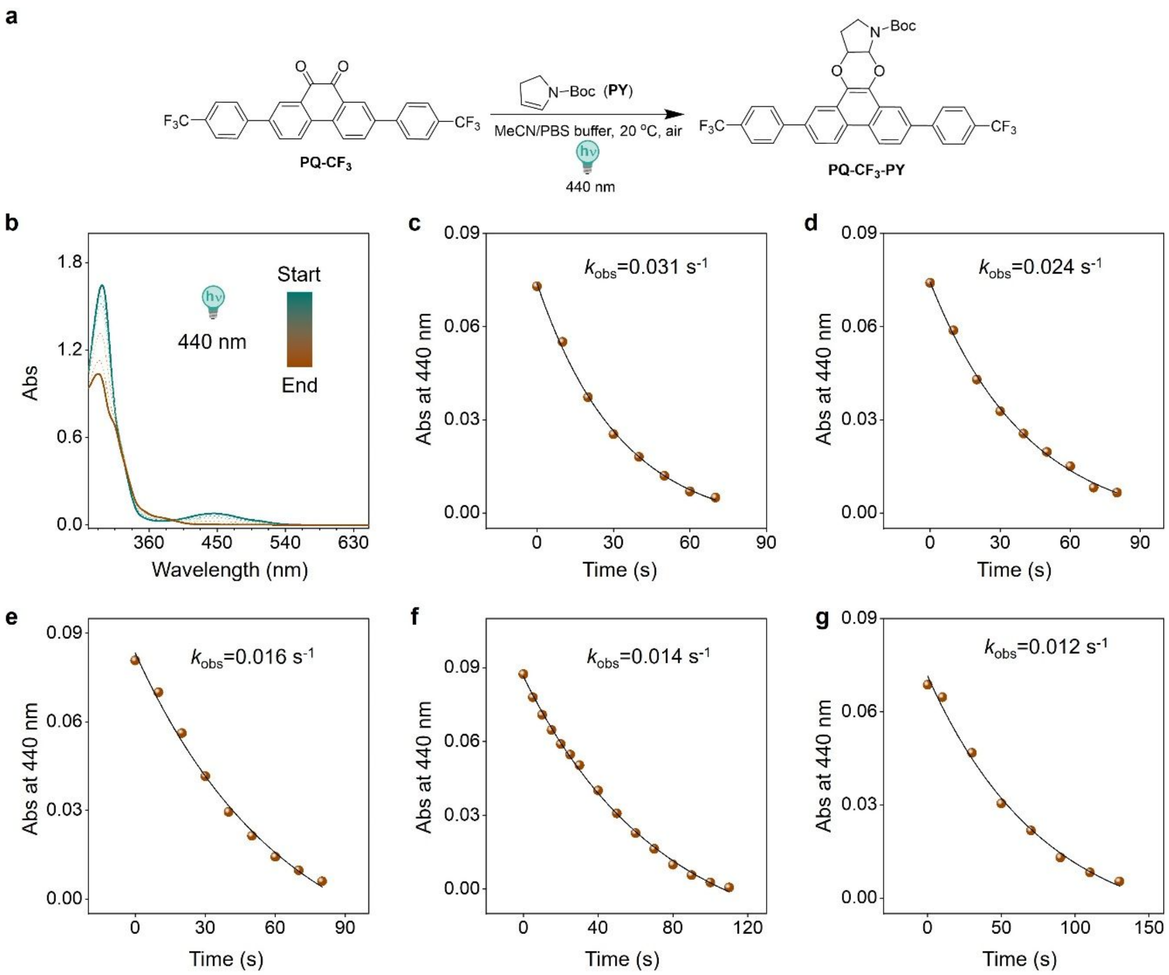

Fig. S48. Reaction scheme **a)**, time-resolved UV-Vis absorption spectra **b)** and kinetic traces of the photocycloaddition between **PQ-CF$_3$** and **PY**. 50 μM **PQ** with 250 μM **PY** in 2.5 mL MeCN/PBS buffer (v/v). From **c)** to **g)** means: 10 % PBS buffer, 20 % PBS buffer, 30 % PBS buffer, 40 % PBS buffer, and 50 % PBS buffer respectively; the reaction mixture was irradiated with 440 nm LED at 20 ^{o}C. The formation of the [4+2] cycloaddition product **PQ-CF$_3$-PY** was monitored by UV-Vis absorption spectroscopy (λ_{obs}=440 nm, 1 cm cuvette) and the trace was fitted exponentially using the equation, y = (y_0-a) e ^{kobs*t} + b, to give k_{obs}^{440}.

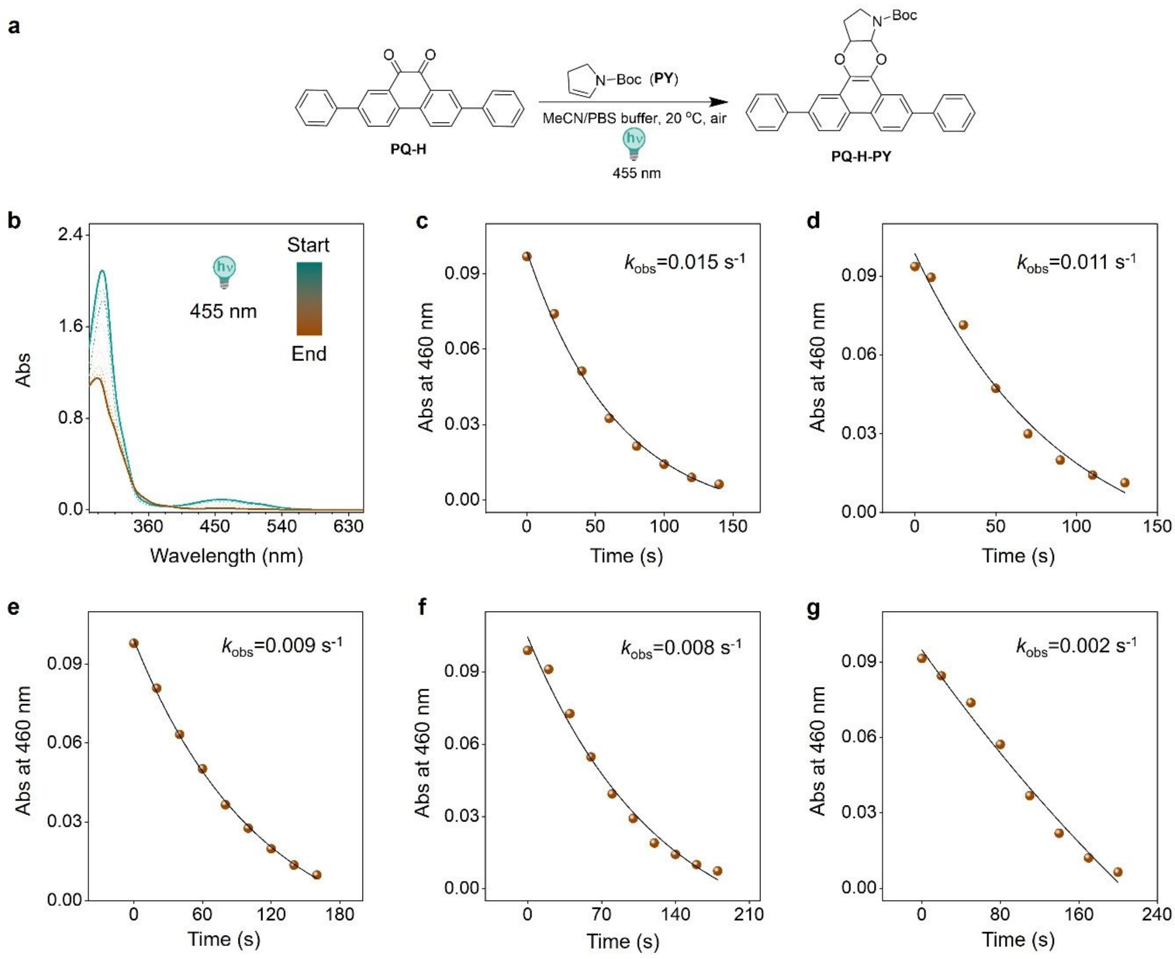

Fig. S49. Reaction scheme **a)**, time-resolved UV-Vis absorption spectra **b)** and kinetic traces of the photocycloaddition between **PQ-H** and **PY**. 50 μM **PQ** with 250 μM **PY** in 2.5 mL MeCN/PBS buffer (v/v). From **c)** to **g)** means: 10 % PBS buffer, 20 % PBS buffer, 30 % PBS buffer, 40 % PBS buffer, and 50 % PBS buffer respectively; the reaction mixture was irradiated with 455 nm LED at 20 °C. The formation of the [4+2] cycloaddition product **PQ-H-PY** was monitored by UV-Vis absorption spectroscopy (λ_{obs}=460 nm, 1 cm cuvette) and the trace was fitted exponentially using the equation, y = (y_0-a) e ^{kobs*t} + b, to give k_{obs}^{455}.

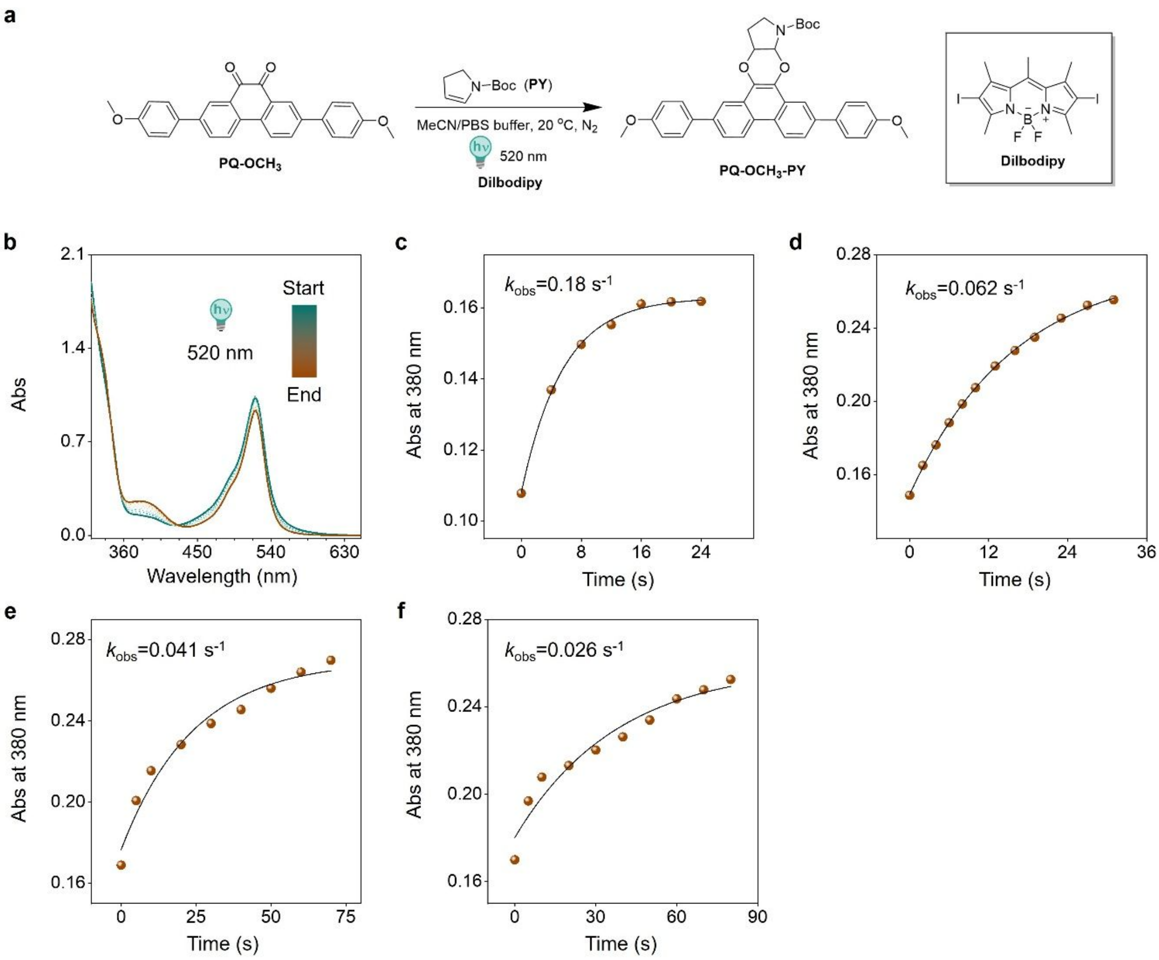

Fig. S50. Reaction scheme **a)**, time-resolved UV-Vis absorption spectra **b)** and kinetic traces of the photocycloaddition between **PQ-OCH$_3$** and **PY** in the present of **DiIBodipy**. 50 μM **PQ**, 250 μM **PY** and 25 μM **DiIBodipy** in 2.5 mL MeCN/PBS buffer (v/v). From **c)** to **f)** means: 10 % PBS buffer, 30 % PBS buffer, 40 % PBS buffer, and 50 % PBS buffer respectively; the reaction mixture was irradiated with 520 nm LED at 20 ^{o}C. The formation of the [4+2] cycloaddition product **PQ-OCH$_3$-PY** was monitored by UV-Vis absorption spectroscopy (λ_{obs}=520 nm, 1 cm cuvette) and the trace was fitted exponentially using the equation, y = (y_0-a) e ^{kobs*t} + b, to give k_{obs}^{520}.

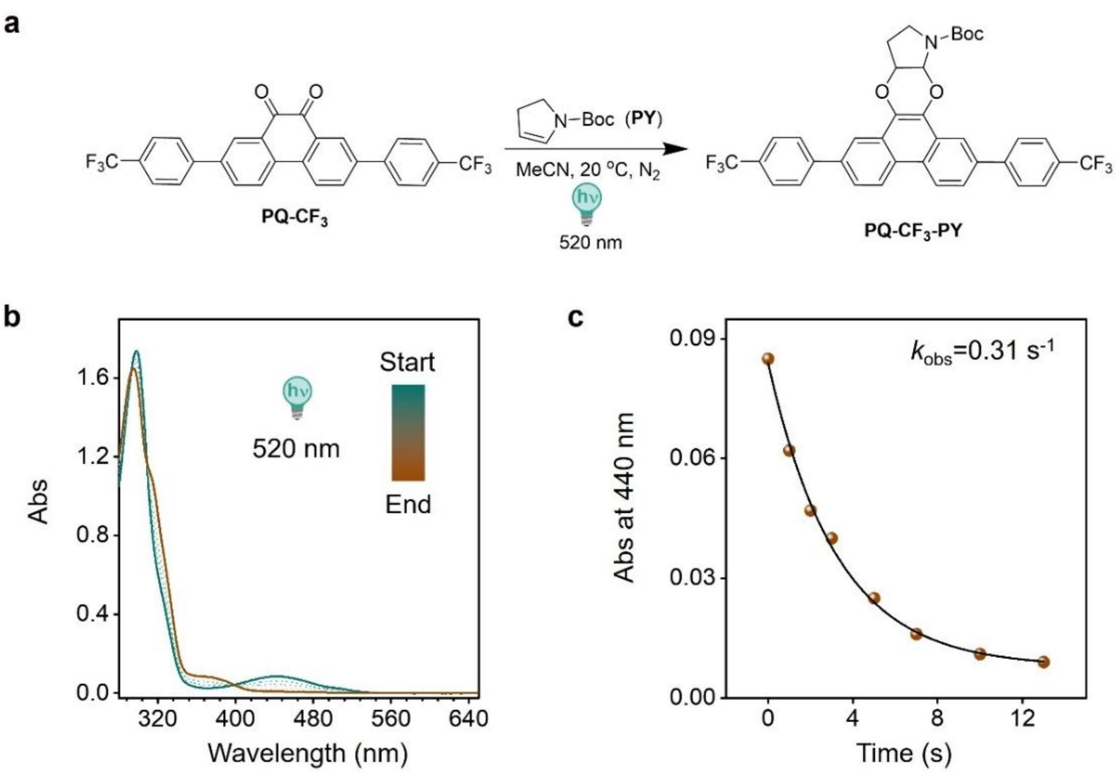

Fig. S51. Photo-induced [4+2] cycloaddition of **PQ-CF$_3$** with **PY**. Reaction scheme **a)**, time-resolved UV-Vis absorption spectra **b)** and kinetic traces of the photocycloaddition between **PQ-CF$_3$** (50 μM) and **PY** (500 μM) in 2.5 mL MeCN (N_2 atmosphere) was irradiated with 520 nm LED at 20 ^{o}C. The reaction was monitored by UV-Vis absorption spectra (λ_{obs}=440 nm, 1 cm cuvette). **PQ-CF$_3$-PY** formation was fitted to an exponential rise to the maximum equation, y = (y_0-a) e ^{kobs*t} + b, to give k_{obs}.

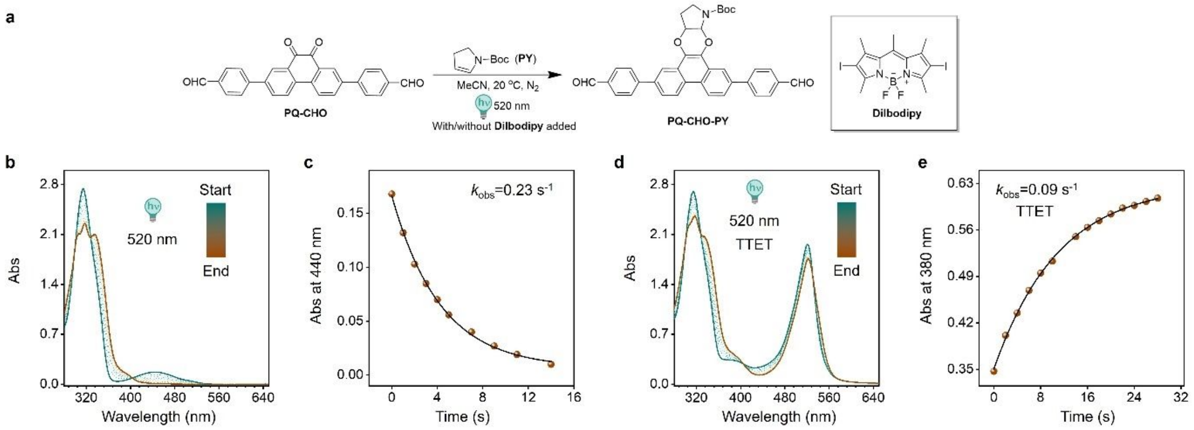

Fig. S52. Photo-induced [4+2] cycloaddition of **PQ-CHO** with **PY** in the present/absence of **DiIbodipy**. Reaction scheme **a)**, time-resolved UV-Vis absorption spectra and kinetic traces of the photocycloaddition between **PQ-CHO** (50 μM) and **PY** (500 μM) in 2.5 mL MeCN (N_2 atmosphere) without (**b, c**) and with (**d, e**) **DiIbodipy** (50 μM) addition respectively, these samples were irradiated with 520 nm LED at 20 ^oC. The reaction was monitored by UV-Vis absorption spectra (1 cm cuvette). **PQ-CHO-PY** formation was fitted to an exponential rise to the maximum equation, $y = (y_0\text{-}a)\ e^{k_{obs}*t} + b$, to give k_{obs}.

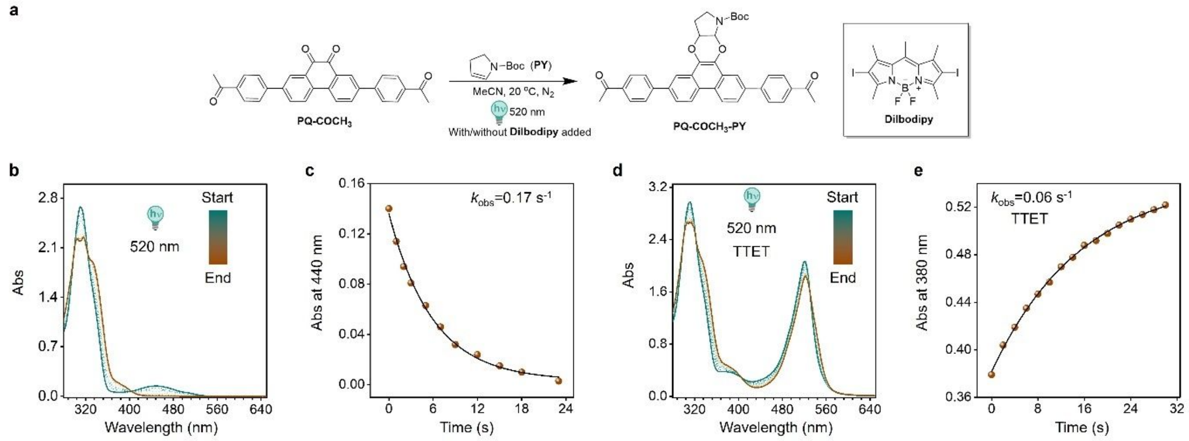

Fig. S53. Photo-induced [4+2] cycloaddition of **PQ-COCH$_3$** with **PY** in the present/absence of **DiIbodipy**. Reaction scheme **a)**, time-resolved UV-Vis absorption spectra and kinetic traces of the photocycloaddition between **PQ-COCH$_3$** (50 μM) and **PY** (500 μM) in 2.5 mL MeCN (N_2 atmosphere) without (**b, c**) and with (**d, e**) **DiIbodipy** (50 μM) addition respectively, these samples were irradiated with 520 nm LED at 20 ^oC. The reaction was monitored by UV-Vis absorption spectra (1 cm cuvette). **PQ-COCH$_3$-PY** formation was fitted to an exponential rise to the maximum equation, $y = (y_0\text{-}a)\ e^{k_{obs}*t} + b$, to give k_{obs}.

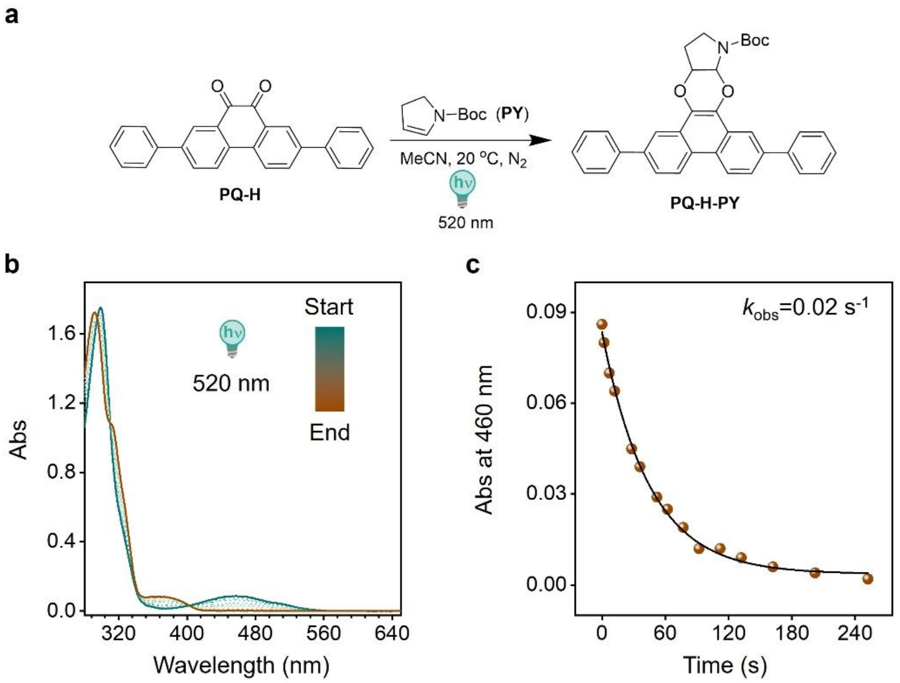

Fig. S54. Photo-induced [4+2] cycloaddition of **PQ-H** with **PY**. Reaction scheme **a)**, time-resolved UV-Vis absorption spectra **b)** and kinetic traces of the photocycloaddition between **PQ-H** (50 μM) and **PY** (500 μM) in 2.5 mL MeCN (N_2 atmosphere) was irradiated with 520 nm LED at 20 °C. The reaction was monitored by UV-Vis absorption spectra (1 cm cuvette). **PQ-H-PY** formation was fitted to an exponential rise to the maximum equation, y = (y_0-a) e ^{kobs*t} + b, to give k_{obs}.

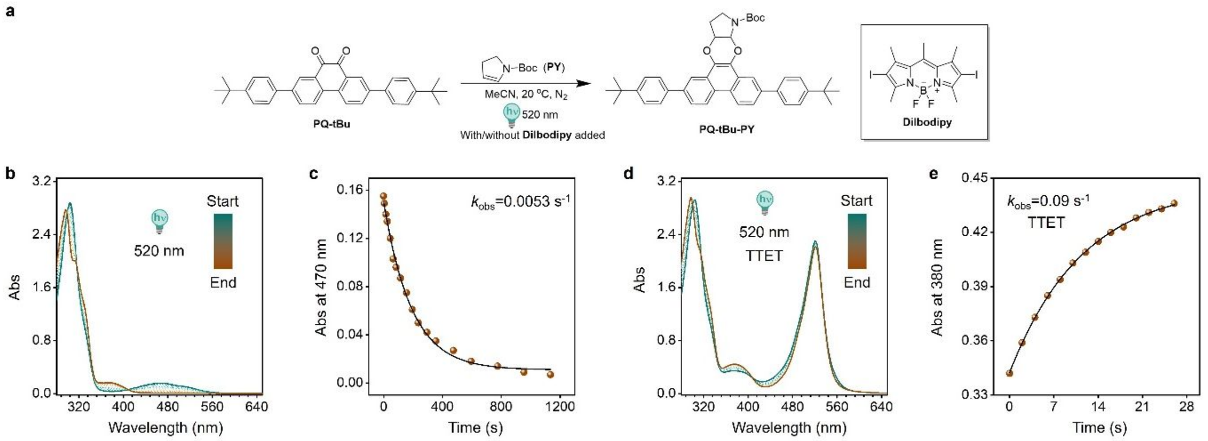

Fig. S55. Photo-induced [4+2] cycloaddition of **PQ-*t*Bu** with **PY** in the present/absence of **DiIbodipy**. Reaction scheme **a)**, time-resolved UV-Vis absorption spectra and kinetic traces of

the photocycloaddition between **PQ-*t*Bu** (50 μM) and **PY** (500 μM) in 2.5 mL MeCN (N_2 atmosphere) without (**b, c**) and with (**d, e**) **DiIbodipy** (50 μM) addition respectively, these samples were irradiated with 520 nm LED at 20 °C. The reaction was monitored by UV-Vis absorption spectra (1 cm cuvette). **PQ-*t*Bu-PY** formation was fitted to an exponential rise to the maximum equation, $y = (y_0\text{-}a)\ e^{kobs*t} + b$, to give k_{obs}.

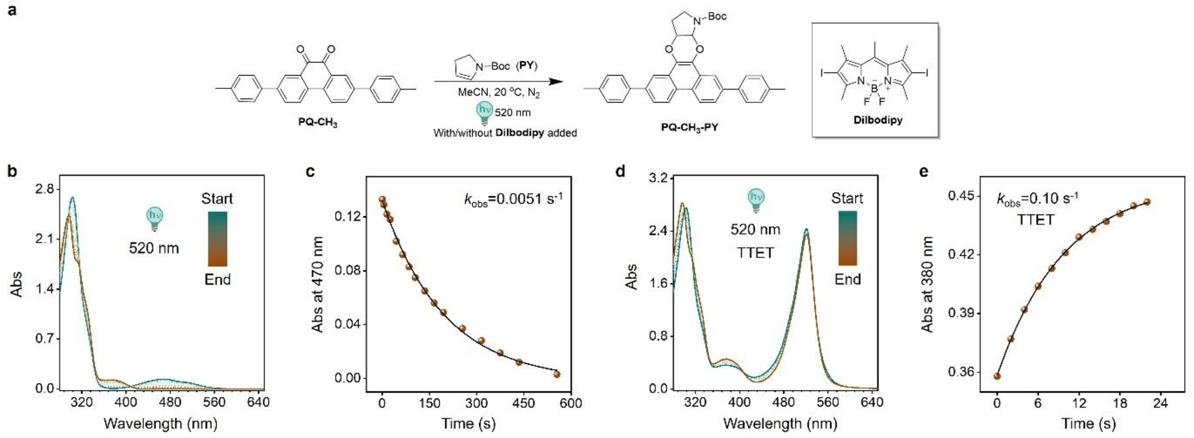

Fig. S56. Photo-induced [4+2] cycloaddition of **PQ-CH$_3$** with **PY** in the present/absence of **DiIbodipy**. Reaction scheme **a)**, time-resolved UV-Vis absorption spectra and kinetic traces of the photocycloaddition between **PQ-CH$_3$** (50 μM) and **PY** (500 μM) in 2.5 mL MeCN (N_2 atmosphere) without (**b, c**) and with (**d, e**) **DiIbodipy** (50 μM) addition respectively, these samples were irradiated with 520 nm LED at 20 °C. The reaction was monitored by UV-Vis absorption spectra (1 cm cuvette). **PQ-CH$_3$-PY** formation was fitted to an exponential rise to the maximum equation, $y = (y_0\text{-}a)\ e^{kobs*t} + b$, to give k_{obs}.

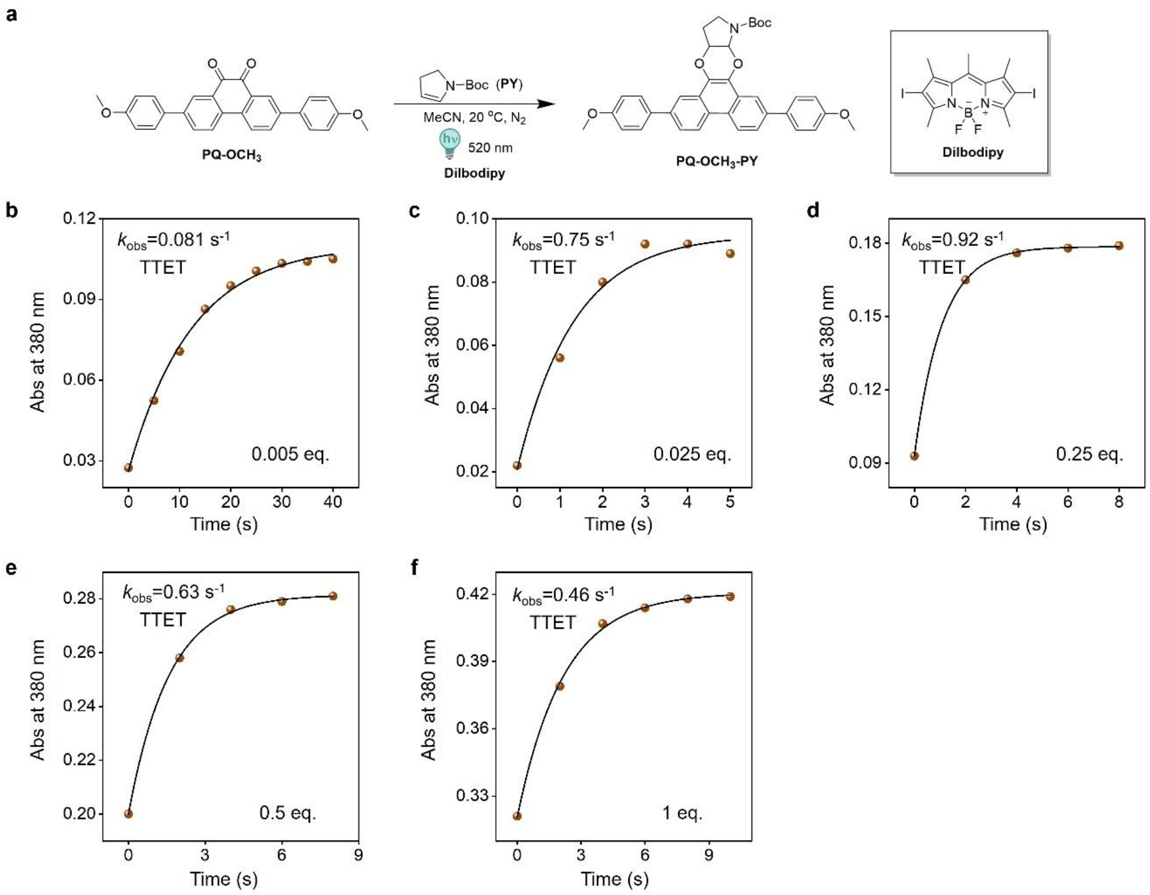

Fig. S57. Photocycloaddition between **PQ-OCH$_3$** and **PY** in the present of different amounts of **DiIbodipy**. **a)** reaction scheme, and kinetic trace of the photocycloaddition between **PQ-PQ-OCH$_3$** (50 μM) and **PY** (500 μM) in the presence of **b)** 0.005 eq, **c)** 0.025 eq, **d)** 0.25 eq, **e)** 0.5 eq, and **f)** 1 eq of **DiIbodipy** in MeCN (2.5 mL, N_2 atmosphere) respectively; the reaction mixture was irradiated with a 520 nm LED at 20 °C. The reaction was monitored by UV-Vis absorption spectroscopy (1 cm cuvette). **PQ-OCH$_3$-PY** formation following equation: y = (y_0-a) e ^{kobs*t} + b, provided k_{obs}^{520}.

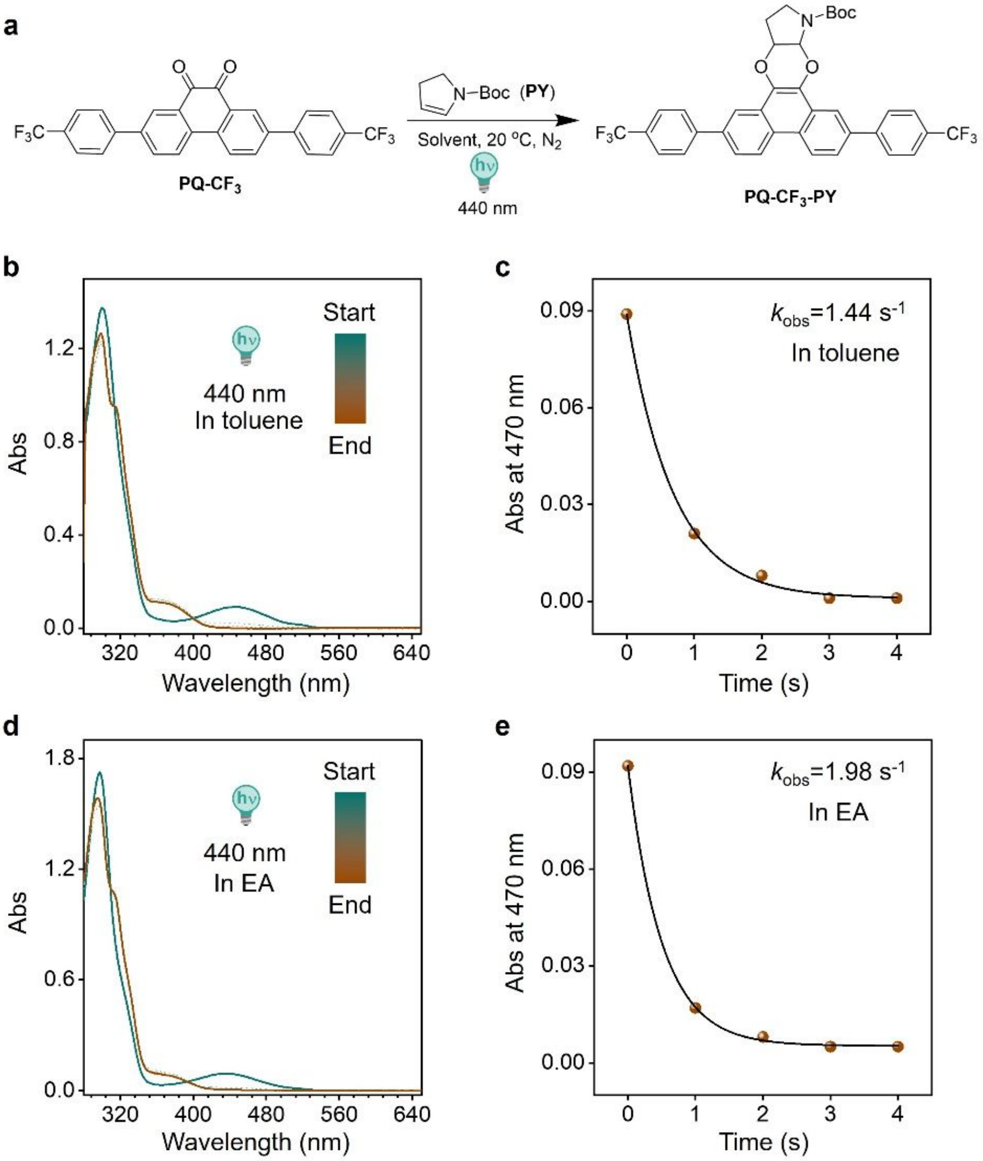

Fig. S58. Solvent effect on the **PQ-CF$_3$/PY** photoclick reaction. Reaction scheme **a)**, time-resolved UV-Vis absorption spectra and kinetic traces of the photocycloaddition between **PQ-CF$_3$** (50 μM) and **PY** (500 μM) in 2.5 mL toluene (**b, c**) and EA (**d, e**) (N_2 atmosphere) respectively, these samples were irradiated with 440 nm LED at 20 ^{o}C. The reaction was monitored by UV-Vis absorption spectra (1 cm cuvette). **PQ-CF$_3$-PY** formation was fitted to an exponential rise to the maximum equation, $y = (y_0\text{-}a)\ e^{k_{obs}*t} + b$, to give k_{obs}.

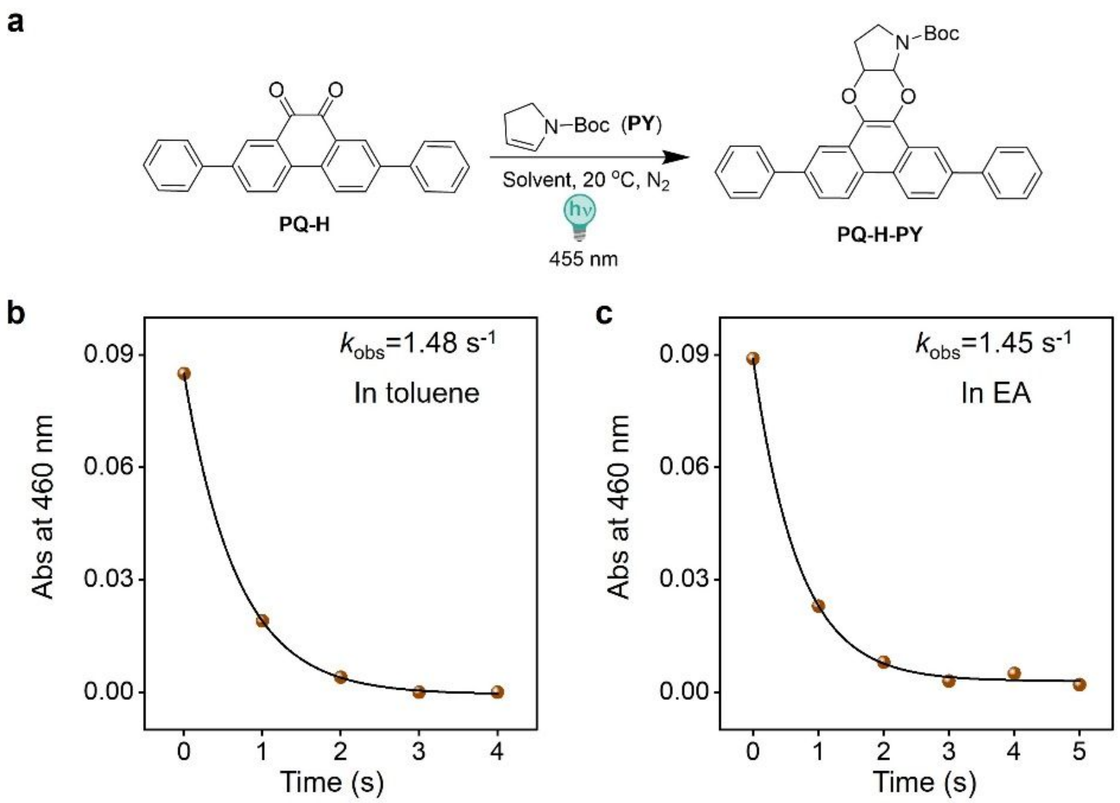

Fig. S59. Solvent effect on the **PQ-H/PY** photoclick reaction. Reaction scheme **a)**, and kinetic traces of the photocycloaddition between **PQ-H** (50 μM) and **PY** (500 μM) in 2.5 mL toluene (**b**) and EA (**c**) (N_2 atmosphere) respectively, these samples were irradiated with 455 nm LED at 20 °C. The reaction was monitored by UV-Vis absorption spectra (1 cm cuvette). **PQ-H-PY** formation was fitted to an exponential rise to the maximum equation, $y = (y_0\text{-}a)\ e^{k_{obs}*t} + b$, to give k_{obs}.

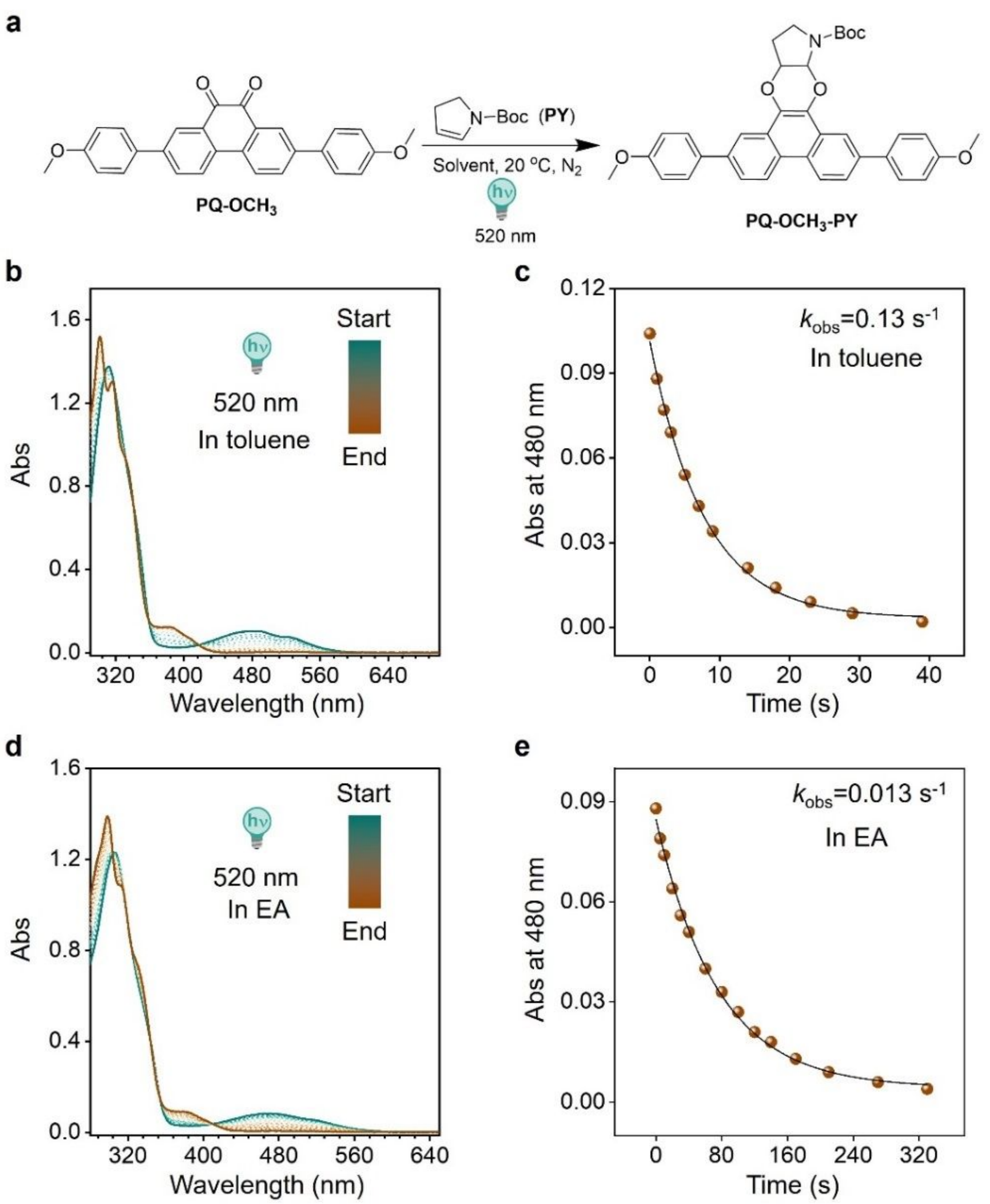

Fig. S60. Solvent effect on the **PQ-OCH$_3$/PY** photoclick reaction. Reaction scheme **a)**, time-resolved UV-Vis absorption spectra and kinetic traces of the photocycloaddition between **PQ-OCH$_3$** (50 μM) and **PY** (500 μM) in 2.5 mL toluene (**b, c**) and EA (**d, e**) (N_2 atmosphere) respectively, these samples were irradiated with 440 nm LED at 20 ^{o}C. The reaction was monitored by UV-Vis absorption spectra (1 cm cuvette). **PQ-OCH$_3$-PY** formation was fitted to an exponential rise to the maximum equation, $y = (y_0\text{-}a)\ e^{kobs*t} + b$, to give k_{obs}.

4.3. Nanosecond Transient Absorption Spectroscopy

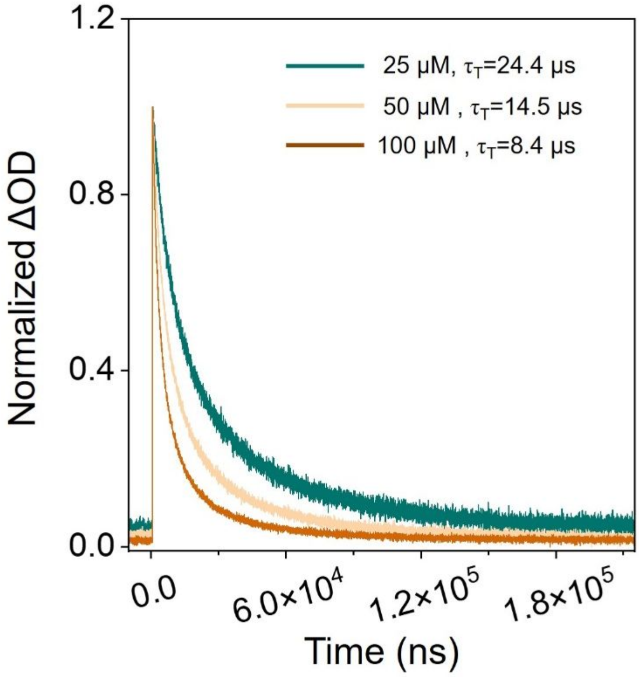

Figure S61. Transient absorption decays of DiIBodipy (at the concentration of 25 μM, 50 μM and 100 μM respectively) at 445 nm. The samples were irradiated at 545 nm.

Figures S62-S69 show heat maps of transient absorption spectra of **PQ-CF$_3$**, **PQ-H**, and **PQ-OCH$_3$** in various solvents together with DADS resulting from their global analysis. Indicated in each case is the optical density of the solution at the employed excitation wavelength and the laser pulse energy.

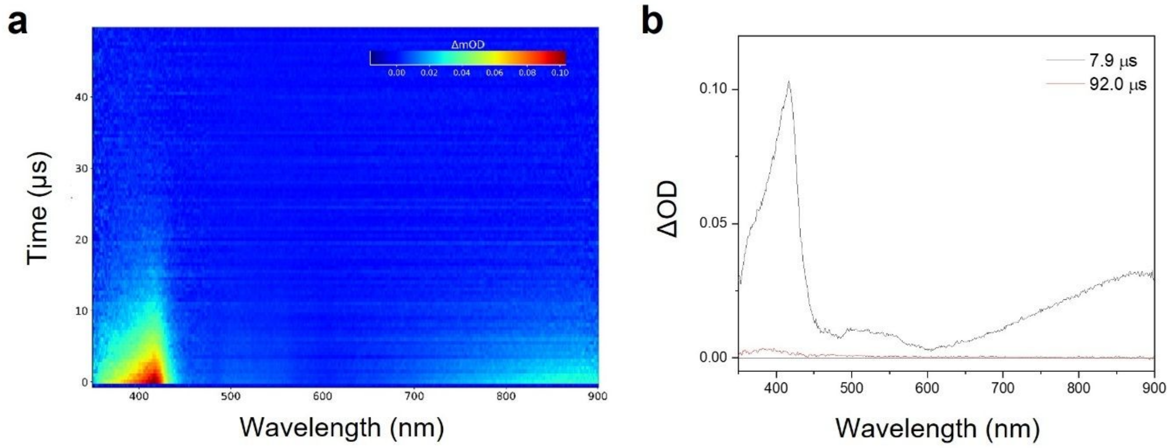

Fig. S62. Transient absorption spectra and DADS **PQ-CF$_3$** in acetonitrile (OD 1.45; 0.1 mJ).

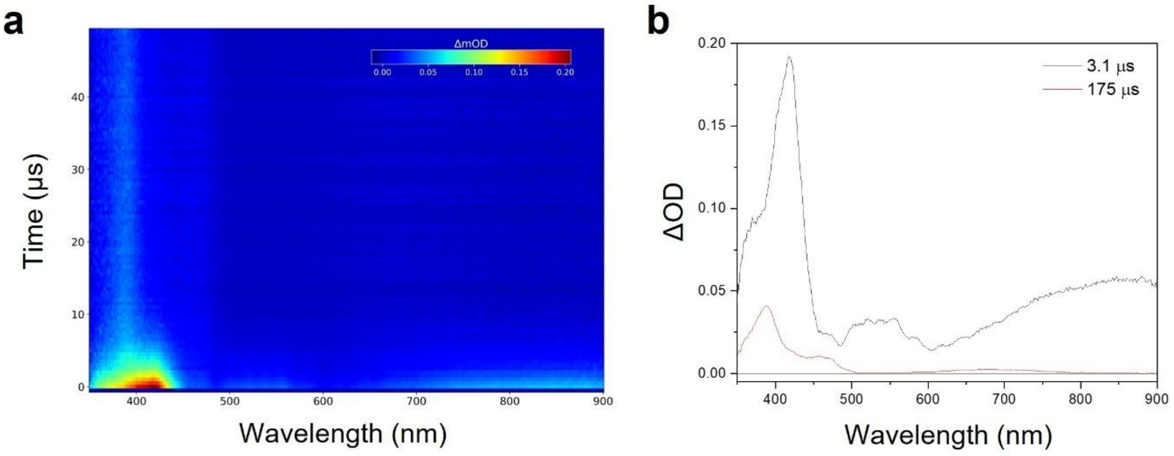

Fig. S63. Transient absorption spectra and DADS **PQ-CF$_3$** in EA (OD 1.56; 0.1 mJ).

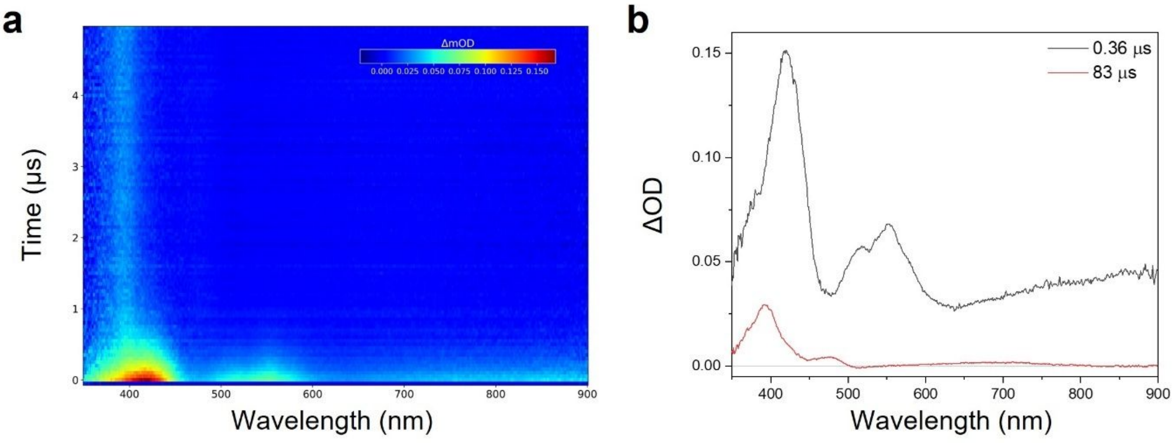

Fig. S64. Transient absorption spectra and DADS **PQ-CF$_3$** in toluene (OD 1.48; 0.1 mJ).

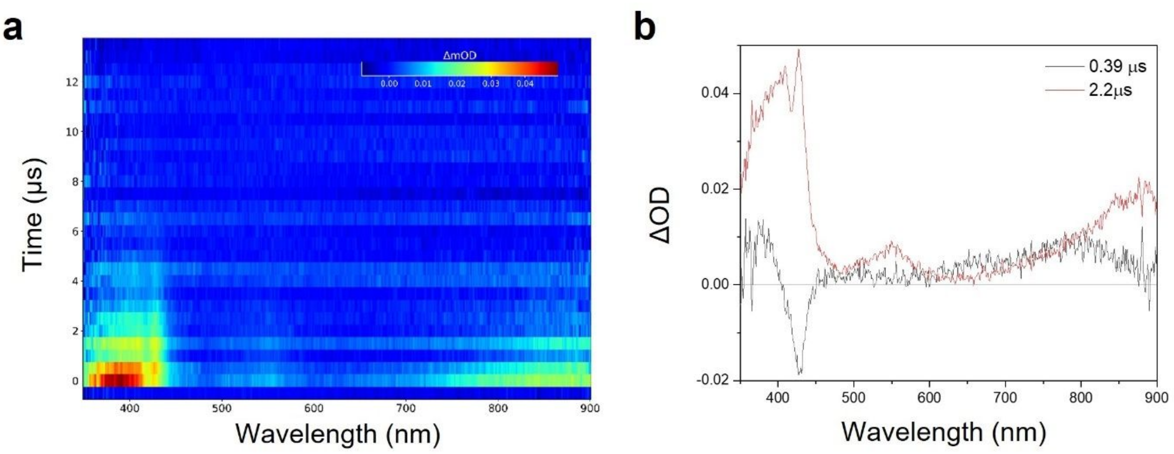

Fig. S65. Transient absorption spectra and DADS **PQ-H** in acetonitrile (OD 0.51; 1.0 mJ).

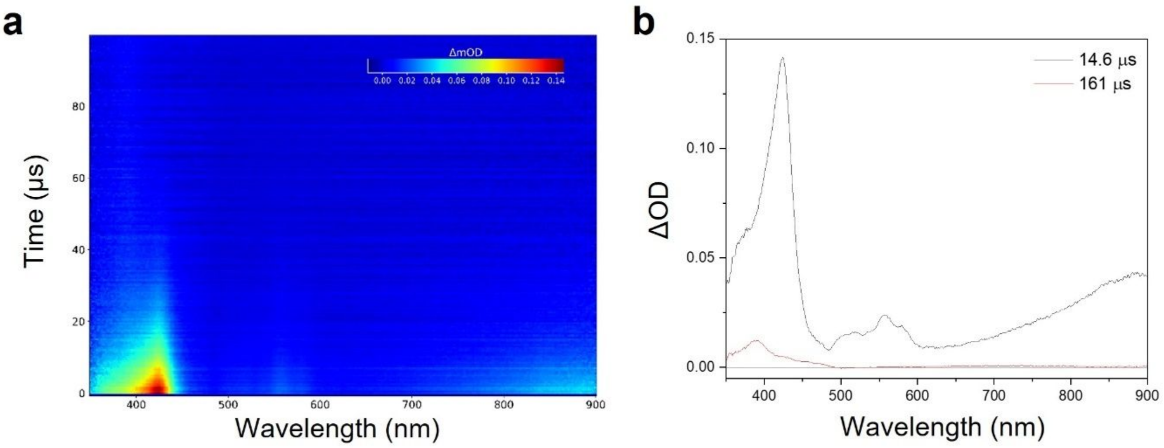

Fig. S66. Transient absorption spectra and DADS **PQ-H** in ethyl acetate (OD 0.98; 0.1 mJ).

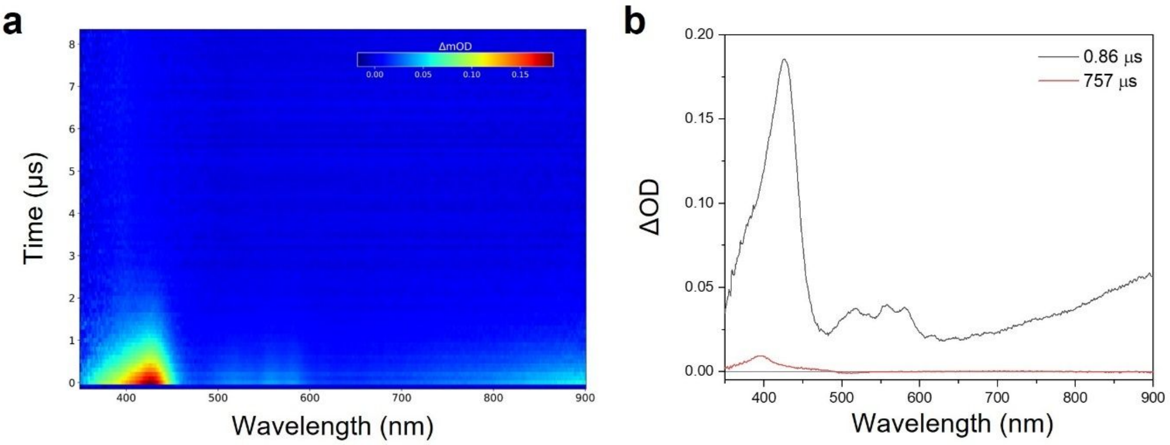

Fig. S67. Transient absorption spectra and DADS **PQ-H** in toluene (OD 1.56; 0.1 mJ).

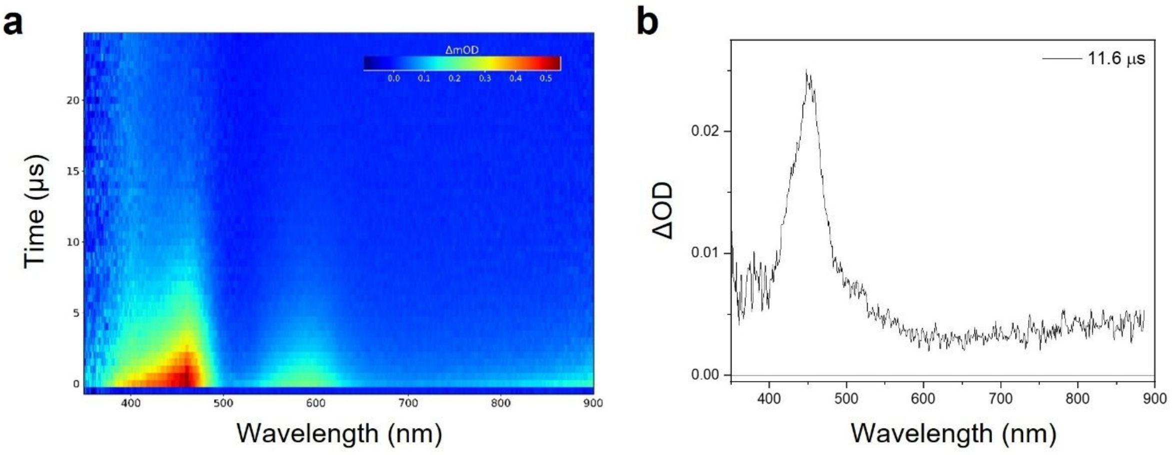

Fig. S68. Transient absorption spectra and DADS **PQ-OCH$_3$** in acetonitrile (OD 0.7; 3.5 mJ).

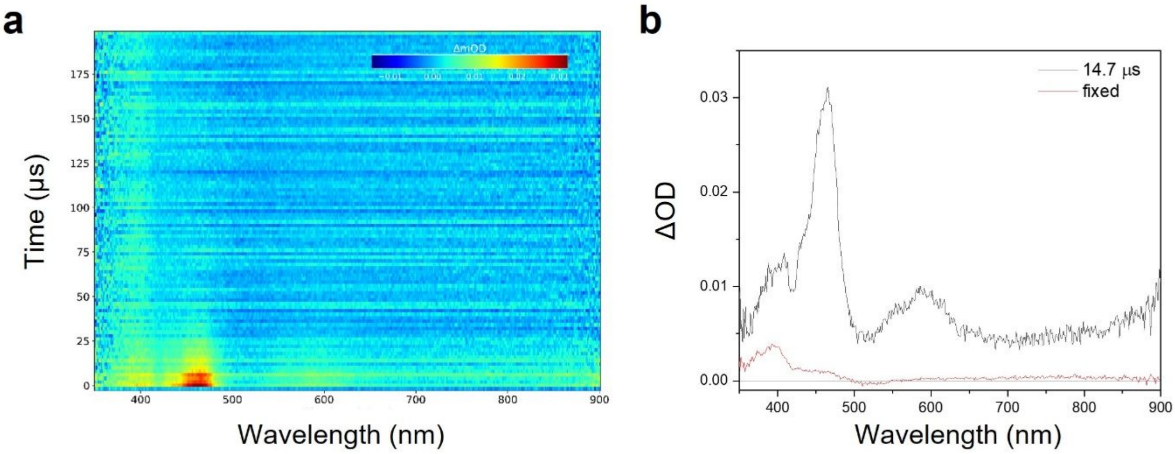

Fig. S69. Transient absorption spectra and DADS **PQ-OCH$_3$** in toluene (OD 0.74; 0.1 mJ). It was not possible to reliably the decay time of the red component, which was therefore kept fixed at ∞.

5. Computational analysis

Table S1. The calculated vertical excitation energies and transition contributions based on the optimized ground-state geometries, calculated at the MN15/Def2-TZVP/PCM(MeCN) level, Gaussian 16.

Compounds	S1		S2		T1		T2	
	Energy level & feature	Transition	Energy level & feature	Transition	Energy level & feature	Transition	Energy level & feature	Transition
PQ-CF_3	2.381 eV (n,π*)	H-2→L (69.6%) H-3→L (18.0%)	2.757 eV (π,π*)	H→L (95.8%)	2.156 eV (n,π*)	H-2→L (68.7%) H-3→L (17.2%)	2.202 eV (π,π*)	H→L (75.4%)
PQ-CHO	2.380 eV (n,π*)	H-4→L (54.6%) H-2→L (32.8%)	2.748 eV (π,π*)	H→L (95.0%)	2.156 eV (n,π*)	H-4→L (53.4%) H-2→L (32.3%)	2.187 eV (π,π*)	H→L (74.0%)
PQ-$COCH_3$	2.381 eV (n,π*)	H-4→L (76.1%) H-5→L (5.2%)	2.724 eV (π,π*)	H→L (95.0%)	2.157 eV (n,π*)	H-4→L (75.0%) H-5→L (4.9%)	2.175 eV (π,π*)	H→L (74.5%)
PQ-H	2.384 eV (n,π*)	H-4→L (86.3%) H-7→L (3.9%)	2.648 eV (π,π*)	H→L (94.9%)	2.135 eV (π,π*)	H→L (77.0%)	2.161 eV (n,π*)	H-4→L (86.1%) H-7→L (3.1%)
PQ-^{t}Bu	2.385 eV (n,π*)	H-4→L (57.8%) H-6→L (22.8%)	2.579 eV (π,π*)	H→L (93.4%)	2.092 eV (π,π*)	H→L (76.5%)	2.163 eV (n,π*)	H-4→L (58.7%) H-6→L (19.7%)
PQ-CH_3	2.385 eV (n,π*)	H-4→L (57.6%) H-6→L (25.1%)	2.571 eV (π,π*)	H→L (93.3%)	2.085 eV (π,π*)	H→L (76.7%)	2.163 eV (n,π*)	H-4→L (58.7%) H-6→L (22.1%)
PQ-OCH_3	2.381 eV (n,π*)	H-6→L (68.0%) H-5→L (11.9%)	2.461 eV (π,π*)	H→L (84.7%)	2.014 eV (π,π*)	H→L (75.5%)	2.164 eV (n,π*)	H-6→L (67.3%) H-5→L (14.6%)

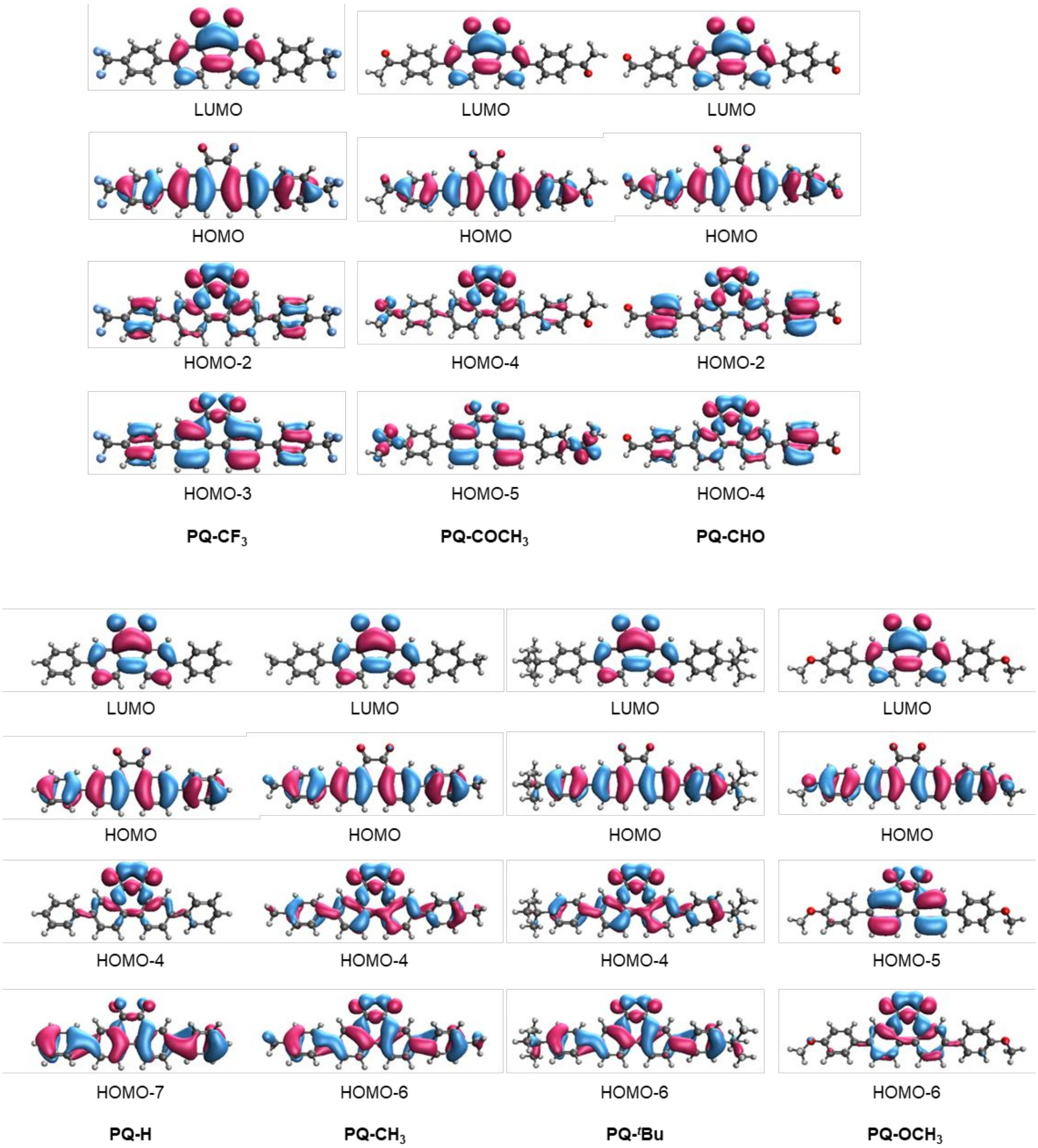

Fig. S70. The frontier molecular orbitals involving vertical excitation energies based on the optimized ground-state geometries, calculated at the MN15/Def2-TZVP/PCM(MeCN) level, Gaussian 16, and visualized via IQmol molecular viewer package.

Table S2. The calculated adiabatic excitation energies and transition contributions based on the optimized lowest singlet-state and triplet-state geometries, respectively, calculated at the MN15/Def2-TZVP/PCM(MeCN) level, Gaussian 16.

Compounds	S1		S2		T1		T2	
	Energy level & feature	Transition	Energy level & feature	Transition	Energy level & feature	Transition	Energy level & feature	Transition
PQ-CF_3	2.325 eV (n,π*)	H-3→L (43.4%)<br>H-5→L (41.4%)	2.623 eV (π,π*)	H→L (90.1%)	1.817 eV (π,π*)	H→L (85.6%)	2.378 eV (n,π*)	H-5→L (85.5%)
PQ-CHO	2.325 eV (n,π*)	H-5→L (50.9%)<br>H-4→L (30.6%)	2.614 eV (π,π*)	H→L (89.8%)	1.812 eV (π,π*)	H→L (85.0%)	2.373 eV (n,π*)	H-7→L (80.2%)
PQ-$COCH_3$	2.324 eV (n,π*)	H-7→L (35.0%)<br>H-6→L (30.7%)	2.590 eV (π,π*)	H→L (85.8%)	1.797 eV (π,π*)	H→L (85.0%)	2.376 eV (n,π*)	H-7→L (77.0%)
PQ-H	2.243 eV (π,π*)	H→L (96.1%)	2.571 eV (n,π*)	H-7→L (81.0%)<br>H-5→L (10.6%)	1.745 eV (π,π*)	H→L (85.8%)	2.385 eV (n,π*)	H-6→L (48.9%)<br>H-5→L (39.7%)
PQ-^{t}Bu	2.177 eV (π,π*)	H→L (95.7%)	2.575 eV (n,π*)	H-7→L (90.9%)	1.704 eV (π,π*)	H→L (85.0%)	2.386 eV (n,π*)	H-6→L (87.0%)
PQ-CH_3	2.166 eV (π,π*)	H→L (95.8%)	2.576 eV (n,π*)	H-7→L (90.9%)	1.696 eV (π,π*)	H→L (85.1%)	2.388 eV (n,π*)	H-6→L (87.4%)
PQ-OCH_3	2.048 eV (π,π*)	H→L (95.0%)	2.586 eV (n,π*)	H-7→L (92.1%)	1.623 eV (π,π*)	H→L (84.1%)	2.392 eV (n,π*)	H-6→L (89.5%)

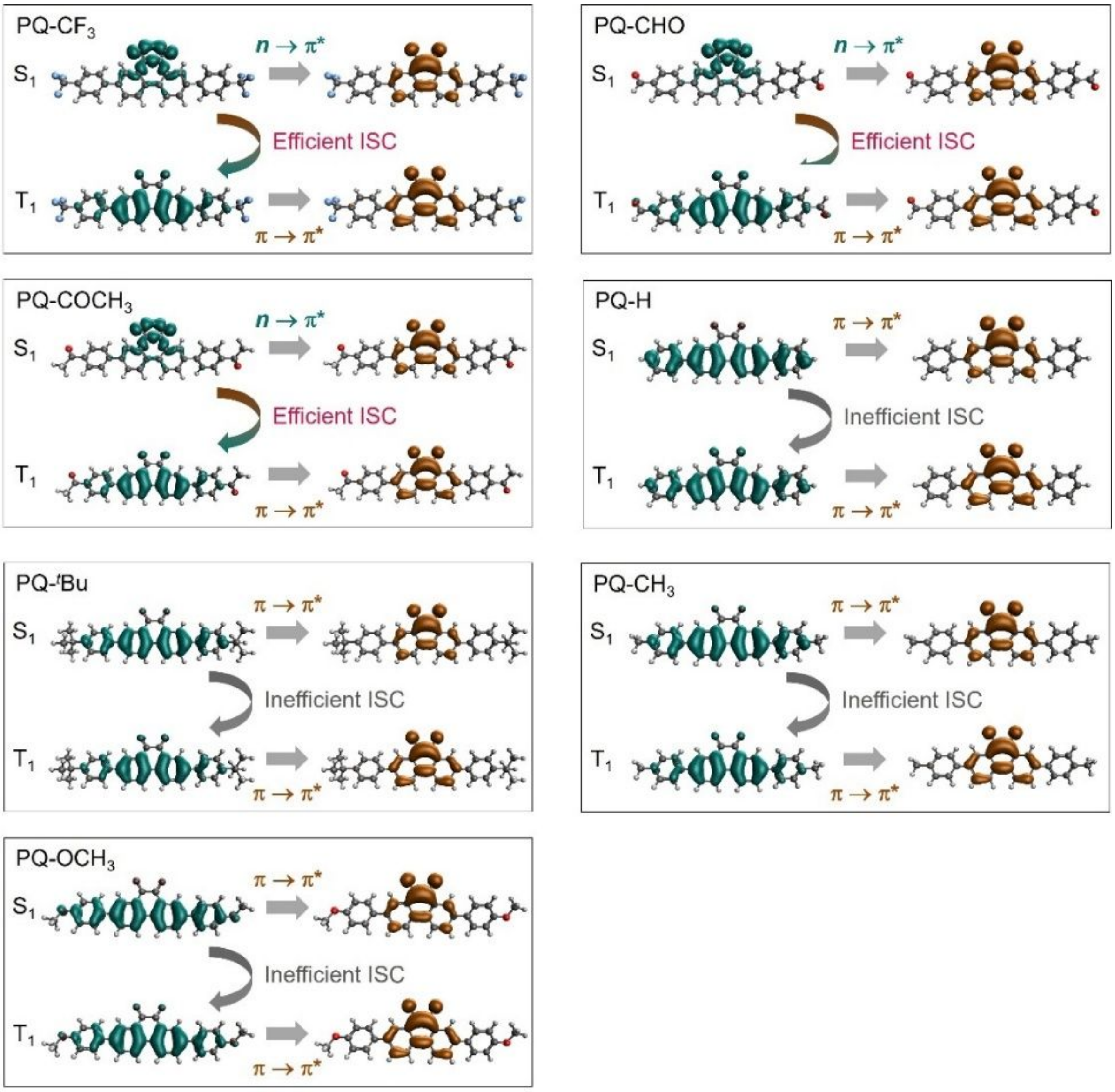

Fig. S71. Hole-electron analysis of all compounds based on their optimized singlet-state and triplet-state geometries. The hole and electron parts shown in green and brown color, respectively. The different electronic characters of the lowest singlet and triplet state promote efficient $S_1 \rightarrow T_1$ intersystem crossing (ISC) while the same features of them diminish the ISC efficiency.

Table S2. The calculated adiabatic excitation energies and transition contributions of **PQ-CF$_3$**, **PQ-H**, and **PQ-OCH$_3$** based on their optimized lowest singlet-state and triplet-state geometries, respectively, calculated at the MN15/Def2-TZVP/PCM level with different solvent environments, Gaussian 16.

Compounds		S1		S2		T1		T2	
		Energy level & feature	Transition	Energy level & feature	Transition	Energy level & feature	Transition	Energy level & feature	Transition
PQ-CF$_3$	Toluene	2.301 eV (n,π^*)	H-2→L (89.4%)	2.870 eV (π,π^*)	H→L (95.7%)	2.072 eV (n,π^*)	H-2→L (87.7%)	2.269 eV (π,π^*)	H→L (72.1%)
	EA	2.347 eV (n,π^*)	H-2→L (87.8%)	2.805 eV (π,π^*)	H→L (95.8%)	2.120 eV (n,π^*)	H-2→L (86.3%)	2.232 eV (π,π^*)	H→L (74.0%)
	ACN	2.381 eV (n,π^*)	H-2→L (69.6%) H-3→L (18.0%)	2.757 eV (π,π^*)	H→L (95.8%)	2.156 eV (n,π^*)	H-2→L (68.7%) H-3→L (17.2%)	2.202 eV (π,π^*)	H→L (75.4%)
PQ-H	Toluene	2.308 eV (n,π^*)	H-4→L (82.1%)	2.764 eV (π,π^*)	H→L (95.1%)	2.080 eV (n,π^*)	H-4→L (80.7%)	2.212 eV (π,π^*)	H→L (73.8%)
	EA	2.352 eV (n,π^*)	H-4→L (88.0%)	2.697 eV (π,π^*)	H→L (95.0%)	2.127 eV (n,π^*)	H-4→L (86.6%)	2.167 eV (π,π^*)	H→L (75.7%)
	ACN	2.384 eV (n,π^*)	H-4→L (86.3%) H-7→L (3.9%)	2.648 eV (π,π^*)	H→L (94.9%)	2.135 eV (π,π^*)	H→L (77.0%)	2.161 eV (n,π^*)	H-4→L (86.1%) H-7→L (3.1%)
PQ-OCH$_3$	Toluene	2.352 eV (n,π^*)	H-5→L (69.0%) H-4→L (17.8%)	2.509 eV (π,π^*)	H→L (90.6%)	2.081 eV (n,π^*)	H-3→L (56.9%) H-5→L (16.9%)	2.108 eV (π,π^*)	H→L (64.5%)
	EA	2.352 eV (n,π^*)	H-5→L (69.0%) H-4→L (17.8%)	2.509 eV (π,π^*)	H→L (90.6%)	2.051 eV (π,π^*)	H→L (74.1%)	2.130 eV (n,π^*)	H-5→L (65.7%) H-4→L (19.4%)
	ACN	2.381 eV (n,π^*)	H-6→L (68.0%) H-5→L (11.9%)	2.461 eV (π,π^*)	H→L (84.7%)	2.014 eV (π,π^*)	H→L (75.5%)	2.164 eV (n,π^*)	H-6→L (67.3%) H-5→L (14.6%)

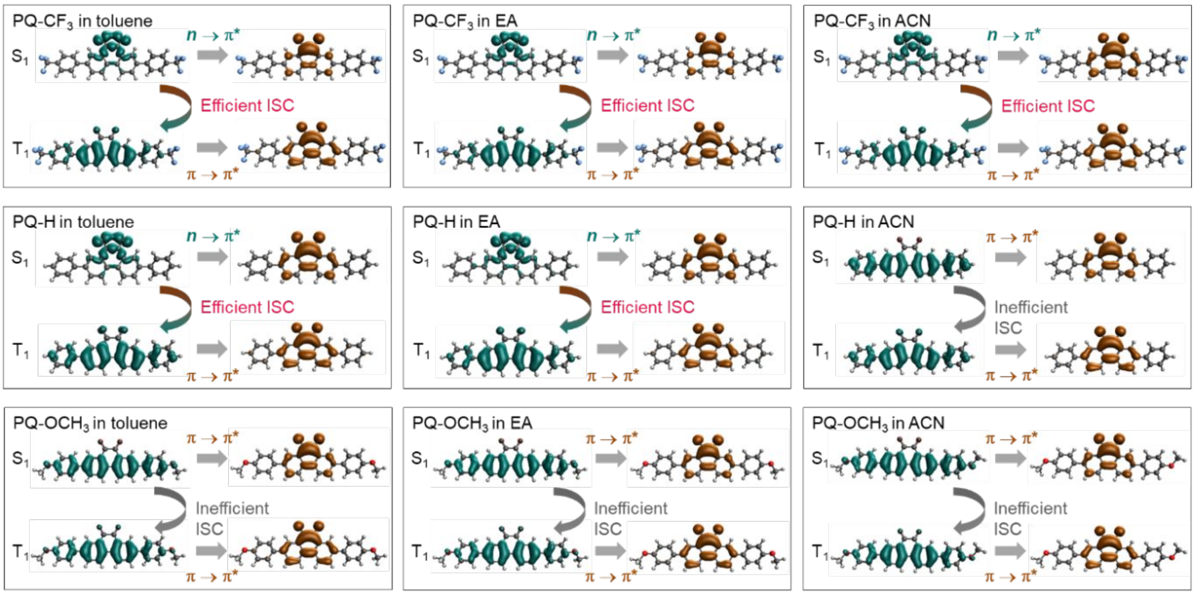

Fig. S72. Hole-electron analysis of **PQ-CF$_3$**, **PQ-H,** and **PQ-OCH$_3$** in different solvent environments based on their optimized singlet-state and triplet-state geometries. The hole and

electron parts shown in green and brown color, respectively. The different electronic characters of the lowest singlet and triplet state promote efficient $S_1 \rightarrow T_1$ intersystem crossing (ISC) while the same features of them diminish the ISC efficiency.

6. References

(1) Fu, Y.; Alachouzos, G.; Simeth, N. A.; Di Donato, M.; Hilbers, M. F.; Buma, W. J.; Szymanski, W.; Feringa, B. L. Triplet‐Triplet Energy Transfer: A Simple Strategy for an Efficient Visible Light‐Induced Photoclick Reaction. *Angew. Chem. Int. Ed.* **2024**, *63* (21), e202319321. https://doi.org/10.1002/anie.202319321.

(2) Fu, Y.; Alachouzos, G.; Simeth, N. A.; Di Donato, M.; Hilbers, M. F.; Buma, W. J.; Szymanski, W.; Feringa, B. L. Establishing PQ-ERA Photoclick Reactions with Unprecedented Efficiency by Engineering of the Nature of the Phenanthraquinone Triplet State. *Chem. Sci.* **2023**, *14* (27), 7465–7474. https://doi.org/10.1039/D3SC01760E.

(3) Doze, A. M.; Fu, Y.; Di Donato, M.; Hilbers, M. F.; Luurtsema, G.; Elsinga, P. H.; Buma, W. J.; Szymanski, W.; Feringa, B. L. With or without a Co-Solvent? Highly Efficient Ultrafast Phenanthrenequinone-Electron Rich Alkene (PQ-ERA) Photoclick Reactions. *Chem. Sci.* **2024**, *15* (29), 11557–11563. https://doi.org/10.1039/D4SC01810A.

(4) Snellenburg, J. J.; Laptenok, S. P.; Seger, R.; Mullen, K. M.; Stokkum, I. H. M. van. Glotaran : A Java -Based Graphical User Interface for the R Package TIMP. *J. Stat. Softw.* **2012**, *49* (3), 1–22. https://doi.org/10.18637/jss.v049.i03.

(5) Yu, H. S.; He, X.; Li, S. L.; Truhlar, D. G. MN15: A Kohn–Sham Global-Hybrid Exchange–Correlation Density Functional with Broad Accuracy for Multi-Reference and Single-Reference Systems and Noncovalent Interactions. *Chem. Sci.* **2016**, *7* (8), 5032–5051. https://doi.org/10.1039/C6SC00705H.

(6) Mennucci, B. Polarizable Continuum Model. *WIREs Comput. Mol. Sci.* **2012**, *2* (3), 386–404. https://doi.org/10.1002/wcms.1086.

(7) Frisch, M. J.; Trucks, G. W.; Schlegel, H. B.; Scuseria, G. E.; Robb, M. A.; Cheeseman, J. R.; Scalmani, G.; Barone, V.; Petersson, G. A.; Nakatsuji, H. *Gaussian 16 Revision a. 03. 2016; Gaussian Inc*; 2016.

(8) Lu, T.; Chen, F. Multiwfn: A Multifunctional Wavefunction Analyzer. *J. Comput. Chem.* **2012**, *33* (5), 580–592. https://doi.org/10.1002/jcc.22885.

(9) Liu, Z.; Lu, T.; Chen, Q. An Sp-Hybridized All-Carboatomic Ring, Cyclo[18]Carbon: Electronic Structure, Electronic Spectrum, and Optical Nonlinearity. *Carbon N. Y.* **2020**, *165*, 461–467. https://doi.org/10.1016/j.carbon.2020.05.023.

(10) Shao, Y.; Gan, Z.; Epifanovsky, E.; Gilbert, A. T. B.; Wormit, M.; Kussmann, J.; Lange, A. W.; Behn, A.; Deng, J.; Feng, X.; Ghosh, D.; Goldey, M.; Horn, P. R.; Jacobson, L. D.; Kaliman, I.; Khaliullin, R. Z.; Kuś, T.; Landau, A.; Liu, J.; Proynov, E. I.; Rhee, Y. M.; Richard, R. M.; Rohrdanz, M. A.; Steele, R. P.; Sundstrom, E. J.; Woodcock, H. L.; Zimmerman, P. M.; Zuev, D.; Albrecht, B.; Alguire, E.; Austin, B.; Beran, G. J. O.; Bernard, Y. A.; Berquist, E.; Brandhorst, K.; Bravaya, K. B.; Brown, S. T.; Casanova, D.; Chang, C.-M.; Chen, Y.; Chien, S. H.; Closser, K. D.; Crittenden, D. L.; Diedenhofen, M.; DiStasio, R. A.; Do, H.; Dutoi, A. D.; Edgar, R. G.; Fatehi, S.; Fusti-Molnar, L.; Ghysels, A.; Golubeva-Zadorozhnaya, A.; Gomes, J.; Hanson-Heine,

M. W. D.; Harbach, P. H. P.; Hauser, A. W.; Hohenstein, E. G.; Holden, Z. C.; Jagau, T.-C.; Ji, H.; Kaduk, B.; Khistyaev, K.; Kim, J.; Kim, J.; King, R. A.; Klunzinger, P.; Kosenkov, D.; Kowalczyk, T.; Krauter, C. M.; Lao, K. U.; Laurent, A. D.; Lawler, K. V.; Levchenko, S. V.; Lin, C. Y.; Liu, F.; Livshits, E.; Lochan, R. C.; Luenser, A.; Manohar, P.; Manzer, S. F.; Mao, S.-P.; Mardirossian, N.; Marenich, A. V.; Maurer, S. A.; Mayhall, N. J.; Neuscamman, E.; Oana, C. M.; Olivares-Amaya, R.; O'Neill, D. P.; Parkhill, J. A.; Perrine, T. M.; Peverati, R.; Prociuk, A.; Rehn, D. R.; Rosta, E.; Russ, N. J.; Sharada, S. M.; Sharma, S.; Small, D. W.; Sodt, A.; Stein, T.; Stück, D.; Su, Y.-C.; Thom, A. J. W.; Tsuchimochi, T.; Vanovschi, V.; Vogt, L.; Vydrov, O.; Wang, T.; Watson, M. A.; Wenzel, J.; White, A.; Williams, C. F.; Yang, J.; Yeganeh, S.; Yost, S. R.; You, Z.-Q.; Zhang, I. Y.; Zhang, X.; Zhao, Y.; Brooks, B. R.; Chan, G. K. L.; Chipman, D. M.; Cramer, C. J.; Goddard, W. A.; Gordon, M. S.; Hehre, W. J.; Klamt, A.; Schaefer, H. F.; Schmidt, M. W.; Sherrill, C. D.; Truhlar, D. G.; Warshel, A.; Xu, X.; Aspuru-Guzik, A.; Baer, R.; Bell, A. T.; Besley, N. A.; Chai, J.-D.; Dreuw, A.; Dunietz, B. D.; Furlani, T. R.; Gwaltney, S. R.; Hsu, C.-P.; Jung, Y.; Kong, J.; Lambrecht, D. S.; Liang, W.; Ochsenfeld, C.; Rassolov, V. A.; Slipchenko, L. V.; Subotnik, J. E.; Van Voorhis, T.; Herbert, J. M.; Krylov, A. I.; Gill, P. M. W.; Head-Gordon, M. Advances in Molecular Quantum Chemistry Contained in the Q-Chem 4 Program Package. *Mol. Phys.* **2015**, *113* (2), 184–215. https://doi.org/10.1080/00268976.2014.952696.

(11) Lin, H. Y.; Huang, W. C.; Chen, Y. C.; Chou, H. H.; Hsu, C. Y.; Lin, J. T.; Lin, H. W. BODIPY Dyes with β-Conjugation and Their Applications for High-Efficiency Inverted Small Molecule Solar Cells. *Chem. Commun.* **2012**, *48* (71), 8913–8915. https://doi.org/10.1039/c2cc34286c.

(12) Bonnier, C.; MacHin, D. D.; Abdi, O.; Koivisto, B. D. Manipulating Non-Innocent π-Spacers: The Challenges of Using 2,6-Disubstituted BODIPY Cores within Donor-Acceptor Light-Harvesting Motifs. *Org. Biomol. Chem.* **2013**, *11* (22), 3756–3760. https://doi.org/10.1039/c3ob40213d.

(13) Lang, K.; Davis, L.; Wallace, S.; Mahesh, M.; Cox, D. J.; Blackman, M. L.; Fox, J. M.; Chin, J. W. Genetic Encoding of Bicyclononynes and Trans -Cyclooctenes for Site-Specific Protein Labeling in Vitro and in Live Mammalian Cells via Rapid Fluorogenic Diels–Alder Reactions. *J. Am. Chem. Soc.* **2012**, *134* (25), 10317–10320. https://doi.org/10.1021/ja302832g.

(14) Li, J.; Kong, H.; Huang, L.; Cheng, B.; Qin, K.; Zheng, M.; Yan, Z.; Zhang, Y. Visible Light-Initiated Bioorthogonal Photoclick Cycloaddition. *J. Am. Chem. Soc.* **2018**, *140* (44), 14542–14546. https://doi.org/10.1021/jacs.8b08175.

(15) Svejstrup, T. D.; Chatterjee, A.; Schekin, D.; Wagner, T.; Zach, J.; Johansson, M. J.; Bergonzini, G.; König, B. Effects of Light Intensity and Reaction Temperature on Photoreactions in Commercial Photoreactors. *ChemPhotoChem* **2021**, *5* (9), 808–814. https://doi.org/10.1002/cptc.202100059.

(16) Fu, Y.; Helbert, H.; Simeth, N. A.; Crespi, S.; Spoelstra, G. B.; van Dijl, J. M.; van Oosten, M.; Nazario, L. R.; van der Born, D.; Luurtsema, G.; Szymanski, W.; Elsinga, P. H.; Feringa, B. L. Ultrafast Photoclick Reaction for Selective 18 F-Positron Emission Tomography Tracer Synthesis in Flow. *J. Am. Chem. Soc.* **2021**, *143* (27), 10041–10047. https://doi.org/10.1021/jacs.1c02229.

(17) Fu, Y.; Simeth, N. A.; Toyoda, R.; Brilmayer, R.; Szymanski, W.; Feringa, B. L. Molecular Engineering To Enhance Reactivity and Selectivity in an Ultrafast Photoclick Reaction. *Angew. Chem. Int. Ed.* **2023**, *62* (16), e202218203. https://doi.org/10.1002/anie.202218203.
